# The role of Jagged1 as a dynamic switch of cancer cell plasticity in PDAC assembloids

**DOI:** 10.7150/thno.71364

**Published:** 2022-05-24

**Authors:** Jae-Il Choi, John Hoon Rim, Sung Ill Jang, Joon Seong Park, Hak Park, Jae Hee Cho, Jong-Baeck Lim

**Affiliations:** 1 Department of Laboratory Medicine, Severance Hospital, Yonsei University College of Medicine, Seoul, Republic of Korea; 2 Institute of Gastroenterology, Department of Internal Medicine, Gangnam Severance Hospital, Yonsei University College of Medicine, Seoul, Republic of Korea; 3 Pancreatobiliary Cancer Clinic, Department of Surgery, Gangnam Severance Hospital, Yonsei University College of Medicine, Seoul, Republic of Korea

**Keywords:** Human cancer organoid, cancer-initiating cells, plasticity, tumor microenvironment

## Abstract

**Background:** Pancreatic ductal adenocarcinoma (PDAC), which commonly relapses due to chemotherapy resistance, has a poor 5-year survival rate (< 10%). The ability of PDAC to dynamically switch between cancer-initiating cell (CIC) and non-CIC states, which is influenced by both internal and external events, has been suggested as a reason for the low drug efficacy. However, cancer cell plasticity using patient-derived PDAC organoids remains poorly understood.

**Methods:** First, we successfully differentiated CICs, which were the main components of PDAC organoids, toward epithelial ductal carcinomas. We further established PDAC assembloids of organoid-derived differentiated ductal cancer cells with endothelial cells (ECs) and autologous immune cells. To investigate the mechanism for PDAC plasticity, we performed single-cell RNA sequencing analysis after culturing the assembloids for 7 days.

**Results:** In the PDAC assembloids, the ECs and immune cells acted as tumor-supporting cells and induced plasticity in the differentiated ductal carcinomas. We also observed that the transcriptome dynamics during PDAC re-programming were related to the WNT/beta-catenin pathway and apoptotic process. Interestingly, we found that WNT5B in the ECs was highly expressed by trans interaction with a JAG1. Furthermore, JAG1 was highly expressed on PDAC during differentiation, and NOTCH1/NOTCH2 were expressed on the ECs at the same time. The WNT5B expression level correlated positively with those of JAG1, NOTCH1, and NOTCH2, and high JAG1 expression correlated with poor survival. Additionally, we experimentally demonstrated that neutralizing JAG1 inhibited cancer cell plasticity.

**Conclusions:** Our results indicate that JAG1 on PDAC plays a critical role in cancer cell plasticity and maintenance of tumor heterogeneity.

## Introduction

Pancreatic cancer, particularly pancreatic ductal adenocarcinoma (PDAC), is an aggressive cancer type with a 5-year survival rate of < 10%, and it is frequently detected at an advanced stage 1. Systemic chemotherapy combinations, such as gemcitabine plus nab-paclitaxel or FOLFIRINOX (folinic acid, 5-fluorouracil, irinotecan, and oxaliplatin), have shown improved overall survival compared to gemcitabine alone 2. Nonetheless, relapses caused by chemotherapeutic drug resistance remain common in the real world 1, and multiple studies have reported that tumor heterogeneity drives drug resistance 3. Especially, cancer-initiating cells (CICs), also called cancer stem cells (CSCs), have self-renewal and differentiation capacities that enable them to recapitulate the cellular heterogeneity of the original tumor 4,5.

Targeting CICs by inhibiting EPCAM 6, Hedgehog, NOTCH/DLL4, Wnt, and TGFB signaling has improved the therapeutic efficacy in xenotransplantation models 4. However, those treatments failed in clinical trials due to their severe toxicity and low efficacy 5. Their low efficacy could have resulted from the cancer's ability to dynamically switch between CSC and non-CSC states under the regulation of both internal and external influences 7. Therefore, the development of therapeutic agents that target cancer cell plasticity is essential to promote a complete response to chemo-drugs.

The activation of NOTCH signaling through cell-to-cell signaling in normal tissues 8 and cancer 9 sparks the acquisition of specific cell fates and cell growth. NOTCH ligand members are generally divided according to the presence or absence of the N-terminal DSL (Delta/Serrate/LAG-2) motif and specialized tandem EGF repeats, called the DOS (Delta and OSM-11-like proteins) domain. As canonical NOTCH ligand members, DLL1, JAG1, and JAG2 have DOS domain-containing DSL ligands, whereas DLL3 and DLL4 have DSL ligands that lack the DOS domain 8,10. In a mouse model of pancreatic cancer, NOTCH inhibition in epithelial cells inhibited pancreatic intraepithelial neoplasia (PanIN) formation 11. Also, NOTCH2 is reported to be necessary for PanIN and PDAC development 12. These findings suggest that NOTCH signaling plays an important oncogenic role in pancreatic cancer cells. However, the role of JAG1 expressed in PDAC organoids for cancer cell plasticity remains poorly understood. In this study, we established organoid-derived PDAC assembloids that contained endothelial cells and autologous immune cells, and identified JAG1 as a dynamic switch for cancer plasticity in PDAC assembloids.

## Materials and Methods

### Human specimens

Cancer tissues from either surgical samples or endoscopic ultrasound-guided fine needle aspiration (EUS-FNA) samples, and peripheral blood mononuclear cells (PBMCs) paired with organoids from the same pancreatic cancer patients were obtained from Severance Hospital, Yonsei University College of Medicine. All human samples and experiments were approved by the institutional review board (IRB) of Sinchon/Gangnam Severance Hospital. Informed consent was obtained from all donors according to the IRB guidelines. Donor information is provided in **Table S1.**

### Establishment of human pancreatic cancer organoids and differentiation

The isolation and culture of pancreatic cancer organoids was performed as previously described 13. Human pancreatic cancer organoids were cultured in AdDMEM/F12 medium supplemented with GlutaMAX (Thermo Fisher Scientific), penicillin/streptomycin (Thermo Fisher Scientific), B27 (Thermo Fisher Scientific), N-acetyl-L-cysteine (1 mM, Sigma-Aldrich), Wnt3a-conditioned medium (50% v/v), RSPO1-conditioned medium (10% v/v, R&D Systems), recombinant noggin protein (100 ng/ml, PeproTech), recombinant epidermal growth factor protein (EGF, 50 ng/mL, PeproTech), gastrin (10 nM, Sigma-Aldrich), recombinant fibroblast growth factor 10 protein (FGF10, 100 ng/mL, PeproTech), nicotinamide (10 mM, Sigma-Aldrich), and A83-01 (0.5 μM, Tocris, Bristol, UK).

For ductal-like cancer cell differentiation, organoids were washed with basal medium and then cultured in AdDMEM/F12 with 50 ng/ml EGF, Wnt-C59 (100 nM, Tocris), DAPT (20 μM, Sigma-Aldrich), and B27 for 2 days.

### Freezing and isolation of PBMCs

As previously described 14, the PBMCs from PDAC-organoid paired blood were isolated using Ficoll-Paque. The isolated PBMCs were cryopreserved in fetal bovine serum (FBS) with 10% dimethyl sulfoxide until analysis was performed.

### Human umbilical vein endothelial cell (HUVEC) culture

On 1% gelatin-coated dishes, HUVECs (Lonza, Basel, Switzerland) were cultured in EBM-2 basal medium (Lonza, CC-3156) supplemented with an EGM-2MV SingleQuots Supplement Pack (Lonza, CC-4147).

### Co-culture of PDAC organoids with HUVECs and autologous immune cells

Differentiated PDAC organoids were dissociated using TrypLE™ Express (Thermo Fisher Scientific, 12605010), and organoid-derived CD44(-) cancer cells were stained for CD44-APCcy7 and sorted using flow cytometry (FACS Aria III). Sorting purity is presented in **Figure S1A**. For cell tracing in the flow cytometry analysis, HUVECs and PBMCs were stained using carboxyfluorescein succinimidyl ester (CFSE) according to the manufacturer's instructions. Organoid-derived differentiated CD44(-) cancer cells, HUVECs, and PBMCs paired with organoids were mixed at a 1:2:2 ratio and cultured on round-bottom ultra-low attachment plates (Corning) in growth medium supplemented with 10% Matrigel for 24 h. Then, the mixtures were cultured in the basal medium (AdDMEM/F12 with GlutaMax and 5% FBS) for 7 days, as previously described 13.

### Flow cytometry

A flow cytometry analysis was performed as previously described 13. Briefly, cells dissociated from assembloids and tumor masses by enzyme were stained with antibodies specific for CD44-APCcy7 (BioLegend, San Diego, CA, USA), CD24-Pecy7 (BioLegend), EpCAM-PerCPcy5.5 (BioLegend), and JAG1-PE (BD Bioscience). The flow cytometry analysis was performed using LSR II, and data were analyzed using FlowJo and DIVA software.

### Xenotransplantation

Animal experimental procedures were approved by the Institutional Animal Care and Use Committee of Yonsei University College of Medicine. Cells dissociated from two PDAC assembloids were transplanted into the NSG-(*K^b^D^b^*)^null^ (*IA*)^null^ (The Jackson Laboratory) mice through subcutaneous injection with 50% Matrigel. After 6 weeks, the tumors were excised and embedded into paraffin blocks or frozen. Immunofluorescence and hematoxylin and eosin (H&E) staining were performed on the frozen and paraffin sections.

### Immunofluorescence staining

Freshly obtained tumor tissues were fixed using 2% paraformaldehyde, and then infiltrated with sucrose and embedded using the OCT compound. For immunostaining, the sections were blocked with bovine serum albumin and stained with anti-CD31, anti-CD44, and anti-CD24. The samples were mounted using fluorescent mounting solution (DAKO).

To assess the differentiation capacity of the organoids, differentiated organoids and control organoids were stained as previously described, with minor modification 13. In brief, the differentiated and control organoids were fixed in paraformaldehyde and glutaraldehyde for 10 min. Then the organoids were washed with phosphate buffered saline containing 10 mM NaBH4 and stained with anti-carbonic anhydrase II (CA2) (Santa Cruz Biotechnology). The organoids were stained with a secondary antibody and 4,6-diamidino-2-phenylindole. Fluorescence images of the stained organoids and frozen sections were obtained using a Zeiss LSM710 laser scanning confocal microscope.

### Single-cell RNA-seq

PDAC organoids were differentiated into ductal-like epithelium cancer cells in the differentiation medium containing Wnt-C59/DAPT. After staining the samples for CD44-APCcy7 (BioLegend) and Propidium iodide (PI) (Sigma-Aldrich), the CD44(-)PI(-) cells were isolated via FACS sorting (FACS Aria III). After the diPDAC assembloids (organoid-derived differentiated PDAC with EC/autologous immune cells) were established, they were cultured for 7 days in the basal medium. After dissociation using TrypLE™ Express, dead cells were removed using a dead cell removal kit (Miltenyi Biotec) according to the manufacturer's instructions. Cell number and viability were measured using trypan blue stain and a Countess Cell Counter (T10282, Thermo Fisher). To obtain libraries, we used the Chromium Next GEM Single Cell 3' Reagent Kits (v3.1, 1000121) according to the manufacturer's instructions. Libraries were sequenced using Novaseq 6000.

For single-cell analyses, Cell Ranger (v3.1) software was used to perform read trimming and alignment through the GRCh38-3.0.0 reference genome. For filtering, normalization, and clustering, we used the standard analysis pipeline in the R package Seurat (v4.0.3) 15. In brief, we filtered the cells that expressed fewer than 200 genes, more than 10,000 genes, or more than 20% mitochondrial genes. After a cell-cycle regression, the filtered data were normalized using the Seurat function SCTransform 16. Cell type annotation used the Seurat functions “FindMarkers” and “PanglaoDB” 17. The annotated T cell population was reanalyzed using the “DatabaseImmuneCellExpressionData” function from the R package SingleR (v1.4.1) 18. To investigate the plasticity between CICs and differentiated ductal-like cancer cells, we performed a trajectory analysis in the PDAC cluster according to the standard analysis pipeline of the R package Monocle (v2.18.0) 19. Briefly, the cell fate in the PDAC cluster was identified by CD44 and CA2 level using the “setOrderingFilter” function, followed by “reduceDimension” and “clusterCells” functions 19. T-test analysis was done to identify differentially expressed genes among each state (1, 2, and 3) with statistical significance. All 4,333 genes are shown in **Table S2.** These genes were analyzed for gene ontology enrichment using the Database for Annotation, Visualization and Integrated Discovery (DAVID) 20. All significant interactions (L-R pairs) between PDACs and ECs were analyzed using the Cell-Cell Contact database from R package CellChat 21.

### TCGA and GTEX dataset analysis

The Pancreatic Adenocarcinoma (PAAD) dataset of The Cancer Genome Atlas (TCGA) and the normal pancreas dataset from Genotype-Tissue Expression (GTEX) were analyzed using the R package recount3 22 and TCGAbiolinks 23. The overall survival (OS) of TCGA was analyzed through Gene Expression Profiling Interactive Analysis (GEPIA) 24. To validate the differences for transcriptome between normal pancreas and tumor samples, analysis was performed using the R package caret, a machine learning technique 25. Normal and tumor samples divided the data into 75% of the sample for training and 25% for testing using the createDataPartition, and then performed train using the Elastic Net model.

### qRT-PCR

Culture plates were coated with 10 μg/ml of recombinant human JAG1 N-terminal Fc chimera protein (R&D Systems, 10111-JG-050) at 4°C overnight. Then, HUVECs were seeded in the coated dishes. After 24 h, total RNA was extracted using an RNeasy Plus Mini Kit (QIAGEN) according to the manufacturer's instructions, and cDNA was synthesized using a GoScript™ reverse transcription system (Promega). qRT-PCR was performed using GoTaq qPCR Master Mix (Promega) and a StepOne™ Real-Time PCR System (Thermo Fisher Scientific). The primers used are listed in **Table S3.**

### Neutralizing JAG1

Organoid-derived CD44(-)PI(-) differentiated ductal-like cancer cells were sorted using flow cytometry (FACS Aria III). Then, we established them with CFSE-labeled endothelial cells in round-bottom ultra-low attachment plates (Corning) and cultured them with 10 μg/ml of JAG1-neutralizing antibody (R&D Systems, MAB12771) and control mouse IgG2b (R&D Systems, MAB004). After 1 day, the assembloids were washed and then embedded in GFR Matrigel and cultured in the basal medium containing JAG1-neutralizing antibody or mouse IgG2b. After 6 days, the assembloids were subjected to a FACS analysis to determine the CIC population.

### Statistical analysis

Error bars for all data are presented as the mean ± standard error of the mean. Appropriate statistical methods were utilized for different analyses. Briefly, statistical comparisons of two samples were performed using Mann-Whitney *U* testing and T-test. To compare multiple samples, statistical significance was assessed using the Dunn, Tukey, and Dunnett multiple comparisons tests. A *P* value < 0.05 was considered statistically significant. Statistical tests were performed using SPSS version 25 (SPSS Inc, Chicago, IL, USA), GraphPad Prism 8, and R.

## Results

### CICs in the PDAC organoids were differentiated toward ductal carcinomas

**Figure 1:**
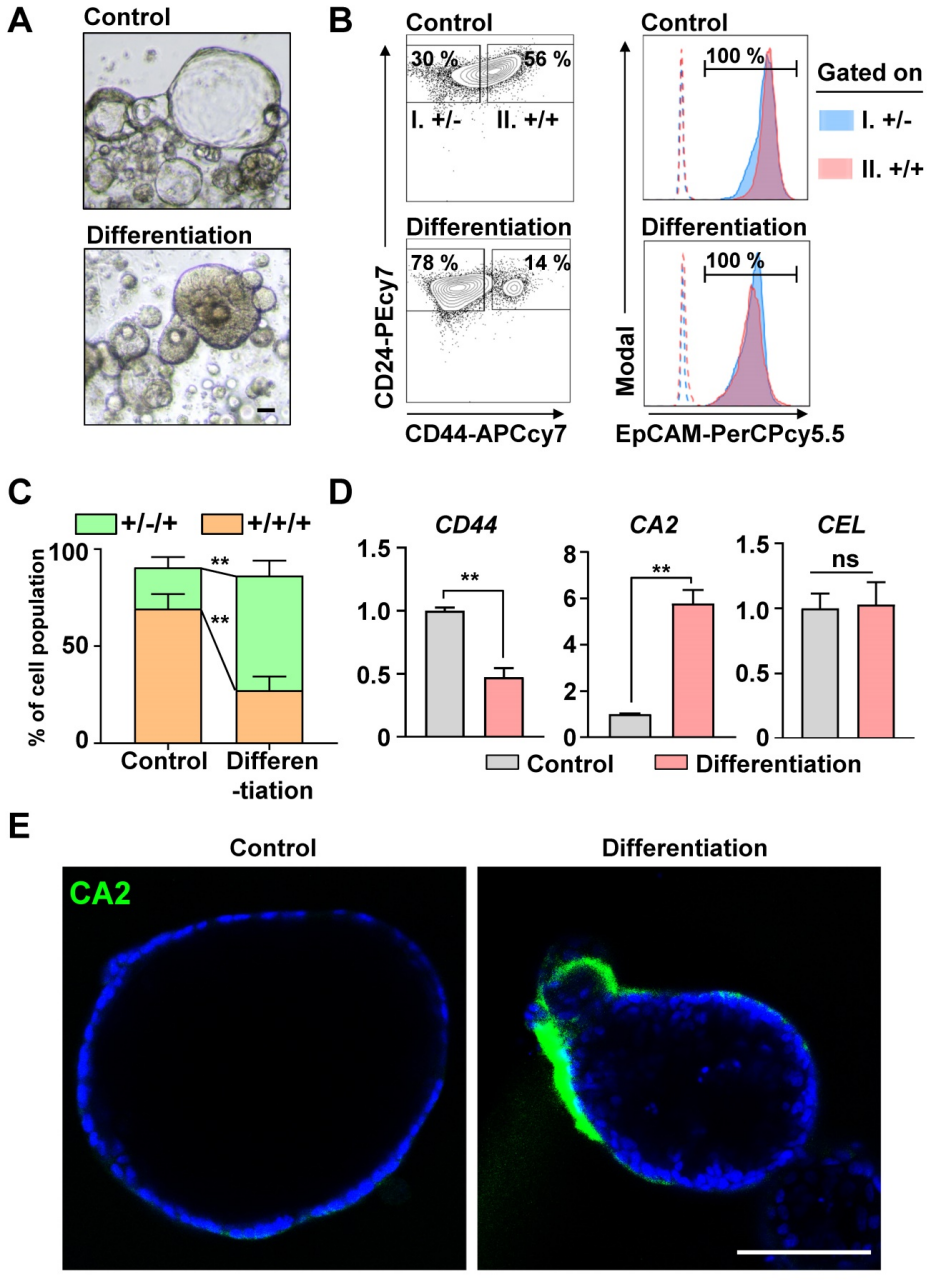
** Inhibiting the Wnt/NOTCH pathway caused CD44(+)CD24(+)EpCAM(+) cancer-initiating cells (CICs) to spontaneously differentiate into epithelial ductal carcinomas. (A)** Representative bright-field images of control and differentiated cancer organoids. Scale bar, 40 μm. **(B)** Fluorescence-activated cell sorting (FACS) plot showing the surface expression of CD24, CD44, and EpCAM on human pancreatic cancer organoids and differentiated organoids. **(Left)** Two groups: (I) CD24(+)CD44(-) and (II) CD24(+)CD44(+) cells. **(Right)** EpCAM expression levels in the two groups. Dotted lines indicate fluorescence minus one (FMO). **(C)** Quantification of the CD24(+)CD44(-)EpCAM(+) and CD24(+)CD44(+)EpCAM(+) populations in control and differentiated organoids (N = 3 biological replicates, **P < 0.05, Sidak multiple comparisons test). **(D)** *CD44*, *CA2*, and *CEL* mRNA levels (N = 3 biological replicates with at least triplicate experiments, **P < 0.05, ns = non-significant, Mann-Whitney *U* test). **(E)** Confocal image showing CA2 (green) expression in control and differentiated cancer organoids. Scale bar, 100 µm.

In our previous study 13, > 80% of the cells in human PDAC organoids were CICs, and the Wnt/NOTCH pathway played a pivotal role in maintaining them. The CICs can recapitulate the cellular heterogeneity of the original tumor 3. We hypothesized that the CICs in the organoids could be differentiated into a ductal or an acinar lineage. As a spontaneous differentiation medium, we tested the basal medium with EGF, which plays a pivotal role in the growth and differentiation of epithelial progenitor cells during pancreatic organogenesis 26-28, and added NOTCH/Wnt inhibitors to inhibit the stemness. After 2 days, the morphology of the organoids changed from cystic (clear lumen) to dense **(Figure 1A)**. Also, the number of CD24(+)CD44(+)EpCAM(+) CICs decreased significantly as those cells became CD24(+)CD44(-)EpCAM(+) cells **(Figures 1B and 1C)**. The differentiated cancer cells had significantly increased the levels of CA2 (a ductal epithelial cell marker), while the levels of CEL (an acinar cell marker) did not change **(Figures 1D and 1E)**. Therefore, the CICs in the PDAC organoids were differentiated into ductal-like cancer cells via the Wnt/NOTCH inhibition and EGF pathway.

### Differentiated ductal carcinomas were re-programmed by endothelial cells and immune cells

**Figure 2:**
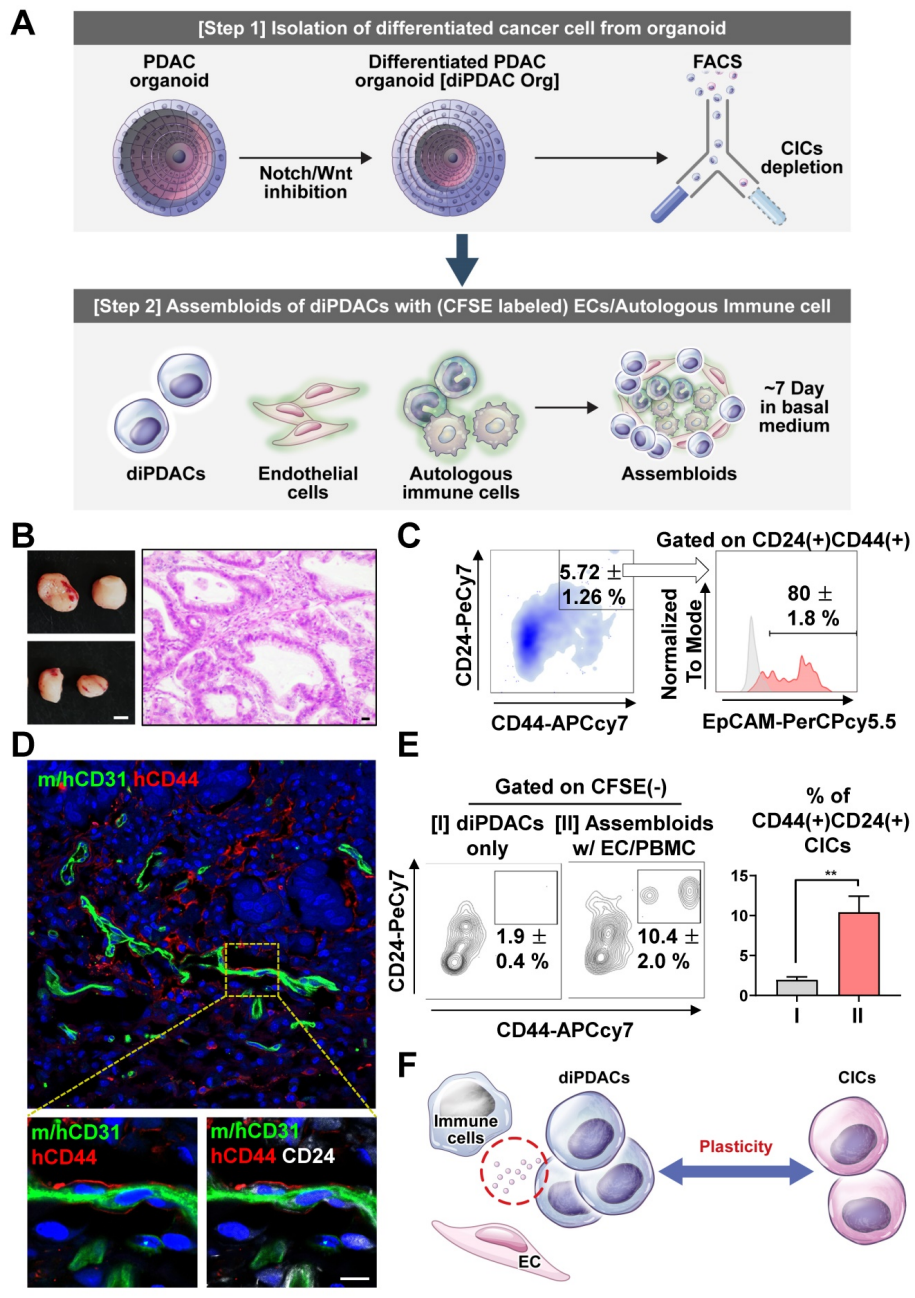
** Differentiated CD44(-) cancer cells from PDAC organoids reprogrammed as CICs by endothelial cells and immune cells. (A)** Schematic figure of the experiment. [Step 1] Isolation of differentiated cancer cells from PDAC organoids after culture in the differentiation condition. [Step 2] Establishment of assembloids with differentiated cancer cells, endothelial cells, and autologous immune cells, followed by culture for 7 days in basal medium. [Step 3] Analysis of the cancer cell plasticity capacity *in vivo* and *in vitro*. **(B-D)** Xenotransplantation of assembloids after culture in basal medium. **(B) (Left)** Tumor mass image. Scale bar, 5 mm. **(Right)** H&E staining. Scale bar 20 μm, **(C)** Representative FACS plot showing CD24(+)CD44(+) and CD24(+)CD44(+)EpCAM(+) CIC populations from tumor mass. **(D)** Frozen section image of m/hCD31 (green), hCD44 (red), and hCD24 (white). Scale bar, 10 μm. **(E) (Left)** Analysis of CIC population after 7-day culture of (I) differentiated PDAC cells (diPDACs) and (II) assembloids containing diPDACs/ECs/autologous immune cells. ECs and immune cells labeled with CFSE dye. **(Right)** Quantification of the CFSE(-)CD24(+)CD44(+) CIC population (N = 3 biological replicates, **P < 0.05, Mann-Whitney *U* test). **(F)** Schematic figure for cancer cell plasticity driven by ECs and immune cells.

To investigate the cancer plasticity capacity, we first differentiated the CICs in the PDAC organoids into ductal carcinoma (differentiated PDAC, diPDAC) using the differentiation medium. Then, we depleted the residual non-differentiated CD44(+) CICs by FACS sorting. Next, we established the assembloids of the differentiated ductal-like cancer cells, endothelial cells (ECs), and autologous immune cells. These assembloids were cultured for 7 days in the basal medium with serum. Finally, we tested the plasticity of the ductal-like cancer cells using *in vitro* and xenotransplantation models **(Figure 2A)**. To determine whether ability for cancer-initiating by plasticity in the assembloids (ductal-like cancer cells with ECs and autologous immune cells), we transplanted two assembloids into NSG-(*K^b^D^b^*)^null^ (*IA*)^null^ mice, which exhibited resistance to graft-versus-host disease for human PBMCs 29. Tumors were extracted from the mice after 6 weeks **(Figure 2B)**, and then analyzed using a FACS analysis and immunofluorescence. Interestingly, CD24(+)CD44(+)EpCAM(+) CICs from the differentiated ductal-like cancer cells were found in the tumor mass **(Figure 2C)**. Also, the CD24(+)CD44(+) CICs interacted with the ECs in the vascular microenvironment **(Figure 2D)**. To investigate the plasticity of the ECs and immune cells, we cultured the assembloids containing differentiated ductal-like cancer cells with ECs and autologous immune cells and only differentiated ductal-like cancer cells from the organoids in the basal medium with serum. We found that the assembloids of ductal-like cancer cells with ECs and autologous immune cells had significantly higher CIC populations **(Figure 2E)**. Therefore, the ductal-like cancer cell by ECs and immune cells was induced plasticity, enabling the differentiated epithelial ductal carcinomas to be reprogrammed into CICs **(Figure 2F)**.

To determine the 2^nd^ differentiation capacity by CD44(+) CICs in the organoids, we analyzed the sorted CD44(+) CICs after the differentiation process. These cells performed 2^nd^ differentiation after conformation of organoid structure **(Figure S1B)**. As a result, the sorted CD44(+) CICs in the diPDAC organoids demonstrated capacity for 2^nd^ differentiation **(Figure S1C)**. Therefore, residual CICs after differentiation step in the organoids could be could be the cause of tumor heterogeneity by asymmetric division.

### Transcriptome dynamics during re-programming were related to the Wnt/beta-catenin pathway

**Figure 3:**
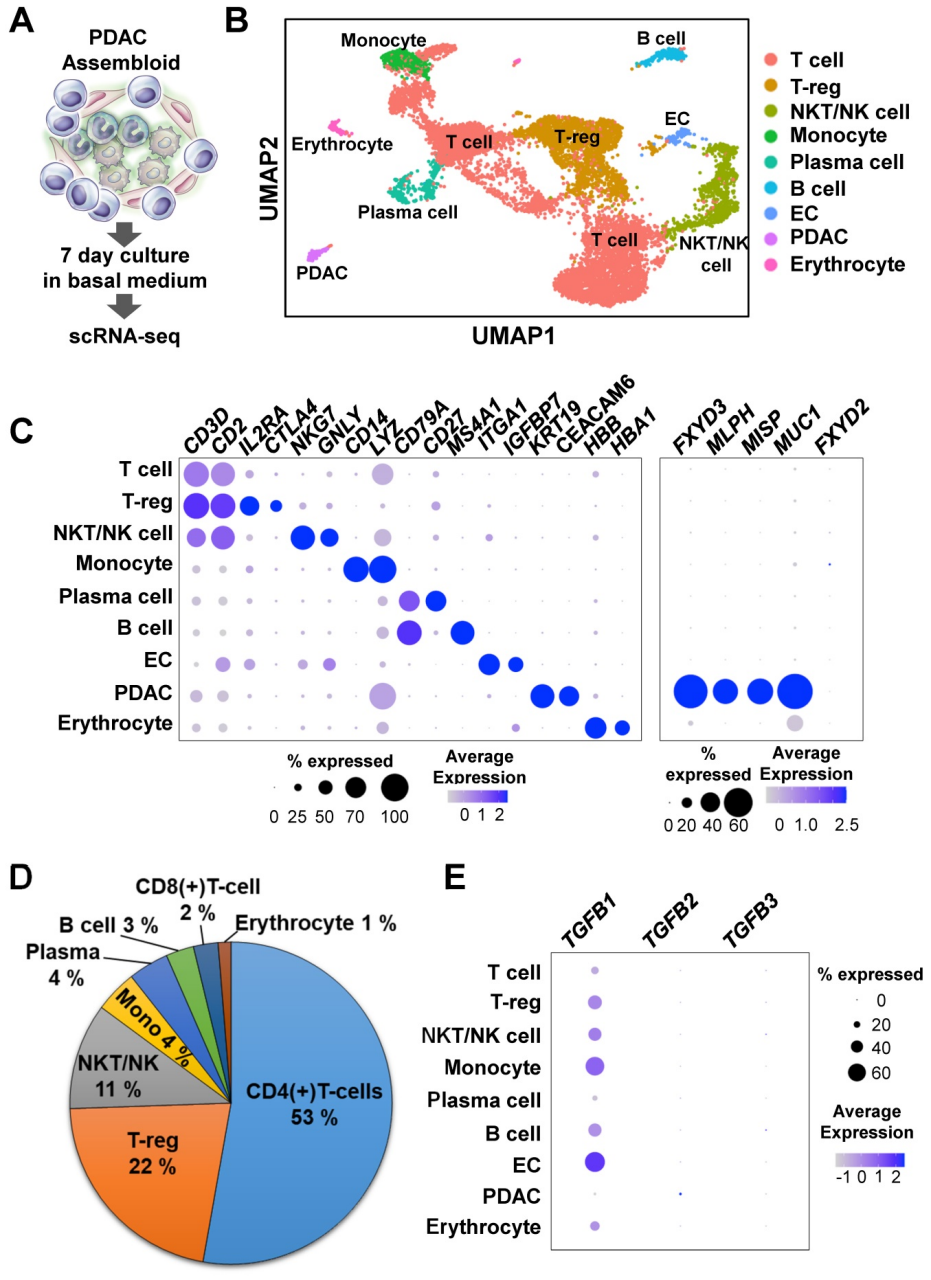
** Single-cell RNA-seq analysis of PDAC assembloids containing endothelial cells and autologous immune cells. (A)** Graphical figure of single-cell RNA-seq. **(B)** Clustering of different cell types. **(C)** Selective cell type-specific markers are shown in a bubble plot. The dot size indicates the percentage of cells that express a specific marker, and the color intensity represents the level of mean expression. **(D)** Proportion of immune cells. **(E)** Bubble plot showing the *TGFB1*, *TGFB2*, and *TGFB3* levels in different cell types.

To investigate the mechanisms of cancer plasticity provided by the ECs and immune cells, we used single-cell RNA-seq to analyze assembloids that had been cultured in the basal medium with serum for 7 days **(Figure 3A)**. The nine major clusters were identified through the Uniform Manifold Approximation and Projection (UMAP) analysis **(Figures 3B and 3C)**. To validate the cluster identify, we defined the markers using the Seurat functions “FindMarkers” and “PanglaoDB” 17 **(Figures 3B and 3C)**. To confirm expression differences between normal pancreas organoids and our samples, we identified the genes that were more highly expressed in pancreatic cancer tumors than in normal pancreatic tissue using the TCGA-PAAD data and the R package caret, a machine learning technique 25. Compared with the normal group, the tumor group had higher expressions of *MLPH*, *MISP*, *KDM6B*, and *FXYD3* genes **(Figure S2A)**. The *MLPH*, *MISP*, *KDM6B*, and *FXYD3* genes were also specifically expressed in the PDAC assembloid cluster **(Figure 3C)**. In a recent study, the *MUC1* and *FXYD3* genes were identified as specifically malignant, whereas *FXYD2* was found in the abnormal group 30. Consistently, we found that *MUC1*, but not *FXYD2*, was specifically expressed in the PDAC clusters **(Figure 3C)**.

In a previous report, the immune-mediated microenvironment in PDAC was found to contain a high percentage of CD4(+) T-cells and low percentage of tumor-infiltrating CD8(+) effector cells 31. Therefore, PDAC is currently considered as a cancer with a poor immune response 32. To identify the subgroups of CD3- and CD2-expressing T cells, we analyzed the DatabaseImmuneCellExpressionData in the R package SingleR (v1.4.1) 18. We found that a high percentage of the cell population were CD4(+) T-cells, and a much lower percentage of the cell population were CD8(+) T-cells **(Figure 3D and Figure S2B)**. The assembloids also had highly enriched T-reg and CD4(+) naïve T cells **(Figures 3B and 3D)**. TGFB1, a positive regulator for the expansion and differentiation of T-reg cells 33, was highly expressed in the ECs and monocytes **(Figure 3E)**. Therefore, we suggest that the PDAC-organoid-derived assembloids can be used as an avatar model of the corresponding patients.

**Figure 4:**
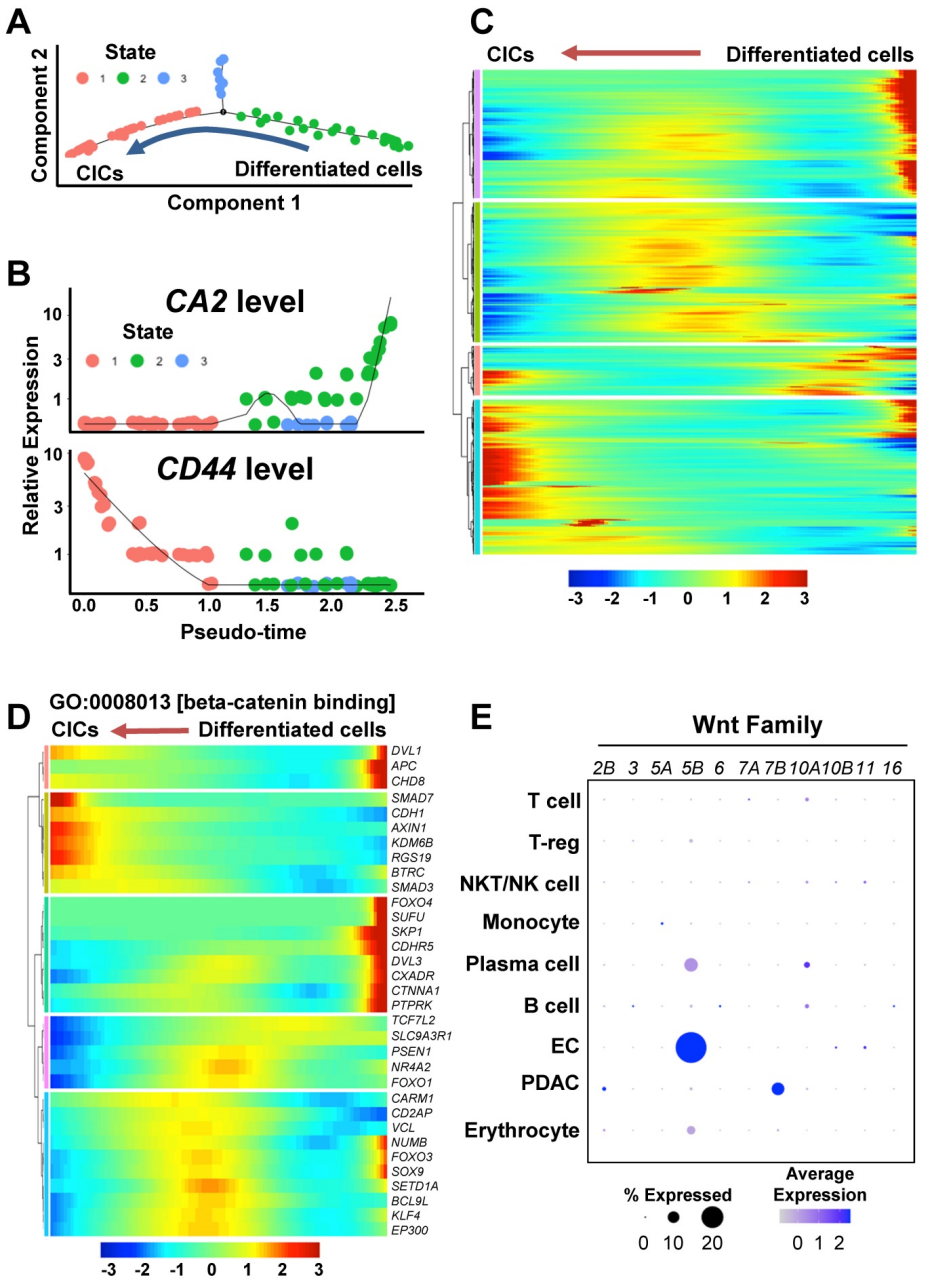
** Wnt/β-catenin pathway during the process of cancer cell plasticity using a trajectory analysis. (A)** Trajectory analysis of CICs and differentiated ductal cancer cells in the PDAC cluster in the single-cell RNA-seq dataset. **(B)** *CA2* and *CD44* levels in the CICs and differentiated ductal cancer cells in the trajectory analysis. **(C)** Heatmap of the differentially expressed genes during the reprogramming process. The gene list contains the genes that were significantly regulated in States 1, 2, and 3 (P < 0.05, T-test). **(D)** Heatmap showing GO:0008013 [β-catenin binding] in the differentially expressed gene list. **(E)** Expression levels of *WNT2B, WNT3, WNT5A, WNT6, WNT7A, WNT7B, WNT10A, WNT10B, WNT11,* and* WNT16* in the PDAC assembloids.

To investigate transcriptome dynamics during the re-programming process, we performed a trajectory analysis of the PDAC cluster. The PDAC cells were divided into three states according to CA2 and CD44 expression levels: “State 1” as CA2(-)CD44(+) CICs, “State 2” as CA2(+)CD44(-) differentiated ductal-like cancer cells, and “State 3” as CD44/CA2-negative cells **(Figures 4A and 4B)**. When we examined the genes that were differentially expressed in each state (State 1 vs. 3, State 1 vs. 2, and State 2 vs. 3), we found 4,333 genes (p < 0.05, T-test) **(Table S2)** and displayed the dynamics in expression levels during the cancer plasticity process in **Figure 4C**. To investigate the enriched gene ontology of the 4,333 genes, we performed a DAVID analysis for molecular function (MP) and biological process (BP) **(Figures S3A and S3B)**. Interestingly, β-catenin binding (GO:0008013) related genes was significantly regulated during the re-programing process **(Figure S3A)**. Specifically, *KDM6B*, *RGS19*, and *SMAD3*, which belong to beta-catenin binding in the gene ontology, were up-regulated during the process of cancer plasticity **(Figure 4D)**. Among the Wnt ligands upstream of the β-catenin pathway,* WNT5B* was highly expressed in the ECs of the PDAC assembloids **(Figure 4E)**. The BP analysis revealed that apoptotic process (GO:0006915) genes were highly enriched **(Figure S3B)**. The three distinctively classified states had different gene expression patterns for the apoptotic process **(Figure S3C)**. Therefore, the diversity of apoptosis machinery could be the cause of drug resistance and tumor heterogeneity by the means of cancer cell plasticity.

### Molecular subtype of PDAC in organoids and assembloids

The major molecular subtype of PDAC consistently were defined as classical epithelial, quasi-mesenchymal (QM), and exocrine-like subtypes 34. Especially, the QM subtype (akin to hybrid epithelial/mesenchymal), which co-expresses certain epithelial and mesenchymal genes, induced drug resistance and poor survival 34-37. We performed analysis to identify the epithelial CICs and QM CIC states in the organoids and assembloids. Major components of PDAC organoid were CD24(+)CD44(+)CDH1(+)CDH2(-) epithelial CICs **(Figure S3D)**. Meanwhile, CD24(+)CD44(+)CDH1(+)CDH2(+) QM CICs were identified rarely (< 1%) in the organoids **(Figure S3D)**. Also, the PDAC assembloids mainly had *CDH1*/*EPCAM*-expressing epithelial subset in the CD44(+) CIC population (State 1 in the trajectory analysis) **(Figures S3E and S3F),** and rarely included *Vimentin* and *ZEB1*-expressing cells as a biomarker of QM CICs** (Figure S3G)**. Next, we analyzed the "PDAssigner” signature 34 as another QM biomarker in the PDA in the assembloids. We identified QM-PDA cells subtype, which is expressed *HK2*, *PHLDA1*, *PMAIP1* and *SLC5A3,* in the CIC population **(Figure S3H).** Taken together, the CICs in the organoids and assembloids were mainly in the composite epithelial state and rarely in the QM state.

### *WNT5B* and *TGFB1* were up-regulated by means of the NOTCH/JAG1 pathway in ECs

**Figure 5:**
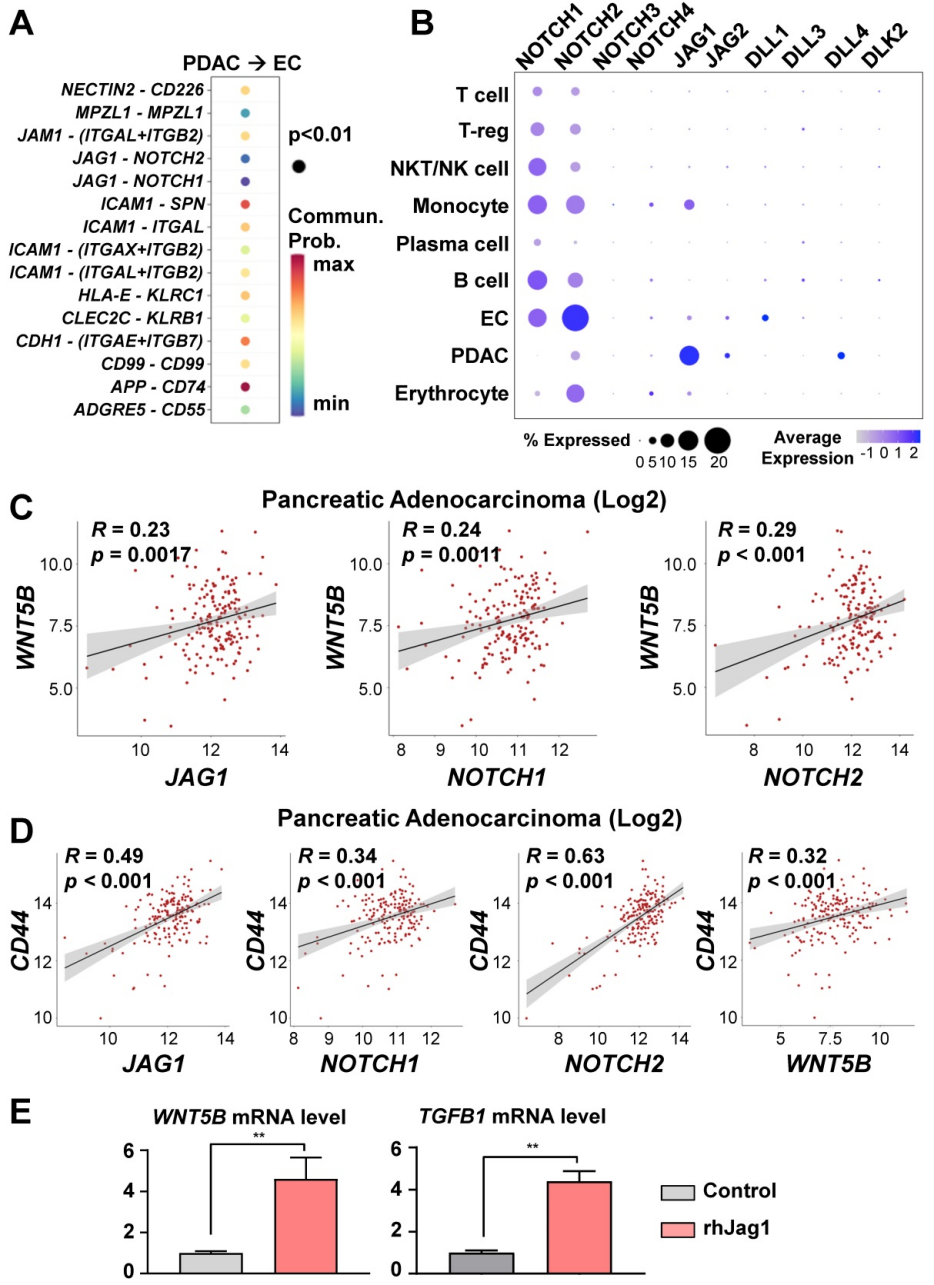
***WNT5B* and *TGFB1* levels regulated via the JAG1/NOTCH pathway. (A)** Significant ligand-receptor pairs from PDAC and ECs in the single-cell RNAseq. **(B)** Bubble plot showing the levels of the NOTCH receptor family (*NOTCH1, NOTCH2*,* NOTCH3*, and* NOTCH4*) and ligands (*JAG1*,* JAG2*, *DLL1*,* DLL3*,* DLL4*, and* DLK2*) in different cell types. **(C)** Pearson correlation analysis for *WNT5B* with *JAG1*, *NOTCH1,* and *NOTCH2* mRNA levels in human pancreatic adenocarcinoma (N = 178) from TCGA data. **(D)** Pearson correlation analysis for a CIC biomarker (*CD44*) with *JAG1*, *NOTCH1*, *NOTCH2*, and *WNT5B* mRNA levels in human pancreatic adenocarcinoma (N = 178) from TCGA data. **(E)** Quantification of *WNT5B* and *TGFB1* mRNA levels with rhJAG1 in ECs (**P < 0.05, Mann-Whitney *U* test).

To investigate the cell-to-cell interaction pathway between PDACs and ECs, we analyzed significant cell-cell interaction pathway using CellChat. As a result, we identified the NOTCH, ICAM1, and CDH1 pathways, which are associated with stemness and metastasis **(Figure 5A)**. In our previous study 13, we reported that endothelial protein secreted by the NOTCH pathway maintains CICs in PDAC organoids. Therefore, we hypothesized that NOTCH pathway activation in ECs by JAG1 on cancer cells would induce *WNT5B* expression. *NOTCH2* and *JAG1* are among the NOTCH ligand and receptor family genes expressed on the ECs and PDACs of the assembloids, respectively **(Figure 5B)**. To determine potential correlations among the *WNT5B*, *JAG1*, *NOTCH1*, *NOTCH2*, and *CD44* levels, we analyzed the TGCA-PAAD dataset. *WNT5B* correlated positively with the *JAG1*, *NOTCH1*, and *NOTCH2* levels **(Figure 5C)**. Also, the expression level of *CD44*, a CIC marker, correlated positively with those of *JAG1*, *NOTCH1*, *NOTCH2*, and *WNT5B* **(Figure 5D)**. To validate the regulation of WNT5B expression through JAG1, we seeded ECs in a plate coated with recombinant JAG1 **(Figure 5E)** and found that *WNT5B* in the ECs was significantly up-regulated by JAG1. TGFB1, an inducer of T-reg expansion and differentiation, was also up-regulated by JAG1 **(Figure 5E)**. Moreover, the inhibition NOTCH pathway by DAPT treatment reversed the increased WNT5B and TGFB1 levels by rhJAG1 **(Figure S4A).** Therefore, NOTCH and JAG1 interaction induce upregulation of WNT5B and TGFB1 level in the ECs.

### JAG1 plays pivotal role in cancer plasticity

**Figure 6:**
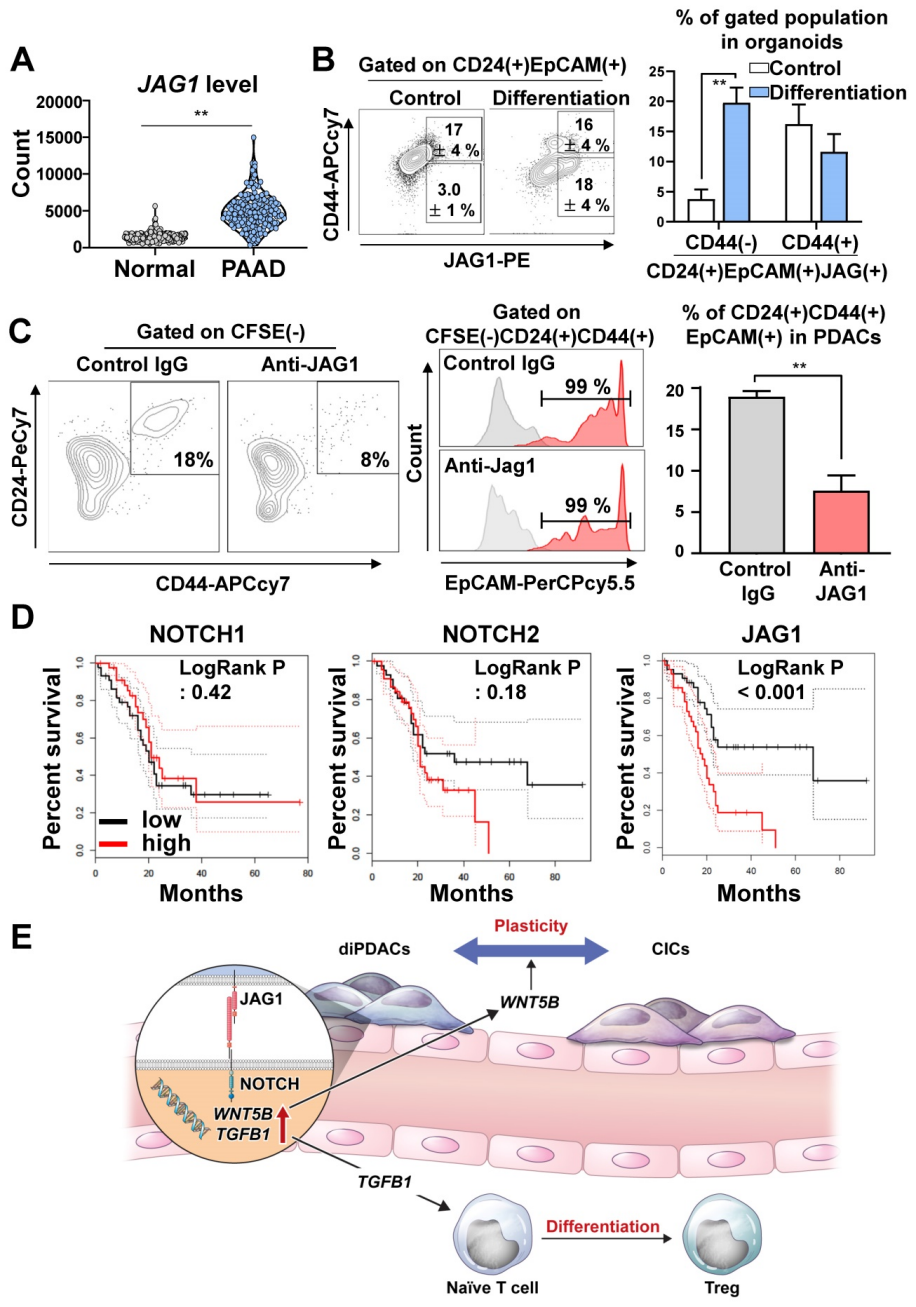
** JAG1 on cancer cells plays important role in plasticity. (A)** Analysis of the *JAG1* mRNA level in the normal pancreas samples from the GTEx dataset and the PAAD TCGA dataset using recount3 (**P < 0.05, T-test). **(B) (Left)** FACS plot showing an analysis of the CD24(+)CD44(-)EpCAM(+)JAG1(+) and CD24(+)CD44(+)EpCAM(+)JAG1(+) populations in human pancreatic cancer organoids and differentiated organoids. **(Right)** Quantification of the CD24(+)CD44(-)EpCAM(+)JAG1(+) and CD24(+)CD44(+)EpCAM(+)JAG1(+) populations in the indicated groups (N = 3 biological replicates, **P < 0.05, Bonferroni's multiple comparisons test). **(C) (Left)** FACS plot showing CFSE(-)CD24(+)CD44(+)EpCAM(+) CICs after co-culture of PDAC organoid derived CD44(-) differentiated cancer cells and CFSE-labeled HUVECs for treatment with control IgG and JAG1-neutralizing antibody. **(Right)** Quantification of the CFSE(-)CD24(+)CD44(+) CICs in the indicated groups (N = 3 biological replicates, **P < 0.05, Mann-Whitney *U* test). **(D)** Overall survival analysis according to NOTCH1, NOTCH2, and JAG1 expression levels using TCGA data. **(E)** Graphical summary of the study, suggesting pivotal role of JAG1 for PDAC plasticity.

To determine how the JAG1 expression level in pancreatic cancer cells was comparatively different from normal pancreatic tissues, we analyzed TCGA-PAAD and the normal pancreas in the GTEx dataset, and found that JAG1 was significantly over-expressed in human pancreatic cancer compared to the normal pancreas **(Figure 6A)**. Next, we analyzed the JAG1 level in the organoids during differentiation. Interestingly, the JAG1(+)CD24(+)CD44(-)EpCAM(+) non-CIC population in the PDAC organoids was increased by differentiation **(Figure 6B)**.

To determine whether cancer plasticity was affected by the NOTCH/JAG1 pathway, we cultured the organoid-derived differentiated CD44(-) cancer cells with ECs after neutralizing JAG1. We found that plasticity, which re-programmed CD44(-) differentiated cancer into CD24(+)CD44(+)EpCAM(+) CICs, was inhibited when JAG1 was neutralized **(Figure 6C)**. Similarly, the plasticity of cancer cell in the assembloids was prevented by the inhibition of NOTCH pathway through DAPT treatment **(Figure S5A)**. Consistently, the clinical data analysis using TCGA-PAAD indicated that high JAG1 expression correlated with poor survival, while high expressions of *NOTCH1, NOTCH2, NOTCH3,* and *DLL4* did not affect survival **(Figure 6D and Figure S5B).** These results suggest that the NOTCH2/JAG1 pathway between EC and PDAC plays an important role in the plasticity of cancer cells, and NOTCH2/JAG1 pathway could have caused poor survival by tumor heterogeneity.

## Discussion

Resistance mechanisms often develop after continuous exposure to chemotherapy and targeted therapy regimes 38. Along with well-known genetic changes, cell plasticity has recently emerged as a major factor in therapy resistance 38. In the present study, we used PDAC organoids to demonstrate a mechanism for dynamic changes in CICs and ductal cancer cells. CICs were differentiated into ductal cancer cells by inhibiting the Wnt/NOTCH pathway. To investigate the plasticity of differentiated ductal cancer cells in a tumor microenvironment, we established PDAC assembloids that contained ECs and autologous immune cells. Using those PDAC assembloids, we demonstrated that ECs induced plasticity in differentiated ductal cancer cells. Furthermore, stimulating ECs with JAG1 expressed on cancer cells could increase the expansion and differentiation of T-reg by up-regulating TGFB1. WNT5B and TGFB1 expressions were increased in ECs through the interaction with JAG1 on cancer cells. Moreover, neutralizing JAG1 inhibited cancer cell plasticity, which otherwise produced drug resistance by maintaining cancer heterogeneity **(Figure 6E)**.

In this study, we demonstrated that the predominant cell population in the PDAC organoids and assembloids was the epithelial subset, which is a specific property of cancer-initiating cells (CICs), and that the QM CIC subtype was rarely found. The QM states of cancer cells are partially mesenchymal cells, which are capable of transitioning to an epithelial state 36,37. Moreover, QM cancer cells are reported to induce drug-resistance and poor survival 34,35. Currently, QM CICs, which are expected to induce stemness and tumor heterogeneity, are not yet a well-defined population. Also, the precise mechanism of dynamic switch between epithelial status and mesenchymal status in the QM CICs has remained unanswered. The use of PDAC organoids and assembloids could be a good experimental model for the investigation on expansion, differentiation, and drug response of QM CICs. Further studies are needed to explore the role of tumor heterogeneity and mechanism for dynamic transition of QM CICs using PDAC assembloids.

The NOTCH pathway plays an important role as a progenitor of cell differentiation during the development of the pancreas 39,40, and the NOTCH level is up-regulated in PanIN 41,42, a premalignant lesion 43. The *NOTCH1* gain-of-function in the Kras-induced mouse model increased PanIN formation 44. Also, the depletion of *NOTCH2* in the PDAC mouse model prolonged survival and decreased PanIN progression through the Myc pathway 12. As ligands of the NOTCH receptor, JAG1 and DLL4 levels were analyzed in the PDAC tumor tissues. JAG1 expression in the PDAC was significantly higher compared to those in normal pancreatic tissues, benign pancreatic tissues, and peritumoral tissues 45. Additionally, both the JAG1 and DLL4 levels were associated with poor differentiation, invasion, regional lymph node metastasis, a maximum tumor size > 5 cm, TNM III/IV disease, and survival 45. Nonetheless, the role played by JAG1 in the stemness and cancer cell plasticity of PDAC remains poorly understood. In this study, we found that the JAG1 level on PDAC organoids was increased by ductal differentiation, and JAG1 consequently increased the WNT5B and TGFb1 levels by stimulating ECs, leading to cancer cell plasticity and anti-inflammatory microenvironment.

Original CIC model (unidirectional hierarchy) and CIC plasticity model are proposed to explain for the tumor heterogeneity 46,47. Original CIC model (unidirectional hierarchy) refers to the development of the tumor heterogeneity through symmetric division (self-renewal) or asymmetric division. In the CIC plasticity model, cancer cells are able to bidirectional conversion between CIC state and non-CIC state. CIC plasticity may be caused by tumor microenvironment, which is extrinsic cues, as well as intrinsic cues 7. In this study, we demonstrated that cancer cells in the organoids and assembloids could be explained by both unidirectional hierarchy and bidirectional conversion through the NOTCH and Wnt pathway. It will be of interest to determine how cancer cells are regulated between unidirectional hierarchy and bidirectional conversion, which commonly contribute to tumor heterogeneity.

CICs are highly resistant to chemotherapy and radiation therapy. Therefore, previous publications reported the clinical feasibility of CIC-targeting therapies that inhibited the NOTCH, Hedgehog, and Wnt pathways 48. In previous studies 49,50, inhibiting NOTCH by using small molecules and antibodies to target CICs demonstrated anti-tumor activity *in vitro* and in a xenotransplantation model. However, in clinical trials, most NOTCH inhibitors were discontinued due to their limited efficacy and high toxicity. For example, combined therapy with demcizumab, an anti-humanized Delta‐like ligand 4 (anti-DLL4) IgG2, gemcitabine, and nab-paclitaxel did not show improved efficacy in metastatic PDAC patients 51,52. In a phase Ib/II trial, tarextumab, a human IgG2 antibody targeting NOTCH2 and NOTCH3, did not improve the objective response rate or OS in patients with metastatic PDAC 53. In a phase I trial, MK-0752, a gamma-secretase inhibitor, combined with gemcitabine treatment showed efficacious outcomes in 47% of stable disease and 5% of partial response patients with unresectable PDAC 54,55. However, MK-0752 was discontinued, presumably due to frequent toxicity (hypokalemia, fatigue, and anemia) and limited efficacy 55. Overall, DLL4, NOTCH2, NOTCH3, and gamma-secretase all failed as targeted therapies for PDAC. Also, the pancreatic cancer dataset in TCGA indicates that *DLL4, NOTCH1, NOTCH2*, and *NOTCH3* levels do not affect survival **(Figure 6D and Figure S5B)**. However, high JAG1 expression correlates with significantly poor survival among pancreatic cancer patients **(Figure 6D)**. As another candidate inhibitor of the NOTCH pathway, we suggest that combining conventional therapy with JAG1 neutralization to inhibit CICs and cancer cell plasticity might offer a synergic effect.

In conclusion, we replicated the cancer cells' dynamic ability to switch between a non-CIC state to a CIC state using patient sample-derived assembloids, in line with previous studies, to indicate that therapies targeting CICs have limited efficacy 56. Based on the findings that a JAG1-neutralizing antibody blocked the property of cancer cell plasticity, we suggest a humanized JAG1-neutralizing antibody as a potentially new therapeutic for PDAC.

## Supplementary Material

Supplementary figures and tables.

## Abbreviations

PDAC: pancreatic ductal adenocarcinoma
CICs: cancer-initiating cells
HUVECs: human umbilical vein endothelial cells
