# Supplementary material for: The role of Jagged1 as a dynamic switch of cancer cell plasticity in PDAC assembloids
Source: Theranostics. 2022 May 24;12(9):4431–45. doi: 10.7150/thno.71364 (PMC9169352; doi:10.7150/thno.71364)
Supplement: Supplementary file 1 — Supplementary figures and tables. [file thnov12p4431s1.pdf]

Supplementary Materials for

The role of Jagged1 as a dynamic switch of cancer cell plasticity in PDAC assembloids

Jae-Il Choi[1*], John Hoon Rim[1*], Sung Ill Jang[2], Joon Seong Park[3], Hak Park[1], Jae Hee Cho[2#] and Jong-Baeck Lim[1#]

[1]Department of Laboratory Medicine, Severance Hospital, Yonsei University College of Medicine, Seoul, Republic of Korea

[2]Institute of Gastroenterology, Department of Internal Medicine, Gangnam Severance Hospital, Yonsei University College of Medicine, Seoul, Republic of Korea

[3]Pancreatobiliary Cancer Clinic, Department of Surgery, Gangnam Severance Hospital, Yonsei University College of Medicine, Seoul, Republic of Korea

* These authors contributed equally to this work.

#Co-corresponding author: Jong-Baeck Lim, 50-1 Yonsei-Ro, Seodaemun-Gu, Severance Hospital, Yonsei University College of Medicine, Seoul, Republic of Korea

Tel: 82-2-2228-2448; Fax: 82-2-2019-8926; E-mail: jlim@yuhs.ac, jlim@yonsei.ac.kr

#Co-corresponding author: Jae Hee Cho, 211 Eonju-Ro, Gangnam-Gu, Gangnam Severance Hospital, Yonsei University College of Medicine, Seoul, Republic of Korea

Tel: +82-2-2019-3310, E-mail: jhcho9328@yuhs.ac

SUPPLEMENTARY FIGURE LEGENDS

Figure S1. (A) Sorting purity of CD44(+)PI(-) in the PDAC organoids. **(B)** Schematic figure for the experiment of 2nd differentiation capacity in the organoids. **(C)** FACS plot showing the CD24, CD44, and EpCAM on control organoids and 2nd differentiated organoids from sorted CD44(+) cells in the 1st differentiated organoid. EpCAM expression levels in the CD44(+)CD24(+) groups. Dotted lines indicate fluorescence minus one (FMO).

Figure S2. (A) Heatmap analysis of genes highly expressed in pancreatic cancer tumors compared to normal tissue in TCGA-PAAD data using the R package caret. Blue color in the row side indicates the top 100 differentially expressed genes in the normal and tumor samples. **(B)** Heatmap showing the subgroups of CD3D- and CD2-expressing clusters using the DatabaseImmuneCellExpressionData in SingleR.

Figure S3. (A) Molecular function (MP) and **(B)** biological process (BP) DAVID analysis of the significantly regulated gene list in States 1, 2, and 3 (P <0.05, T-test). **(C)** Heatmap showing the apoptotic process in genes significantly regulated in States 1, 2, and 3. **(D)** FACS plot showing the CDH1 and CDH2 in the CD24(+)CD44(+) CICs (N = 3 biological replicates). **(E-H)** Expression of markers associated with differentiated ductal, CICs **(E)**, epithelial **(F),** mesenchymal **(G),** and QM-PDA **(H)** in the CIC subset of PDAC assembloids (State 1 in the trajectory analysis).

Figure S4. (A) Quantification of *WNT5B* and *TGFB1* mRNA levels by rhJAG1 with DAPT in ECs (**P < 0.05, Bonferroni's multiple comparisons test).

Figure S5. (A) (Left) FACS plot showing CFSE(-)CD24(+)CD44(+)EpCAM(+) CICs after co-culture of PDAC organoid derived CD44(-) differentiated cancer cells, CFSE-

labeled HUVECs and autologous PBMC for treatment with control and DAPT. **(Right)** Quantification of the CFSE(-)CD24(+)CD44(+) CICs in the indicated groups (N = 3 biological replicates, **P < 0.05, Mann-Whitney *U* test). **(B)** Overall survival analysis according to *NOTCH3* and *DLL4* expression level in TCGA-PAAD data.

SUPPLEMENTARY TABLE LEGENDS

Table S1. Donor information list.

Table S2. Differentially expressed gene list according to differentiation states by trajectory analysis.

Table S3. Primer sequence information.

Figure S1

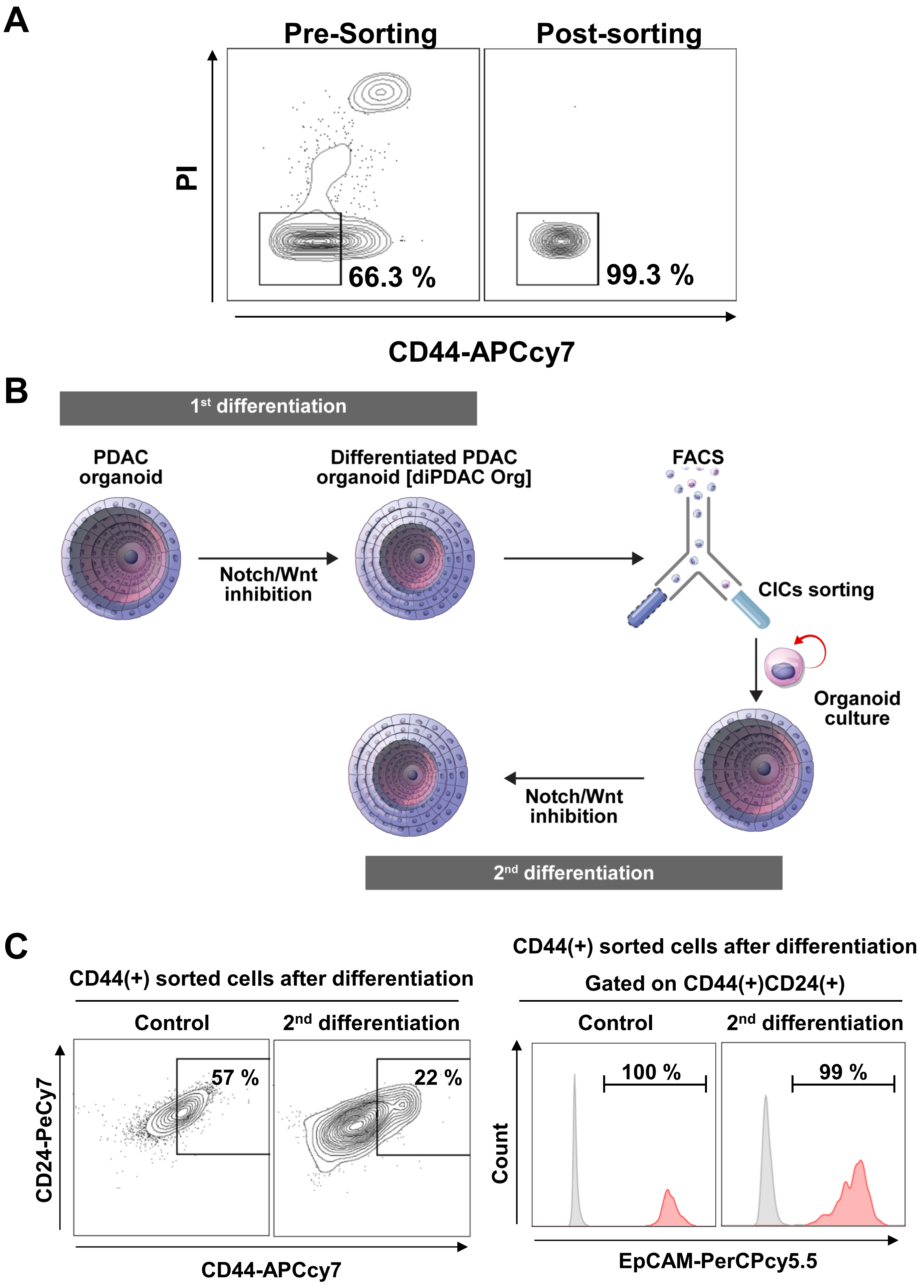
A
Pre-Sorting
Post-sorting
PI
66.3 %
99.3 %
CD44-APCcy7
B
1st differentiation
PDAC organoid
Notch/Wnt inhibition
Differentiated PDAC organoid [diPDAC Org]
FACS
CICs sorting
Organoid culture
Notch/Wnt inhibition
2nd differentiation
C
CD44(+) sorted cells after differentiation
Control
2nd differentiation
57 %
22 %
CD24-PeCy7
CD44-APCcy7
CD44(+) sorted cells after differentiation
Gated on CD44(+)CD24(+)
Control
2nd differentiation
100 %
99 %
Count
EpCAM-PerCPcy5.5

Figure S2

A

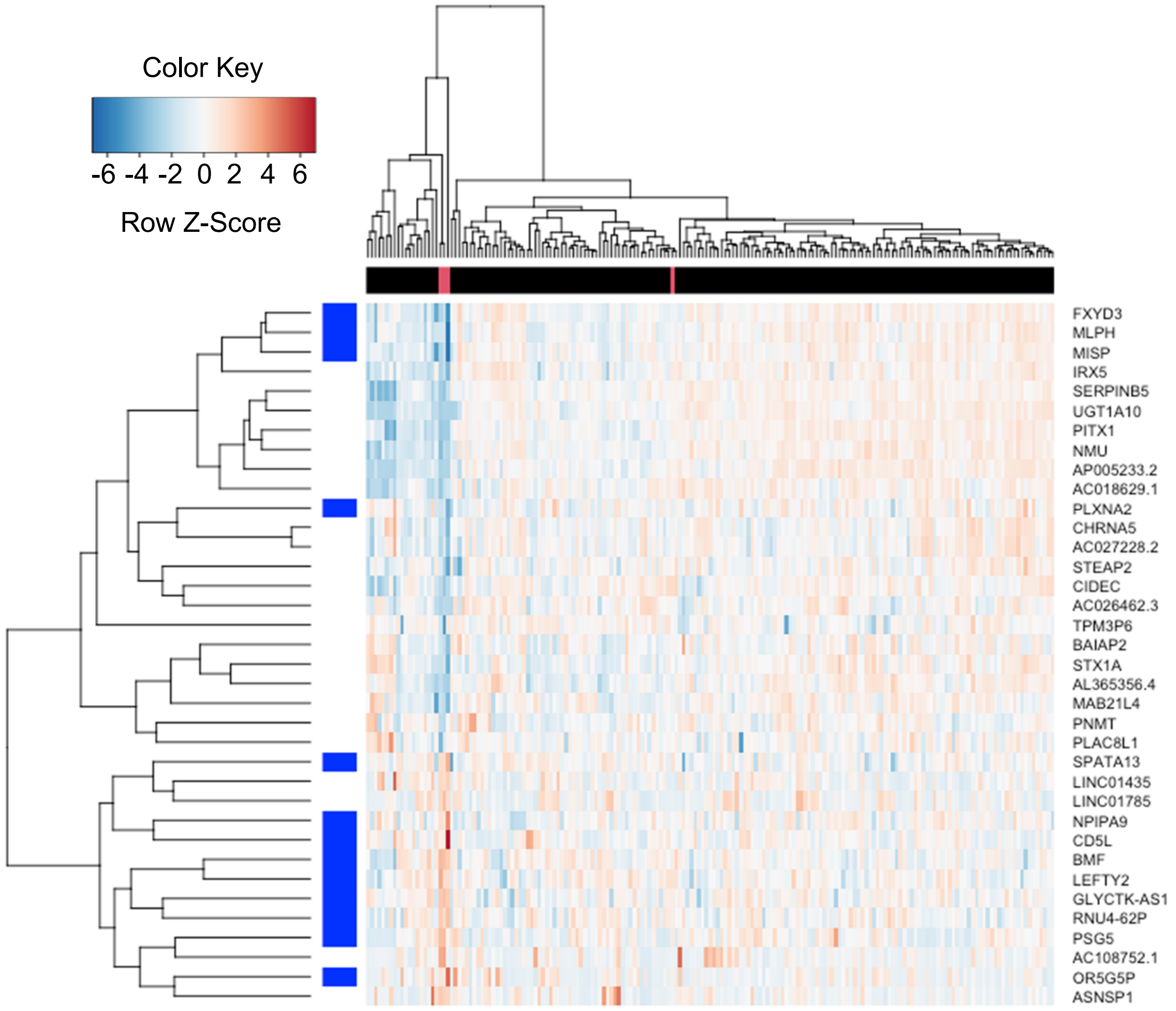

Color Key
-6 -4 -2 0 2 4 6
Row Z-Score
FXYD3
MLPH
MISP
IRX5
SERPINB5
UGT1A10
PITX1
NMU
AP005233.2
AC018629.1
PLXNA2
CHRNA5
AC027228.2
STEAP2
CIDEC
AC026462.3
TPM3P6
BAIAP2
STX1A
AL365356.4
MAB21L4
PNMT
PLAC8L1
SPATA13
LINC01435
LINC01785
NPIPA9
CD5L
BMF
LEFTY2
GLYCTK-AS1
RNU4-62P
PSG5
AC108752.1
OR5G5P
ASNSP1

B

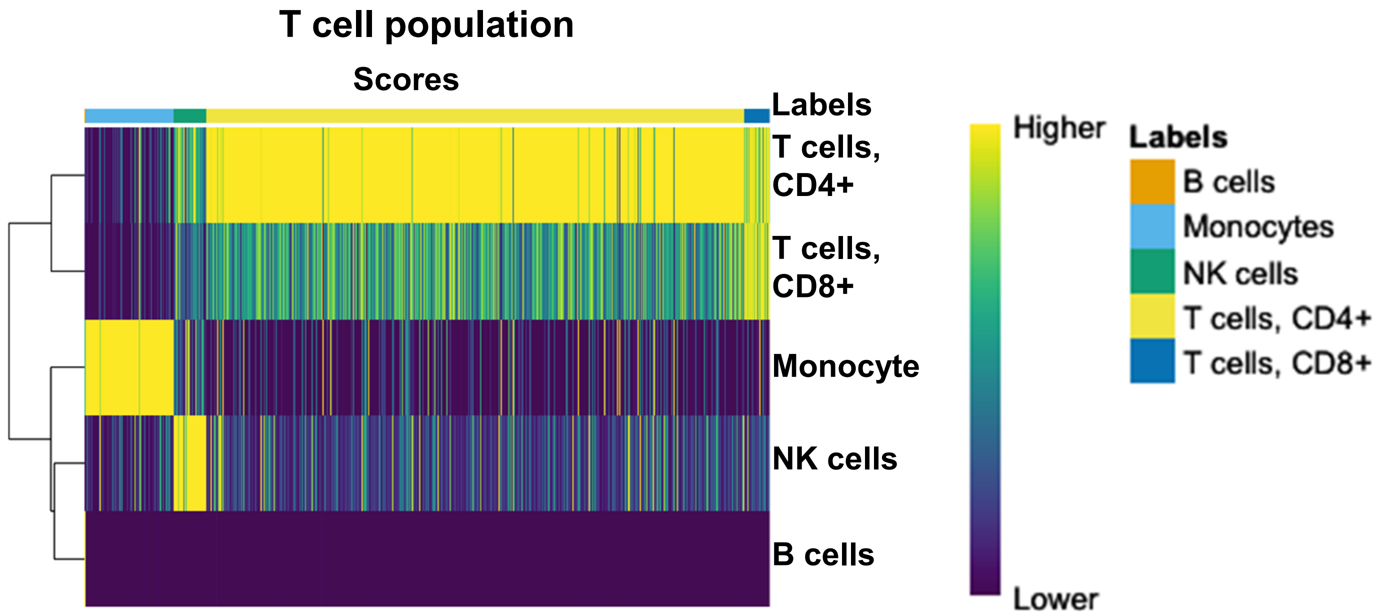

T cell population
Scores
Labels
T cells, CD4+
T cells, CD8+
Monocyte
NK cells
B cells
Higher
Lower
Labels
B cells
Monocytes
NK cells
T cells, CD4+
T cells, CD8+

Figure S3

A

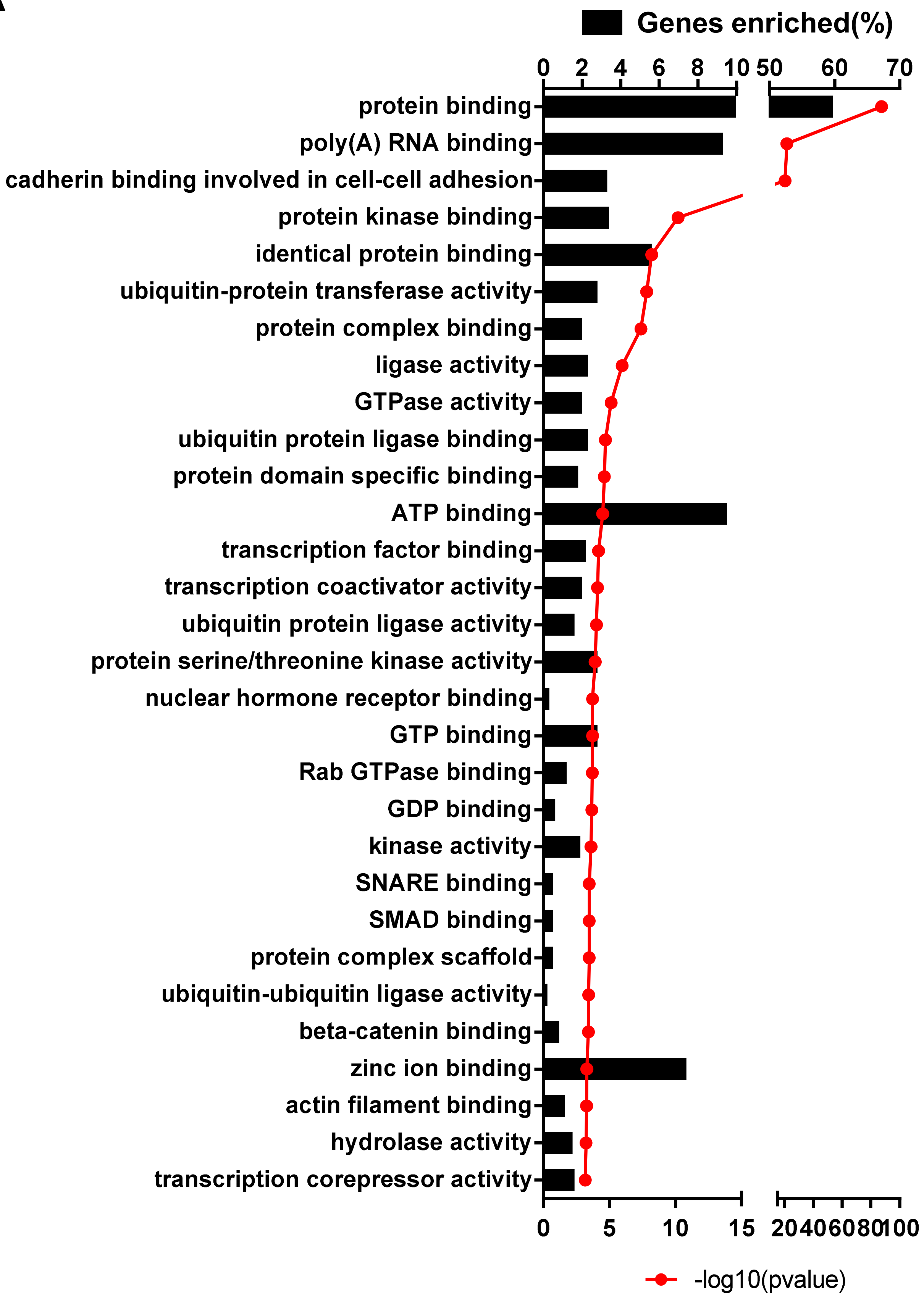

Molecular function (MP)

Figure S3

B

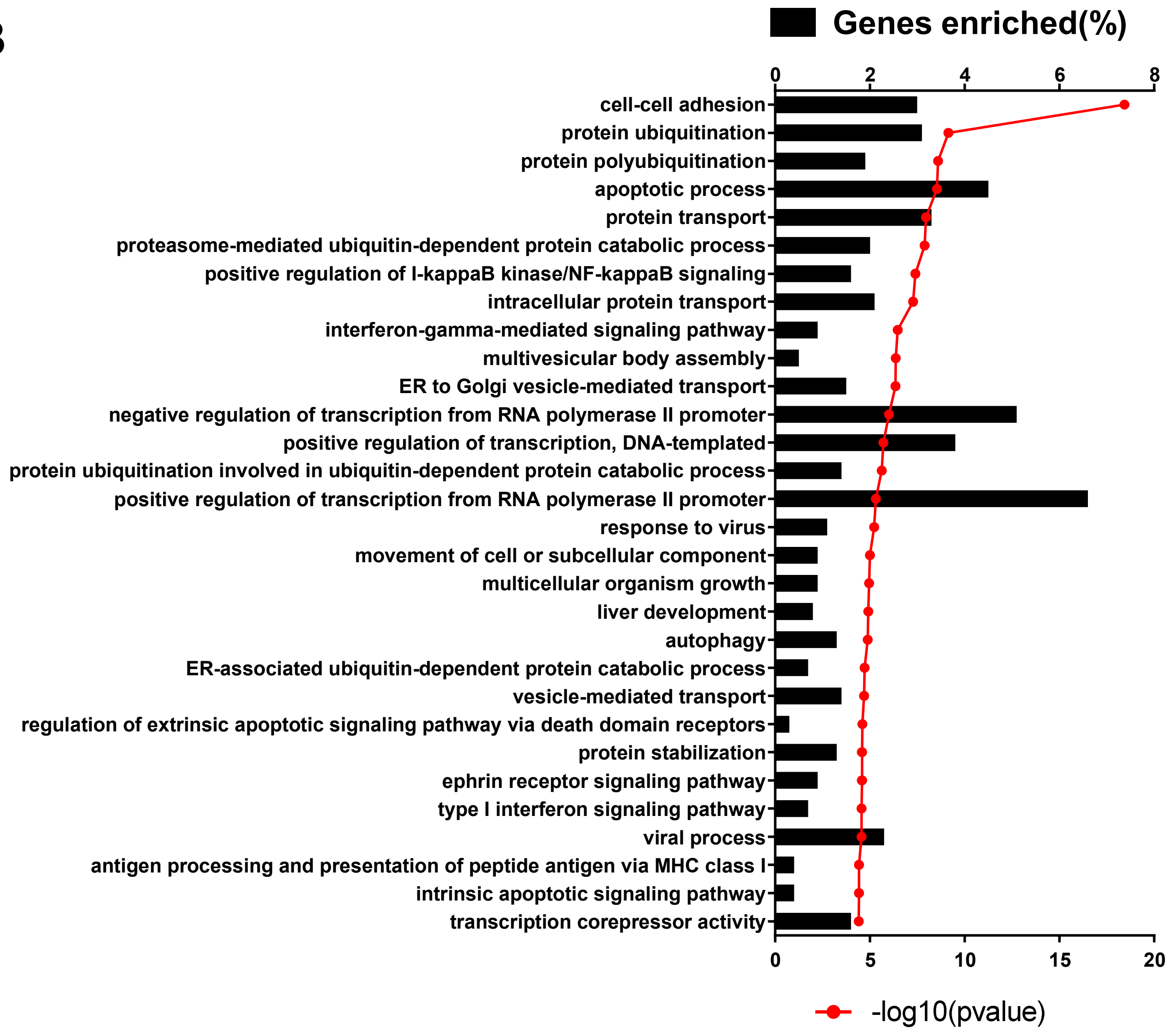

Biological process (BP)

Figure S3

C

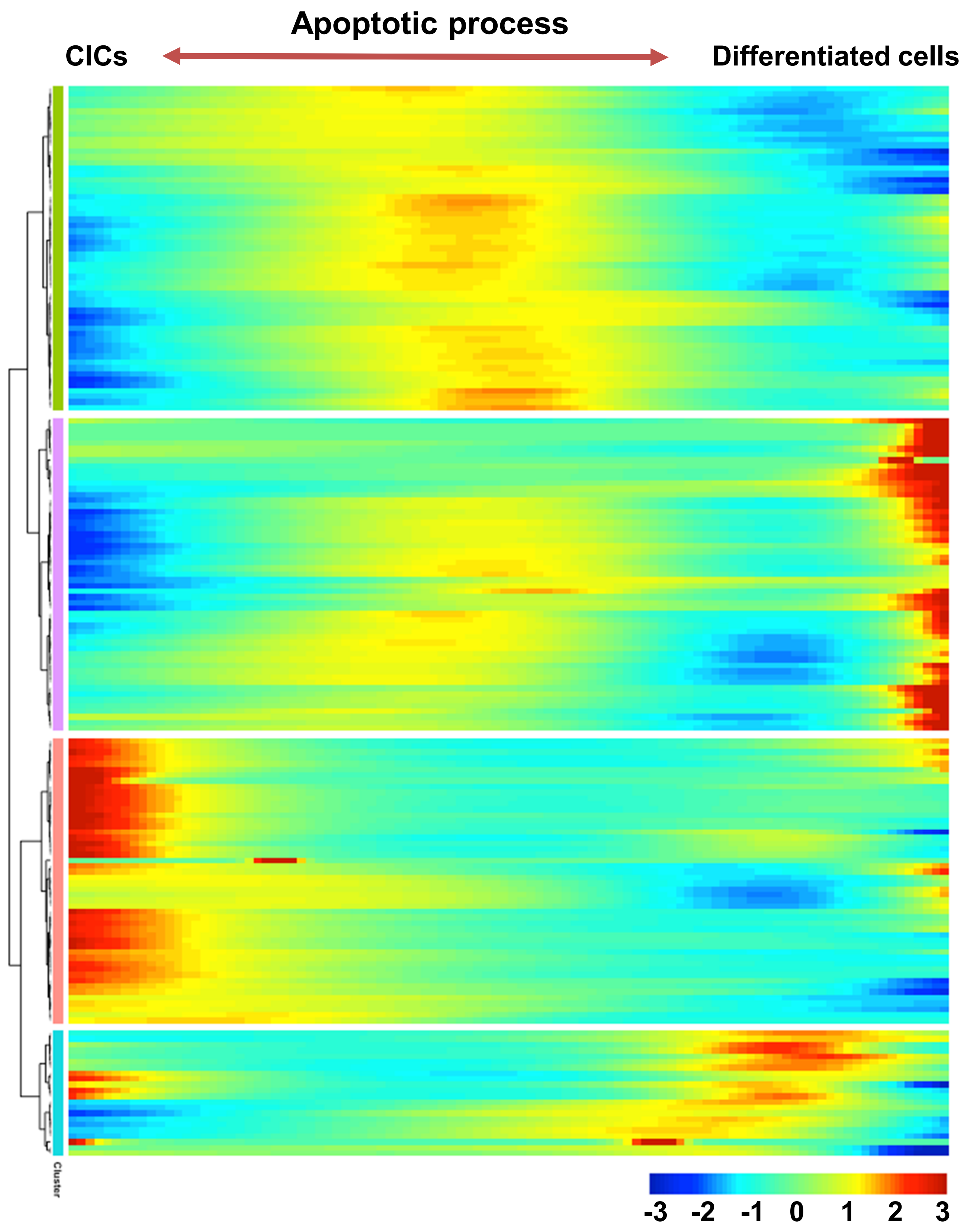

Figure S3

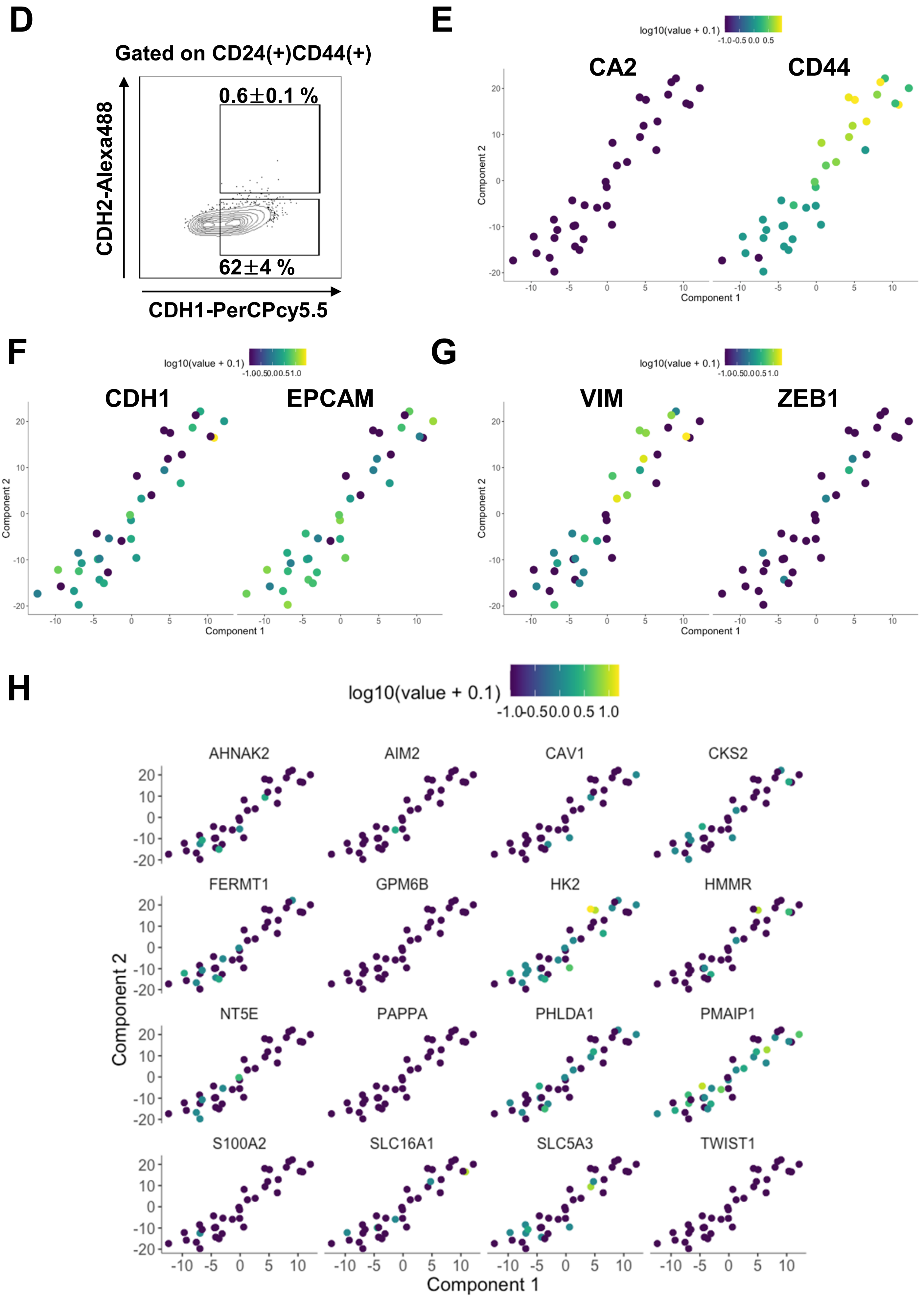

D
Gated on CD24(+)CD44(+)
0.6±0.1 %
62±4 %
CDH2-Alexa488
CDH1-PerCPcy5.5
E
log10(value + 0.1)
CA2
CD44
Component 2
Component 1
F
CDH1
EPCAM
G
VIM
ZEB1
H
AHNAK2
AIM2
CAV1
CKS2
FERMT1
GPM6B
HK2
HMMR
NT5E
PAPPA
PHLDA1
PMAIP1
S100A2
SLC16A1
SLC5A3
TWIST1

Figure S4

A

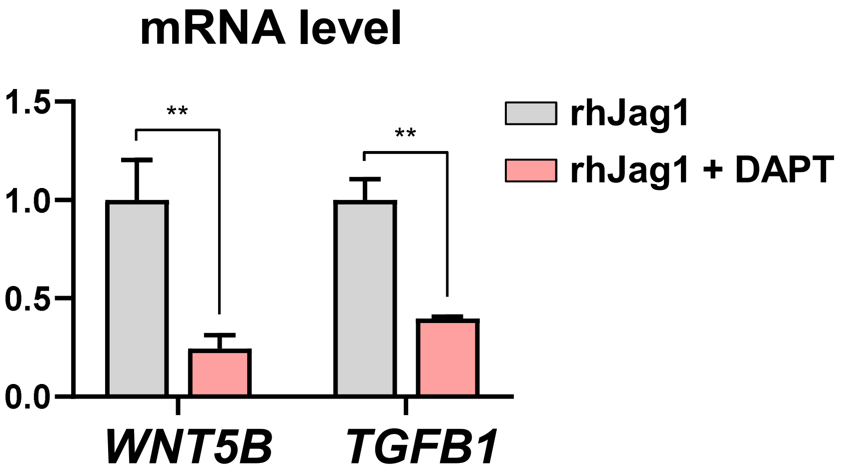

Figure S5

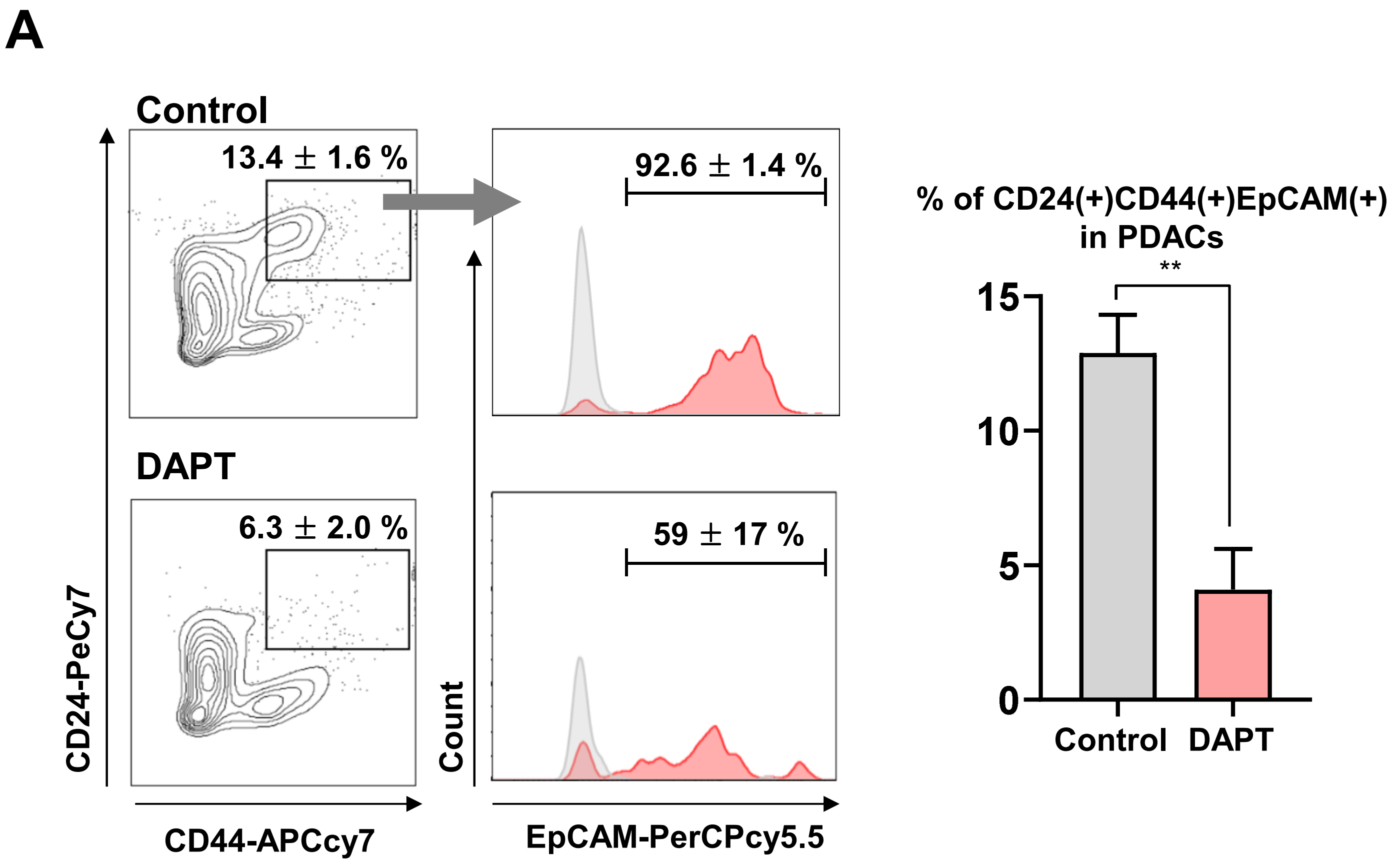
A
Control
13.4 ± 1.6 %
DAPT
6.3 ± 2.0 %
CD24-PeCy7
CD44-APCcy7
92.6 ± 1.4 %
59 ± 17 %
Count
EpCAM-PerCPcy5.5
% of CD24(+)CD44(+)EpCAM(+)
in PDACs
**
15
10
5
0
Control
DAPT

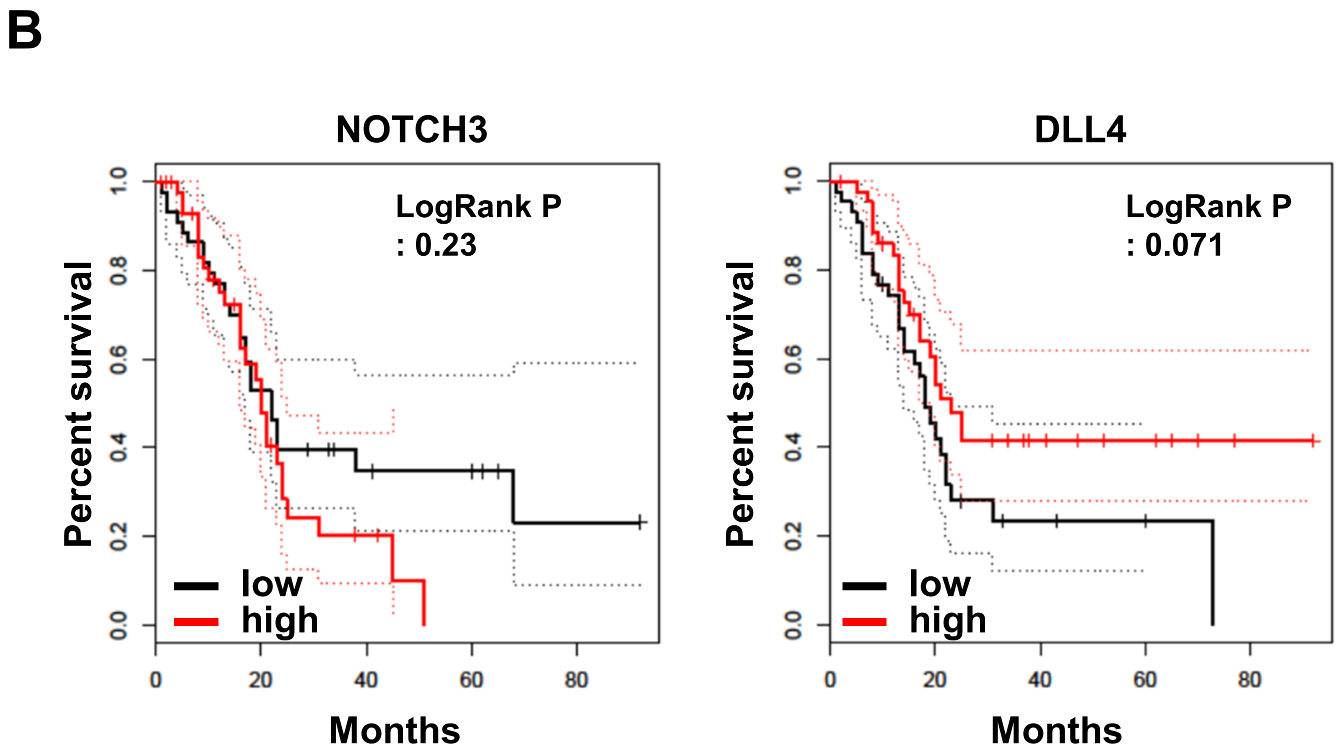
B
NOTCH3
LogRank P
: 0.23
Percent survival
low
high
Months
DLL4
LogRank P
: 0.071
Percent survival
low
high
Months

Table S1. Donor information of pancreatic cancer organoid

	Sex	Age	Source	History
Donor 1	F	65	EUS-FNA	PDA
Donor 2*	M	74	Surgical	PDA
Donor 3	F	74	Surgical	PDA
Donor 4	M	67	Surgical	PDA
Donor 5	M	63	Surgical	PDA
Donor 6	M	75	Surgical	PDA
Donor 7	M	50	Surgical	PDA
Donor 8	M	29	Surgical	PDA

EUS-FNA = Endo-scopic Untrasound-Fine Needle Aspiration

PDA = Pancreatic ductal adenocarcinoma

* = single cell RNAseq analysis

Table S2. Differentially expressed gene list according to differentiation states by trajectory analysis

gene_ID	State3 vs State1	State3 vs State2	State2 vs State1
CA2	0.16809708	2.81168E-13	5.82493E-10
CD44	4.28104E-15	0.00114374	5.92748E-08
GCNT3	0.473258198	6.50302E-08	4.25932E-07
TICAM1	0.122097983	4.00326E-10	1.22254E-06
TSPAN3	0.169855064	5.5256E-08	2.38733E-05
CTSE	0.197642949	6.46357E-07	3.90281E-05
TUBB2A	0.022822143	6.4551E-09	4.86511E-05
B4GALT4	0.952303037	0.000390032	0.000114002
PTPRR	0.796368347	1.4909E-05	0.000123093
RHPN2	0.681660701	0.000347655	0.000187234
MMP15	0.700272433	0.00044363	0.000188609
MUC13	0.211420717	5.91395E-07	0.000192119
ABHD2	0.848286219	0.000991873	0.000219203
RFLNA	0.889264534	0.000255036	0.000242943
MUC5AC	0.248206361	0.016324282	0.000243446
GSN	0.557945233	1.31523E-05	0.000248282
ARID1A	0.855328142	0.000124633	0.000261058
TIMP2	0.182174983	1.76348E-06	0.000284761
TOX3	0.79037985	0.000156894	0.000315291
KLF2	0.155684309	1.55148E-07	0.000319218
PLLP	0.371321105	0.001090849	0.00032301
LINC02381	0.040734409	0.787630049	0.000414259
B4GALNT3	0.343813532	0.028160356	0.000416693
SYMPK	0.330520025	0.121182221	0.000473396
CASP7	0.765761369	0.006211504	0.000499474
FUT4	0.702149195	4.08769E-05	0.00051191
PRKCD	0.538704424	0.008339402	0.000548366
CAB39	0.365378009	0.032429057	0.000600763
CLDN18	0.486552224	2.49273E-05	0.000608602
HPGD	0.915593824	6.71074E-05	0.000610458
SUGP2	0.423511271	0.051273173	0.000611221
MUC17	0.237915735	3.36229E-06	0.000627581
CLDN23	0.246426124	3.77568E-06	0.00062762
CXADR	0.94378867	0.000319615	0.00068267
MUC3A	0.436371467	5.57139E-05	0.000775906
MUCL3	0.18263733	0.050965548	0.000780237
LSR	0.436862232	0.000259013	0.000798489
TSPAN8	0.275255621	9.4307E-06	0.000834502
RAB8B	0.858850183	0.002349016	0.000846244
LACTB	0.810625821	0.002211458	0.000879144

AGR3	0.620397659	0.002867435	0.000900837
BCAS1	0.804196344	0.001442077	0.000916434
CGN	0.112716492	4.23281E-07	0.000944941
SEMA4B	0.079029493	5.94674E-07	0.000977713
HSPG2	0.170688524	5.87482E-06	0.001036614
UPK1B	0.470738833	0.006106949	0.001051734
PTPRG	0.862990938	0.000209718	0.00110429
SPATS2L	0.077811525	3.49892E-06	0.00115375
PKDCC	0.821802987	0.000632339	0.0012316
GOLM1	0.045614796	1.42263E-06	0.001244035
CEACAM5	0.192435265	5.42773E-06	0.001244898
SAMD8	0.621402195	0.009936831	0.001290708
CD164	0.025582454	8.94674E-08	0.001318038
DUSP14	0.430552724	0.002189479	0.001355438
FOXQ1	0.684844647	0.003418717	0.001397047
JUP	0.004964668	1.36299E-07	0.00146832
CCNQ	0.000173931	0.775763243	0.001644248
FAM102B	0.945310684	0.000502095	0.001665508
LATS2	0.021582483	2.82864E-06	0.001817099
STEAP4	0.625322951	0.000417092	0.001918481
KLK11	0.091817139	1.90034E-06	0.002035721
MFN2	0.483370938	0.022969126	0.002064088
IDH2	0.60236822	0.000443684	0.002084439
ABO	0.384194521	0.053909905	0.002087491
PDCD6IP	0.060576609	1.32128E-06	0.002116792
RAB11FIP1	0.045473365	2.99851E-07	0.002184696
ARHGEF37	0.002681518	0.251795368	0.002192555
STX3	0.345973768	0.036974643	0.002333676
S100P	0.54306624	0.087300489	0.002465976
NUDT22	0.000584379	0.733557171	0.002550448
TM4SF5	0.594121151	0.005706006	0.002866776
CIB1	0.121480396	1.56162E-06	0.002899639
DNAJC13	0.362606103	0.137642031	0.002912373
RUNDC1	0.988328014	0.000679721	0.002987359
DUSP8	0.104334908	1.17201E-06	0.003064673
EPCAM	0.049774946	6.14615E-07	0.003084473
BTNL8	0.751583479	0.002255414	0.00311427
CSTA	0.058509543	0.478258069	0.003116004
FHL2	0.880904694	0.000951957	0.003123012
PARD6B	0.769149356	0.000969492	0.003161831
ACHE	0.18210699	4.13063E-05	0.00317069
TNFRSF21	0.210210381	9.59675E-06	0.003217628
FOSL2	0.007662709	4.19641E-08	0.003276224

SCFD1	9.07731E-07	0.098749457	0.003341382
DNAJB12	0.613635255	0.079883268	0.003400397
TST	0.822657742	0.003837384	0.003423233
ANKS4B	0.9999999	0.000586399	0.003609029
MAL2	0.356746879	0.0002096	0.003647549
YPEL5	0.101606972	3.50671E-05	0.003743159
NTHL1	0.479076449	0.137480994	0.003780361
TFF2	0.876944375	0.00896723	0.003856827
TPRN	0.636134201	0.000680096	0.00391563
CCT8	0.032993116	0.637085933	0.003923585
KLF5	0.025865232	1.46194E-06	0.003982468
LMO4	0.599115257	0.003537651	0.004091296
SLC17A5	0.17813473	0.00014405	0.004107844
DUOX2	0.045500548	1.18958E-05	0.004147361
AKAP13	0.251612571	0.000191637	0.004216801
PAQR8	0.679261754	0.00141447	0.004234176
HACL1	0.019738167	0.247171006	0.004261434
INSR	0.161190719	3.38212E-06	0.004320324
MISP	0.026888182	1.57632E-06	0.004327357
ERF	0.708476355	0.003742105	0.004333509
ARHGEF11	0.822496322	0.001874727	0.004422457
CPSF3	0.168778353	0.504457052	0.004536919
DUSP4	0.415543476	0.000448933	0.004543724
SIKE1	0.236519564	0.171477976	0.004694049
PCDH7	0.019669052	0.498461605	0.004905702
UBL3	0.726367795	0.001537366	0.00514264
MFAP1	0.806785231	0.011937921	0.005144949
GNAI1	0.740278798	0.02689425	0.005171111
SAP30L	0.459247936	0.000314828	0.005309504
PSMA3	0.015379172	0.725482066	0.005387847
KBTBD11	0.15761362	0.024091363	0.0054276
TYRO3	0.202558228	0.07768517	0.005452043
ITPKC	0.874074152	0.000925008	0.005605211
LYZ	0.210049048	0.000101211	0.005638408
LPIN2	0.018302414	1.24361E-06	0.005682549
OCLN	0.30828999	0.000392329	0.005763302
CRB3	0.076416471	3.07874E-05	0.00583331
ERRFI1	0.081367968	4.154E-05	0.005836815
SLC9A1	0.98373725	0.003532771	0.00584573
CXCL16	0.113508649	2.77201E-05	0.005870202
OSTM1	0.942569994	0.004068704	0.005922088
CNPY3	0.000741379	0.884955324	0.005935238
WWC1	0.604423315	0.020178865	0.005963304

LZTR1	0.05244347	0.15194936	0.005992907
PACSIN2	0.144473472	5.4282E-05	0.006070138
NDUFA1	0.001089157	0.324539925	0.006191786
NR2F2	0.056946733	1.81329E-06	0.006232111
UGCG	0.36427346	0.000159083	0.006292856
MRPL46	0.463148322	0.398184211	0.006384237
SLPI	0.348828626	0.000626486	0.006393235
RRP1B	0.025683508	0.716654537	0.00640134
PTDSS2	0.273467997	0.034302113	0.006420744
SLK	0.080185415	5.4233E-06	0.006433106
PLEKHH2	0.234071412	0.293748514	0.006529961
CYP2S1	0.590101925	0.00531822	0.006574808
ARRDC4	0.341270506	0.000465104	0.00657526
PPDPF	0.862123219	0.064426479	0.006622917
WASL	0.217926471	0.000108465	0.006707446
CYHR1	0.819860345	0.009171228	0.006728397
EZR	0.057826631	7.38528E-06	0.006797655
DDX28	0.0120331	0.292369522	0.006855574
ABCB7	0.692658866	0.142946689	0.006986604
AOC1	0.928022028	0.000947534	0.006996271
ARHGAP27	0.864982558	0.007062365	0.007025739
LGALS9C	0.555786339	0.074594303	0.007070612
MYD88	0.124353967	1.15243E-05	0.007088124
ST14	0.047702267	2.617E-05	0.007089604
PCDH1	0.750090209	0.00163273	0.007097856
MTERF4	0.129445833	0.402771923	0.00713409
POR	0.077428418	1.29196E-05	0.00725301
HCST	0.087794173	0.043266136	0.007290374
WASHC2C	0.324071451	7.7007E-05	0.007497446
PEBP1	0.363788793	0.066814964	0.007535363
GRN	0.30016856	0.000704193	0.00779121
ISG20	0.052394972	5.13355E-07	0.007883645
AC092683.1	0.011685944	0.440624641	0.00790828
GLIPR1	0.627211019	0.270735928	0.007923145
C19orf24	0.000920054	0.424010363	0.007978716
NFKBIL1	0.540113361	0.000917474	0.008021778
OGFOD3	0.124666294	0.165177088	0.008110084
MED29	0.52053577	0.001264525	0.008119662
SECTM1	0.433869272	0.000583776	0.008207624
IGSF6	0.888665799	0.239368177	0.008207834
MACC1	0.827616835	0.005536415	0.00824928
ADAM9	0.068896098	1.62129E-05	0.008327033
HACD3	0.171340489	0.570304923	0.008431413

SPNS2	0.413277321	0.002007514	0.00847418
SMIM13	0.041123862	0.623170127	0.008531117
NOL4L	0.363920393	0.066639127	0.008581991
SLC6A20	0.873279185	0.005822573	0.008604324
CD82	0.223489373	0.000112007	0.0086087
FAM110C	0.161974324	0.180940304	0.008681885
LIF	0.737277803	0.00188609	0.008705602
SSH2	0.123626931	0.303955529	0.008734585
L3MBTL2	0.312346667	0.000155931	0.008836591
FXYD3	0.527670409	0.001592903	0.008839252
NOM1	0.012623099	0.70288185	0.009141607
HSPB1	0.621911167	0.004041873	0.009145477
RBM47	0.334752806	0.000422918	0.009385859
NPC2	0.362076349	0.000372921	0.009554141
RETREG1	0.750351216	0.027358969	0.009693845
YWHAG	0.585978395	0.001786415	0.009712408
TIMM29	0.813834924	0.006908915	0.009777822
EFCAB14	0.928162328	0.016293769	0.009795257
PPM1A	0.835769978	0.0142572	0.009819063
NUP50-DT	0.498229361	0.10110062	0.00984274
NSUN4	0.0160149	3.53663E-05	0.009872595
BTF3L4	0.091693598	0.976837351	0.00997396
BCL2L1	0.069310332	1.81881E-05	0.010028421
KDM4C	0.001657093	0.873071744	0.010072188
DHRS12	0.744274752	0.226938859	0.010090347
INPP1	0.182346215	9.86694E-05	0.010099568
RNASE1	0.512691726	0.001435517	0.010103137
FOXO4	0.401299575	0.024788201	0.010106164
ASAH1	0.130066971	0.000101282	0.010153036
CCDC68	0.58724455	0.000788297	0.010238416
CABLES1	0.326680773	0.00053512	0.010275801
AQR	0.934443513	0.004553247	0.010514727
KLF3	0.006378798	1.98676E-07	0.0105262
PRSS16	0.18301986	0.863998954	0.010549159
MTFR2	0.002984963	0.415229678	0.010600502
SOCS6	0.571326407	0.00207075	0.010649179
PLOD3	0.627983255	0.003018244	0.010724814
MICALL1	0.002334979	0.949022233	0.010767324
JUND	0.07300593	3.66074E-05	0.010826877
RHOB	0.042150613	1.55206E-05	0.01102492
PKM	0.032497148	0.351882195	0.011030604
CCDC85C	0.257101698	3.63013E-05	0.011122946
SRI	0.930568736	0.03093297	0.011216815

VSIG2	0.914597382	0.015245899	0.011252634
INTS3	0.467826186	0.070504862	0.011374395
BRD1	0.316390661	0.000331415	0.011398792
GSKIP	0.42975924	0.00133705	0.011404046
SPTBN1	0.147932993	5.14999E-05	0.011450953
TMEM30B	0.042943776	1.115E-05	0.011463873
KIF2A	0.296788296	0.201176405	0.011548671
CISD3	0.630492235	0.0723808	0.011597344
UBALD1	0.055193544	0.355082422	0.011688681
RMDN3	0.650785843	0.068139351	0.011817493
MED15	0.423585657	0.001229393	0.012068377
D2HGDH	0.381620064	0.358602841	0.012263518
PLCD3	0.44272509	0.001612256	0.012346449
SCARB2	0.222910351	0.000416633	0.012454131
TNFAIP1	0.440710624	0.00163358	0.01258893
FRA10AC1	0.087061027	0.628710557	0.012704578
ALDH1A3	0.210619974	0.108770903	0.012732652
TOB1	0.421427033	0.001514543	0.012878556
UBE3C	0.189217807	0.00019104	0.012913378
REX1BD	0.049772513	0.753030867	0.013027101
CYTH2	0.057293935	2.85423E-05	0.013077501
RBP1	0.868454613	0.360047401	0.013111599
NASP	0.060356463	0.562399909	0.013187884
LASP1	0.024145911	1.23017E-06	0.013259719
PXDC1	0.800517003	0.028086859	0.013318236
TRNP1	0.370704599	0.114693649	0.01333699
ARHGAP12	0.256199507	0.000189343	0.013375652
HLA-DMB	0.050686006	5.57686E-06	0.013408109
TENT5A	0.239634569	0.000128006	0.013431117
PSCA	0.163742442	0.141895721	0.013435287
CTSA	0.050044651	2.09981E-05	0.013447898
ARL3	0.086761186	0.26202063	0.013448857
SNRPA	0.015525681	0.693805658	0.013490481
ABCC1	0.000117331	0.243257335	0.01351958
C17orf49	0.687796539	0.415229678	0.013520353
MARVELD3	0.270401034	9.12756E-05	0.013734872
FA2H	0.89368581	0.00606664	0.013842365
PRKX	0.933544632	0.363358927	0.013869776
LCOR	0.347999873	0.000989004	0.013992158
ZSWIM6	0.682575414	0.051146176	0.013992542
CPD	0.684589928	0.006848087	0.014003435
ZBTB7A	0.148129374	0.000248806	0.014041417
TPM1	0.108713358	2.8214E-05	0.014082656

MYO19	0.053741405	0.367286609	0.014091689
SLC9A3R1	0.596243626	0.003472346	0.014270533
RAB29	0.037454116	0.52647894	0.014287276
ATRX	9.25577E-05	0.082313442	0.014377775
VIL1	0.28517736	0.038762786	0.014577361
TMEM256	0.180047412	0.369144791	0.014633377
H1F0	0.022203679	5.51081E-06	0.014659966
DYRK2	0.85660082	0.005197073	0.014817407
VAV2	0.656634686	0.019149516	0.014910898
GIT1	0.884043927	0.019626395	0.014966221
GNAQ	0.804044623	0.008854018	0.014998869
HOXB2	0.004485133	0.975866106	0.015038951
MIDN	0.009746084	2.16234E-06	0.015136023
ABCC3	0.152115117	0.369832686	0.015199234
MVD	0.006267426	0.777384966	0.015202492
MMP25	0.111434906	0.577631125	0.015224749
TMED7	0.242336809	0.00036486	0.015356335
CRIM1	0.050782127	1.47209E-05	0.015444217
SLC9A3	0.679376798	0.003390105	0.015470966
FAM3B	0.726849016	0.005524894	0.015619423
DNAJC24	0.001986291	0.615794728	0.015692816
LRRC1	0.182077328	0.000156863	0.015697952
AC020916.1	0.515237057	0.003424773	0.015721046
MCU	0.144264462	0.000185578	0.015771215
DUSP1	0.116927804	9.85713E-05	0.015837206
ANXA11	0.120827753	3.09801E-05	0.015983421
SLC45A4	0.30167655	0.001608437	0.01601246
CDH17	0.71610373	0.01569697	0.016196317
RHOU	0.607728074	0.001714303	0.016201242
BLMH	0.430179157	0.325941265	0.016242061
BRAT1	0.386713313	0.334171155	0.016287363
ESRP2	0.502276086	0.062586595	0.016336131
DUSP6	0.068730169	6.24513E-05	0.01645286
NUP107	0.971036075	0.222459284	0.016546067
LMAN2L	0.66347249	0.041008282	0.016622201
RABEP2	0.059103838	0.329864039	0.016998884
MTHFD2L	0.211940047	0.376863112	0.017032819
AC021218.1	0.518196704	0.086512621	0.017066406
DPP8	0.023792482	2.17304E-05	0.017141159
MSH3	0.50811831	0.249865923	0.017206335
CKAP2	0.113012679	0.205582839	0.017207762
NDUFAF2	0.056726634	0.573809342	0.017289019
MAP1LC3B	0.00031855	2.52167E-08	0.017369705

ZNF217	0.112842011	0.000161526	0.017381657
CASC4	0.292984195	0.000259342	0.017434212
BANF1	0.105329129	0.418672024	0.017436125
FOS	0.061986809	1.9932E-05	0.017438131
TFDP2	0.598809615	0.398792658	0.017519413
MATR3.1	0.184247139	0.64068054	0.017539587
ZNF3	0.206066751	0.118790134	0.017544505
ZNF432	0.53313191	0.123000316	0.017624751
EIF4G3	3.04474E-05	0.040280506	0.017649059
SLC37A1	0.233578736	0.00027317	0.017689837
C7orf43	0.404436032	0.384935246	0.017701487
MYO1E	0.054236974	3.97148E-06	0.017704217
CAPN5	0.420870056	0.001179244	0.017830578
TMEM135	0.629153617	0.035210002	0.01811971
TM4SF4	0.946360202	0.023939756	0.018128884
RUNX1	0.544748963	0.00321561	0.01825946
VARS	0.061858234	0.338086942	0.018267132
DUSP3	0.72142064	0.064104325	0.01837001
TCEAL8	0.637402171	0.094397981	0.018423236
SETDB2	0.798856371	0.353762929	0.018440204
FOXO1	0.127963844	8.49791E-05	0.01859526
NRBF2	0.542599766	0.139785662	0.01870874
HSPB11	0.196286735	0.816796286	0.018765181
ABHD11	0.161171378	0.000248331	0.019001825
ASCC1	0.87256635	0.151397588	0.019048261
MRPS21	0.027880925	0.994913453	0.019053201
RNF19A	0.122813945	2.84956E-05	0.019096584
EHBP1	0.009726733	0.952625717	0.019142831
CANT1	0.121170621	0.000440092	0.019306644
TRIM41	0.545366444	0.043347565	0.019453088
ENTPD5	0.827226691	0.01690206	0.019479271
MAP7	0.280618034	0.000824959	0.019493839
SIK1B	0.849530689	0.004918559	0.01949384
ITPKA	0.460717139	0.124627874	0.019546098
CERS2	0.514132555	0.003182977	0.019714254
ALDH9A1	0.235508148	0.358895134	0.019724176
ATF3	0.003612921	1.32725E-07	0.019749317
GNPDA1	0.667867797	0.043685929	0.01998563
HIST1H2AC	0.348833458	0.002853075	0.020021004
RNF11	0.602963889	0.004111144	0.020104305
R3HDM2	0.324954452	0.290865292	0.020119967
DDR1	0.163454769	0.000446214	0.020272602
TMEM37	0.266128883	0.000571732	0.020297975

NUP37	0.000486038	0.34390798	0.020398428
STK39	0.822794213	0.006613635	0.020457287
BX255925.3	0.218613495	0.000554982	0.020489249
MTCH2	0.954459645	0.082866548	0.020543233
RAB22A	0.128302049	0.000375384	0.020559819
RBM10	0.000483224	0.170173791	0.020587195
TDG	0.007567716	0.857516739	0.020591618
DBN1	0.132503914	0.307905205	0.020706704
NTN4	0.43502814	0.003402882	0.020735627
MYO15B	0.247695818	0.000148956	0.020779116
PIK3CG	0.02236426	0.254662063	0.020839147
SLC38A10	0.184816967	0.000458496	0.020916516
PRMT1	0.080127032	0.799214191	0.021008374
FMO5	0.441321587	0.044409325	0.021014578
NOL9	0.04262408	0.453689805	0.02108488
STX7	0.134684527	0.000253616	0.02108609
FAM13B	0.033509198	0.967596988	0.02112443
ARPC1B	0.050545153	0.846622961	0.021144292
PRR13	0.587816653	0.006154835	0.021279712
SNX9	0.384915461	0.002290143	0.021279912
NEK3	0.005836303	0.976378487	0.021434695
FUT2	0.088704607	7.61125E-05	0.021434764
GPR108	0.42896575	0.001278214	0.021536416
RGL1	0.206522766	0.920649543	0.021545004
SERPINB9	0.028316126	0.932849743	0.021690015
METTL15	0.670348165	0.006898288	0.02169654
SCD	0.019216568	0.47202912	0.021775886
AMDHD2	0.276439576	0.192545615	0.021808601
FYCO1	0.910086543	0.008606324	0.021846038
B3GNT7	0.736345393	0.133043574	0.021919438
ANAPC5	0.153984469	0.520106786	0.021972485
SMPD3	0.571162486	0.002698399	0.02215642
EBPL	0.016363039	0.932096074	0.022410859
CHST4	0.549336157	0.005046451	0.02245312
ANKRD29	0.069875381	0.000120984	0.022468765
SFT2D1	0.964160363	0.055867631	0.022513402
CDH3	0.000147614	0.079023581	0.02258018
GRAMD2B	0.59525104	0.039079146	0.022677647
DVL3	0.066602076	0.000247136	0.022917003
RAB11A	0.292819696	0.001940423	0.022934731
RAVER2	0.598263326	0.003641527	0.022957223
SLCO2A1	0.637329986	0.003352653	0.022993813
AGAP1	0.79544653	0.025885677	0.02305792

SYTL2	0.599467436	0.275207827	0.023142762
NPAS1	0.267167124	0.483367428	0.023180249
SCRN1	0.120766983	0.000319704	0.023187045
CLRN3	0.238041559	0.32527635	0.023291451
RIOK3	0.243173097	0.000680788	0.023577463
AC007952.4	0.692844841	0.120437077	0.023597339
UBR5	0.029416672	0.832185911	0.023951217
TUBG1	0.127909073	0.524725765	0.023965445
TUFT1	0.011033659	2.34073E-05	0.024249836
UBE4A	0.03826263	3.62058E-05	0.024278672
IFI44L	0.001032784	0.358986359	0.024316658
TSPAN1	0.216779299	0.00056886	0.024336405
NCOA4	0.270218391	0.000904742	0.024343018
DOK2	0.095652649	0.2286996	0.024514524
TBCB	0.136070185	0.393850447	0.024711279
RHOBTB1	0.085710271	0.000193646	0.024728917
CEACAM6	0.072587694	0.000146424	0.024771491
ZFP36L2	0.038139294	4.16821E-06	0.024789222
DDX23	0.032920644	1.44063E-06	0.024835899
EXOC8	0.831721406	0.002766298	0.024979523
TRMT10C	0.000366142	0.226792155	0.025016212
MSRB2	0.763102097	0.022633604	0.025153517
NIT2	0.001302617	0.162679143	0.025155407
ACTR3C	0.234231578	0.120844576	0.025244399
PRR15L	0.547907849	0.002755413	0.025285196
C21orf2	0.090883927	0.380863066	0.02535268
CCNL2	0.007195752	0.760937014	0.025502502
CCL15	0.22429483	0.331166084	0.025514535
RIPK4	0.11606726	0.000233104	0.025518542
ALDH3B1	0.574369362	0.050484007	0.025632317
WASHC5	0.188010362	0.855422938	0.025737775
WDR24	0.235243156	0.000665761	0.025857029
UBAP2L	0.808645481	0.120030001	0.025861076
IWS1	0.996712658	0.050202844	0.025896297
PHF2	0.3598804	0.002613387	0.025910695
RNF130	0.352085525	0.624271724	0.025978694
DPP4	0.130939283	0.000506084	0.026007468
TMA16	0.515029168	0.374813774	0.026010225
NCK2	0.757596397	0.272241959	0.026024131
USP8	0.814554243	0.022924874	0.026096081
FAM171A1	0.433606993	0.362710236	0.026102954
WDR74	0.08394985	0.530965352	0.026131438
MESD	0.009981378	3.77078E-06	0.026253483

MRPS27	0.008536099	0.881539508	0.026304777
SLC35E2A	0.842544955	0.339959599	0.026352265
LDLR	0.215839112	0.000113194	0.026365155
LGR4	0.161116537	0.000273721	0.026369464
SLC15A3	0.173083202	0.00038074	0.026422002
FKRP	0.004781931	0.752472013	0.026457686
TIMM17A	0.020989492	0.78791926	0.026499317
EBP	0.043459347	0.789908776	0.026526739
GAPDH	0.039187714	0.455908982	0.026574188
PPP3CC	0.777584693	0.09662428	0.026634539
BMP2	0.216333889	0.000627548	0.026804092
CCL28	0.197365507	0.000357486	0.026890951
PLAT	0.003526778	0.611568431	0.026997763
TGFA	0.602787135	0.003382091	0.027021544
KCTD3	0.627333245	0.007542003	0.027169581
WHAMM	0.199379745	0.000710601	0.027237995
FILIP1L	0.149079746	0.445034325	0.027334099
ZNF512	0.191158049	0.415229678	0.027429119
TMEM185B	0.768874789	0.006888261	0.027659999
UBE4B	0.534159197	0.135931927	0.027673732
C3orf58	0.401299575	0.01915491	0.027829078
CREB3L1	0.418745993	0.00383996	0.027973693
CHPT1	0.816480272	0.062722611	0.028013618
ESYT1	0.01501225	0.986178089	0.028061331
SREK1	0.464319953	0.44025864	0.028460484
TMED10	0.574136862	0.004630132	0.028485515
CAPN9	0.658521231	0.044686166	0.028492954
ZFP64	0.68417533	0.25216226	0.02849796
AXIN1	0.008744264	0.902805031	0.028550281
CRIPT	0.694736885	0.018787982	0.028577089
ZNF532	0.007627788	0.908886132	0.028691239
CEP120	0.908557576	0.023933756	0.02874441
RAB12	0.697103799	0.011800011	0.028776098
PIH1D1	0.091093071	0.547186999	0.02888347
CTNNB1	0.628087035	0.008328992	0.029010113
ANKS3	0.027212298	0.415229678	0.02909427
MAP3K21	0.176056434	0.593385431	0.029122558
VSIR	0.097227027	4.03186E-05	0.029149893
PTPN4	0.109485868	0.60853106	0.02924718
ABHD3	0.657907575	0.008872531	0.029282611
MSMB	0.484821275	0.018028424	0.029367526
C8orf76	0.364607427	0.00183599	0.029424811
TMEM134	0.874706073	0.020047089	0.029466594

EPS8	0.008343151	1.52644E-05	0.029475307
DEPP1	0.416339342	0.003052099	0.029551484
CPEB2	0.462205107	0.164476987	0.029584055
MYADM	0.000220928	0.116981445	0.029590235
SPSB1	0.008794384	0.876051106	0.029687573
GTPBP1	0.693482688	0.151033987	0.029736769
CCDC159	0.163090191	0.291811353	0.029928621
RABAC1	0.036352589	0.837008708	0.029985311
RPF2	0.666852035	0.147553991	0.029992484
THAP3	0.002377164	0.40341115	0.030071756
STK38L	0.667033849	0.197546675	0.03010734
LINC00342	0.000953936	0.23001642	0.03013871
WDR20	0.946544879	0.011705609	0.030145556
PVR	0.124629153	0.000257655	0.030319331
PIP5K1A	0.591812281	0.007338104	0.030329862
ITGA1	0.031605165	0.806898493	0.030529636
SMCO2	0.008385999	0.9999999	0.030534065
RGS19	0.013125283	0.935103339	0.030602656
GCSH	0.934911491	0.150839648	0.03064826
BLOC1S1	0.759530061	0.014933115	0.030728933
DPY30	0.007981729	0.623496109	0.03078516
LINC02001	0.502116445	0.415229678	0.030787255
GPBAR1	0.358667164	0.169998577	0.030787407
TMEM128	0.522188954	0.003547255	0.030852442
CHCHD4	0.430836543	0.199341747	0.030929379
MIRLET7BHG	0.018933912	0.8166734	0.03097033
RAB3GAP1	0.748007847	0.020951311	0.031140705
FRK	0.351826819	0.001939559	0.031182961
TXK	0.071539944	0.249441007	0.031193465
MPP3	0.008652843	0.9999999	0.031216885
SCAND1	0.249118327	0.000600172	0.031272734
TMEM70	0.178611082	0.285764639	0.031340087
TJP3	0.460234346	0.004062675	0.031356729
ZBED1	0.593391628	0.009711088	0.0314028
MIB2	0.008450293	0.989077107	0.031414373
NISCH	0.597243069	0.097985178	0.031468041
DNAJC3-DT	0.016028624	0.757286534	0.031513022
MLLT6	0.002284424	0.379892115	0.031536826
NAA30	0.263817392	0.59523031	0.031605031
ERCC1	0.074349006	0.773761436	0.031719582
OPA3	0.397582974	0.041605243	0.031744883
TACC2	0.894343308	0.018341742	0.03178447
NDUFB11	0.268064231	0.429075788	0.031854877

SLC4A2	0.597743867	0.171616112	0.031872255
PELI2	0.96286259	0.016599886	0.031883107
MARCKS	0.057780622	4.72268E-05	0.031960114
LTB4R	0.0151836	0.415229678	0.032013331
CD55	0.002627975	1.30179E-07	0.032041914
RHOA	0.195524511	0.000178351	0.032088579
SRM	0.112217972	0.577388281	0.0321198
GIT2	0.234348463	0.746481625	0.032237479
DNAJC5	0.941794308	0.056874867	0.032242151
SLBP	0.046668471	0.795318175	0.032280048
IRS1	0.5099064	0.006768962	0.032314758
CLDND1	0.139439345	0.333312909	0.032530737
ZNF559	0.401299575	0.02441866	0.032558023
C2CD4B	0.193367509	0.156382599	0.03264164
HADHB	0.004680875	0.558636532	0.032648801
P3H1	0.074739241	0.463678098	0.032673628
SARNP	0.376572494	0.881898055	0.032770684
COPE	0.00650923	0.472956091	0.032843017
C9orf64	0.265064584	0.002118584	0.032882877
RFX5	0.415361122	0.003592793	0.032948074
CYP2C18	0.014679218	4.19015E-05	0.032974023
TUSC1	0.239003352	0.001988422	0.033013174
WNT7B	0.042820395	0.633352713	0.033176223
SAV1	0.131619233	0.000879114	0.03328458
TUBA1A	0.02395565	0.570035739	0.033440398
E2F3	0.000626776	0.219300141	0.033516766
ADM	0.944230979	0.024267624	0.033516922
METRNL	0.339518614	0.002778295	0.033551888
P4HTM	0.696403702	0.006850024	0.033668564
SMC2	0.858236973	0.091324284	0.033701881
CCDC186	0.078652919	9.04353E-05	0.033821551
METTL26	0.592181423	0.104841401	0.033822306
EDEM2	0.025375454	0.891132106	0.033862395
CFI	0.17211621	0.147659783	0.033914006
CDCP1	0.024746414	2.78418E-05	0.033959059
ABCD1	0.563337142	0.005846667	0.033990217
PML	0.000106065	0.051920656	0.034041398
TMEM126B	0.663154308	0.320284318	0.034086474
MEPCE	0.001347289	0.2636443	0.034129349
C2CD4A	0.105018494	0.157248687	0.03414376
FAM104A	0.940069758	0.080040408	0.03419378
FLNA	0.007697484	0.702052133	0.034199863
PKP2	0.449921356	0.103555004	0.034255879

CBX4	0.775214011	0.040621808	0.034367436
LYPD3	0.009931337	0.9999999	0.034407859
CAPG	0.341822486	0.336003353	0.034453524
SIK1	0.498464616	0.082411688	0.034600998
UFSP1	0.815806783	0.018173363	0.034612921
KLF6	0.055120144	0.000261787	0.034664379
LMO7	0.001430339	2.1787E-07	0.034689948
HACD4	0.050706661	0.415229678	0.03482098
HR	0.9999999	0.012559443	0.035005584
ACTL6A	0.040036733	0.987971817	0.03501319
APH1B	0.314918076	0.53701047	0.035021906
DDX10	0.019639124	0.813745737	0.035063024
TRIM15	0.095947642	7.85128E-05	0.035080279
DDX3Y	0.012307512	0.774745726	0.035118335
AL691432.2	0.401299575	0.652014622	0.035190903
B2M	0.024557489	1.53241E-05	0.035327352
NEK9	0.003158525	0.447660191	0.035337283
ARHGAP17	0.75970682	0.010618233	0.035381513
CCR1	0.932263868	0.415229678	0.035430026
ANP32E	0.352091038	0.08865566	0.03543724
ILVBL	0.825653343	0.141063893	0.035448887
STEAP1	0.00960032	0.875826039	0.03545525
OSR2	0.851232608	0.03860277	0.035466362
FAM114A1	0.581606115	0.011465462	0.03548111
SAXO2	0.9999999	0.012813944	0.03553182
F2RL1	0.066805415	1.89845E-05	0.03573126
MAFF	0.002214774	4.12589E-07	0.035752349
BATF	0.323793359	0.327981955	0.035817347
GIPC1	0.856349652	0.033243166	0.035866464
PLCB4	0.9999999	0.013056376	0.03603065
SMIM5	0.996185378	0.07165419	0.036036523
MKRN1	0.319894204	0.001593361	0.036180732
MIR29B2CHG	0.011015288	0.839062218	0.036210741
STRN4	0.693772915	0.245947423	0.036248907
ZBTB17	0.812680534	0.035531175	0.036274167
SSBP2	0.429558269	0.343954338	0.036286597
RNF20	0.000111391	0.109730028	0.03632084
KHNYN	0.293955895	0.00144127	0.036323618
RPUSD4	0.902613724	0.048597411	0.03636682
INPP4B	0.00513703	0.457078245	0.036387858
PGS1	0.008906838	0.912536027	0.03644184
APIP	0.254321125	0.878477533	0.036470967
ZNF324	0.014565311	0.918253569	0.036480219

RTN4R	0.034354164	2.99352E-05	0.036533887
LDHB	0.230414489	0.13001061	0.036609132
C1orf21	0.360040432	0.21413535	0.036637438
ADGRF1	0.067826727	6.98594E-05	0.036702965
DSTN	0.151500591	0.000122968	0.036715638
TRAPPC5	0.010905091	0.9999999	0.036758592
BBS12	0.401299575	0.068893887	0.036917892
MDN1	0.355909741	0.44471921	0.036963544
AC090912.2	0.234641166	0.112946869	0.037011437
SEZ6L2	0.829694087	0.048649388	0.037095528
NPM1	0.141350113	0.570488077	0.037104966
AKR1C3	0.467679326	0.384970598	0.03713386
ANXA6	0.031338836	0.688149108	0.037135744
CAMK2D	0.035009605	1.47202E-05	0.037217076
HLA-F	0.067181203	0.000228378	0.037314625
WDTC1	0.875408733	0.026052283	0.037387577
SPG11	0.208987747	0.902048854	0.03739568
CFDP1	0.421762444	0.007430136	0.037413031
SOWAHC	0.231922382	0.001074727	0.037589576
TMEM168	0.338123817	0.002816614	0.037606022
SRGN	0.400013876	0.000348146	0.037637746
MRPL41	0.015310095	0.841625249	0.037909934
UBE2O	0.001333774	0.194483016	0.037929772
AC025263.1	0.122384236	0.474594373	0.037942928
TRIM21	0.030579798	4.94421E-05	0.038084649
TIA1	0.001490418	0.281255391	0.038092864
DUSP5	0.382363331	0.002307034	0.038174912
HDAC8	0.001374057	0.249367603	0.038209796
SUFU	0.278077597	0.047777771	0.038226375
CD2	0.193746587	0.146906172	0.038404251
ACE2	0.431659194	0.480768719	0.038446051
SBNO1	0.314913459	0.001926345	0.038464812
POLR3B	0.091157153	0.324890394	0.038542041
BLVRA	0.530992168	0.19297696	0.038570739
SELENOW	0.610368735	0.02379015	0.038801176
DPM2	0.000257976	0.154631058	0.038941982
NOMO1	0.150708802	0.344827903	0.038997486
ATP2B4	0.000103227	0.019743459	0.039202341
RNF186	0.65761219	0.020972238	0.039443904
ZHX1	0.603888324	0.223467436	0.039587332
SHROOM3	0.018749583	1.21154E-05	0.039639494
ZRANB2	0.002483776	0.254692831	0.039655606
CFAP298	0.011510338	0.423490576	0.039791077

THOC3	0.634767574	0.125658907	0.039841023
KLHDC7B	0.012285226	0.9999999	0.039989061
RNASEH2C	0.247870811	0.484387181	0.040030872
CFH	0.044098134	0.68057781	0.040042251
CLPB	0.000608352	0.118229097	0.040073213
MXRA5	0.088898087	0.366659076	0.04010026
BMPR1A	0.388678066	0.003176135	0.040126342
SMIM22	0.027258205	5.69623E-05	0.040164706
SLC16A7	0.97956708	0.048313766	0.040208895
EXOC6B	0.674661003	0.013199106	0.040261699
AREG	0.636829785	0.013752814	0.040291761
HECTD1	0.555924133	0.367055686	0.040403101
FGFR1OP2	0.003702457	0.345169577	0.040413077
NIT1	6.43661E-05	0.079768899	0.040625764
CNIH1	0.317719568	0.514178847	0.040711331
NCAPD3	0.575384155	0.190873154	0.040773245
AVPI1	0.54716063	0.003410409	0.040780338
GKN1	0.423969979	0.004490237	0.040831775
USP2	0.401299575	0.124966747	0.040851716
TRAF3IP2-AS1	0.235068737	0.144239917	0.040878035
PSAPL1	0.342940171	0.55806834	0.041148125
CAPRIN1	0.000249739	0.148509403	0.041226592
ZNF322	0.855296653	0.055146636	0.041288475
AC016831.7	0.617779939	0.119181623	0.041348939
TTC8	0.291951531	0.881715262	0.041349818
AP000866.2	0.9999999	0.015734792	0.041397608
CDHR5	0.952234634	0.025555321	0.041411325
ITGB5	0.973054966	0.020962854	0.041617448
COQ5	0.671202392	0.209984295	0.041697792
TRAC	0.029457734	0.430840167	0.041738361
LIPH	0.003247786	3.33396E-07	0.04176673
PSD3	0.707314563	0.081216872	0.041797082
TRIM66	0.013091396	0.9999999	0.041827309
CAMK2N1	0.2510727	0.000431906	0.041885544
PLAC8	0.751907368	0.01108122	0.041923273
MMP9	0.863760272	0.05520448	0.041954541
PRRC2B	0.048526232	0.900661498	0.04203945
TUBA4A	0.051176667	0.912958175	0.042059302
HDAC7	0.020388245	0.836905129	0.042097264
ACOX1	0.72667967	0.072534945	0.042138329
LINC00926	0.708852635	0.032675931	0.042178695
LARP4B	0.239581414	0.000351118	0.042217375
ATPAF1	0.984136988	0.02871716	0.042436307

NFKBIE	0.937297407	0.069107131	0.042462344
MED22	0.16474172	0.157510387	0.042579391
CNOT8	0.104360178	0.575311583	0.042684985
MFSD10	0.067517982	0.862390218	0.042816037
CAMSAP2	0.001341405	0.29667372	0.042897855
ATP8B1	0.918208095	0.024801496	0.042924545
CLDN4	0.01009016	2.17479E-05	0.042952381
AL441992.1	0.183338462	0.813041159	0.042969481
CDC42EP5	0.062993246	6.02874E-05	0.043010362
NRP1	0.016090602	0.790332127	0.043026451
SMG8	0.23259715	0.153309525	0.04304173
SMAD5	0.333573332	0.449547978	0.043088944
MFNG	0.43758366	0.20265705	0.043313706
SFR1	0.7474175	0.009444043	0.043373675
SPATA2	0.240142374	0.000785042	0.043438601
BUB1	0.373792955	0.149495805	0.043447898
SPATA6	0.11511207	0.791936007	0.0434639
PLS1	0.106979415	0.000175459	0.043643415
AC020656.1	0.576554475	0.187804922	0.043649216
DCBLD2	0.029484905	0.838692321	0.043736266
NCOA7	0.082936652	0.000173406	0.04376857
PRSS8	0.031030798	6.76172E-05	0.043791919
SIAE	0.594312803	0.006547674	0.043864908
DEGS1	9.72805E-05	0.025905745	0.043895774
PARP8	0.36514114	0.003952197	0.043932978
TBC1D22A	0.437320138	0.260655453	0.043982999
PIGA	0.771431787	0.016467398	0.044008887
LAMTOR2	0.000486668	0.060147543	0.044031323
NFX1	0.398223509	0.37951549	0.044225035
ASXL1	0.00555868	0.463306389	0.044461018
FGF2	0.005487637	0.225200933	0.044505212
CDX2	0.071078194	0.000336876	0.044610006
PDS5A	0.493265581	0.28311099	0.044641824
CDC20	0.109097254	0.247605833	0.044655419
DPYD	0.000165	0.02154224	0.044819946
DAG1	0.063702775	0.00028046	0.04484547
WDFY2	0.010158735	0.648512255	0.044859661
LDHA	0.027826759	0.974449188	0.044967207
TBK1	0.561347378	0.01300772	0.045042971
ABHD17C	0.055767715	1.73264E-05	0.045094412
ACAP2	0.78998957	0.402109732	0.045106302
DARS	0.00046603	0.091143139	0.045229518
ZC3H7B	0.123360442	0.981666728	0.04537576

PEX5	0.9999999	0.017800489	0.045380045
HMG20B	0.221880477	0.002657295	0.04546333
CD53	0.007842222	0.660431434	0.045505032
ITGB7	0.004685066	0.340090347	0.045555873
TTBK2	0.005947217	0.601338908	0.045628572
LTBP1	0.868662963	0.03872973	0.045687168
ESRP1	0.67731424	0.075734894	0.045768875
CYTIP	0.769996278	0.017326447	0.045813453
SNAI3-AS1	0.014898865	0.9999999	0.045834733
NHS	0.965110682	0.016787117	0.045842573
EHD2	0.348402912	0.225753128	0.045988929
SNU13	0.018452587	0.528544877	0.045997843
RAB27B	0.761552889	0.022943829	0.046082248
OPLAH	0.419998865	0.216517857	0.0462172
CLDN3	0.314973014	0.001206086	0.046246907
SIRT1	0.554854776	0.132049675	0.046283756
ARL6IP4	0.055668813	0.873253811	0.046387687
KAZALD1	0.838825558	0.024585953	0.046450944
ABI1	0.246126786	0.003180593	0.046483896
ACAT1	0.12804618	0.508831865	0.046679685
APC	0.243735454	0.002127555	0.046807575
KIAA1522	0.620973474	0.014891843	0.0468503
SORBS3	0.835714837	0.022322543	0.047217604
TCN1	0.17646931	0.002082919	0.047286299
HOXA3	0.820366654	0.191496499	0.047416218
TMOD3	0.479724877	0.010608425	0.047626977
RRAS2	0.49261771	0.016113095	0.047744119
AC073332.1	0.237674218	0.213179665	0.047783305
LIMA1	0.331538811	0.005020337	0.047980177
GRK3	0.207327551	0.329245825	0.048043931
POLE2	0.249507312	0.415229678	0.048316946
KLK6	0.032577687	0.464444849	0.048326955
HOXB3	0.087640542	0.000266414	0.04836118
SEMA6B	0.017354415	0.976378487	0.048487043
AC105446.1	0.016140676	0.9999999	0.048506916
BRD3	0.560085851	0.105077543	0.048568042
AEN	0.947640497	0.035673558	0.048651556
TNFSF9	0.936470552	0.067714654	0.048707125
KLHL15	0.037342443	0.415229678	0.048736596
RPL4	0.142503685	0.578732959	0.048796256
ZNF341	0.510569531	0.415229678	0.048804235
FRRS1	0.016292399	0.9999999	0.048829277
CYP1B1	0.841461009	0.062357423	0.048965789

IQSEC1	0.354011043	0.525196867	0.049080779
SIVA1	0.332744952	0.501289202	0.049086176
DDX17	0.005008511	0.289975134	0.049088901
GARS	0.022329751	0.848691154	0.04912752
CHD7	0.498136944	0.261453488	0.04914666
ARL5B	0.857075123	0.05270274	0.049154521
SNAPIN	0.998832366	0.071139813	0.04916719
RASAL3	0.284642828	0.3080137	0.049259049
RGMB	0.401299575	0.054487938	0.049304358
SPA17	0.06994097	0.785077932	0.049514976
KCNAB2	0.003904641	0.296842032	0.049618706
IL7	0.227792543	0.00298842	0.049630056
FKBP11	0.187968823	0.708953441	0.04986085
SGO2	0.468123883	0.316489669	0.049906336
PPP1R26	0.32600318	0.004952714	0.050257703
VSIG10	0.055487102	0.000295629	0.05036984
CXCL17	0.047491532	0.391541448	0.050472416
TRAPPC11	0.705600972	0.014036814	0.050588636
AC026401.3	0.017144369	0.9999999	0.050623751
TMEM219	0.311779734	0.006699307	0.050804814
SAYSD1	0.507234295	0.04288307	0.05087855
HLA-DQB1	0.260613903	0.005877895	0.050900867
RAB2A	0.241992314	0.00155599	0.050980156
RPL10	0.018460827	0.812602429	0.051123536
YTHDF1	0.394156261	0.00816543	0.051383334
EXOC1	0.792134821	0.033132872	0.051517074
PIP4K2C	0.128581636	0.000597906	0.051575846
MAGI3	0.027712742	0.000225315	0.051717279
EEF1A2	0.091571067	0.000216306	0.05172631
AC103591.3	0.003201348	0.393286766	0.05175088
VILL	0.300001023	0.007239019	0.051903201
RPS4Y1	0.01591761	0.706900767	0.051978897
ACTN4	0.009286553	3.00648E-05	0.052017718
PICALM	0.032489452	0.000145018	0.052337415
CEP57	0.022471144	0.751588202	0.052351766
CAND1	0.500048654	0.012942308	0.052663247
MED20	0.835476985	0.02000061	0.052865072
AZGP1	0.065404136	0.000126203	0.052884313
BDKRB2	0.297121152	0.003570952	0.052966073
MRPS15	0.007185195	0.451810844	0.053139513
TAF13	0.543868422	0.009142389	0.053163061
APH1A	0.698210352	0.046098368	0.053252329
CCDC122	0.043848425	0.415229678	0.053359177

EPN1	0.015852368	7.26481E-06	0.053454683
SPATA7	0.768020305	0.042151387	0.053519259
PSIP1	0.010762437	0.600126791	0.053569716
LITAF	0.069821464	0.000131126	0.053647244
GPR35	0.858300311	0.046215364	0.05385459
ECHDC3	0.018727399	0.9999999	0.053891821
FEZF1-AS1	0.489473045	0.008357829	0.053894313
RNF135	0.026132161	0.732984129	0.053975478
LINC01003	0.368888133	0.005519161	0.054180565
MAN2C1	0.760240315	0.01534632	0.054284933
RAB17	0.967976981	0.033471563	0.054328152
STX1A	0.000265789	0.207105805	0.054411754
KIF3B	0.27448418	0.001631419	0.054521426
MYO1B	0.57103882	0.014001396	0.054840421
FAM3D	0.00939179	1.19369E-05	0.055080106
SLF1	0.039710722	0.66764579	0.055192641
AP3M1	0.013327091	0.663753268	0.055223483
CHCHD2	0.008533688	0.38704801	0.055322047
UBL5	0.001823107	0.12911217	0.055534358
TTC22	0.042793017	0.000159202	0.055620515
NEAT1	0.013400398	0.488080774	0.05587107
CLIC5	0.034840419	7.96546E-06	0.056003898
NCEH1	0.089671054	0.000184418	0.056009743
SPTSSA	0.00525759	0.36436035	0.056090405
SH3BGRL3	0.008521623	0.402161723	0.056128799
TRAF4	0.057496826	0.000362998	0.056377238
APP	0.003873718	8.87911E-07	0.056398575
RARA	0.231386643	0.001783189	0.056413615
DSG3	0.028519024	0.247235212	0.056577918
HIST2H2BE	0.049450978	0.000163419	0.056636422
AC005550.2	0.401299575	0.047671317	0.056922898
GPR153	0.998897365	0.027830184	0.057040468
TMEM54	0.235937821	0.001449496	0.057101261
GMEB2	0.50530042	0.005843397	0.057353813
KIF5B	0.278638902	0.004575659	0.05743193
PSMD5	0.423408094	0.005606303	0.057634362
HDAC10	0.402099489	0.006201872	0.057723409
MYH10	0.976692737	0.026332202	0.057754912
NRROS	0.020681172	0.9999999	0.057818409
PELI1	0.658999292	0.020308464	0.058051262
LGALS1	0.139689194	0.006869785	0.058380905
GTSE1	0.022774929	0.833750565	0.058382482
C1orf198	0.764931684	0.019190192	0.058390824

MORN3	0.020975197	0.9999999	0.058399958
TMEM204	0.030253401	0.415229678	0.058409167
HEXB	0.125583531	0.001357623	0.058706352
YWHAZ	0.045939953	6.55942E-05	0.058885318
CCNK	0.418792298	0.012493699	0.059028377
SLC39A14	0.773737519	0.017918808	0.059066587
NAA60	0.354648644	0.01090635	0.059410471
SP2	0.021065508	0.000582699	0.059450633
GBP3	0.202183227	0.001724857	0.059556663
MYPOP	0.020731031	0.968821775	0.059679253
NIF3L1	0.744456686	0.027355183	0.059759418
HAVCR2	0.021870883	0.9999999	0.06015741
MID1IP1	0.008691961	0.442334142	0.060172856
CCDC124	0.021541684	0.683420086	0.060191863
KANTR	0.021892169	0.9999999	0.060198923
WLS	0.413159647	0.006154718	0.060390839
PPP1R7	0.338645078	0.006609708	0.060434407
RHNO1	0.453754792	0.009215822	0.060440491
NUDT7	0.012181554	0.550191433	0.06064807
LINC01215	0.022125595	0.9999999	0.060653419
GATA3	0.024229532	0.9063136	0.060779074
IFT140	0.926871385	0.045562724	0.061455103
WDR48	0.780516412	0.022473396	0.061462814
FAM98A	0.001860685	0.188720314	0.06149218
TNFRSF10B	0.019764927	0.684862116	0.061612507
ITGAL	0.031689799	0.878320377	0.061632895
BOD1L1	0.03523581	4.44363E-05	0.061792616
DNAJC30	0.002173467	0.47020332	0.06192769
IFNAR1	0.06262533	2.81705E-05	0.061982653
IPO8	0.920190592	0.032275656	0.062044173
NPTN	0.547822493	0.009127533	0.062062578
WAC	0.034073132	8.6459E-05	0.062143956
MTMR2	0.360927565	0.003559644	0.062241003
TAF1D	0.010821717	0.593930834	0.062363599
SULT1A1	0.381377663	0.005816152	0.06243327
QTRT2	0.032241272	0.676817501	0.062604873
C2orf68	0.710376194	0.01542356	0.062643185
CHAC1	0.511922524	0.013444539	0.062691184
RHOD	0.132018636	0.000706496	0.062787478
BIRC3	0.125139059	0.000206551	0.063149234
CARD8-AS1	0.039932014	0.415229678	0.063197011
VPS13D	0.010958654	0.68218018	0.063306653
WASF2	0.007111075	1.22182E-05	0.063600951

RPRD1B	0.58280574	0.01912667	0.063609167
CD24	0.637202884	0.029343127	0.06381049
WDFY3	0.653212874	0.013127998	0.063900223
N4BP2L1	0.46361537	0.006471729	0.064148386
CCDC69	0.004113893	0.203454471	0.064153879
PIGR	0.047555745	0.000201072	0.064270885
LIMD2	0.043623623	0.702740329	0.064313493
RPL12	0.043353354	0.926520159	0.064353248
ORC2	0.015092622	0.72310482	0.064545088
RNASET2	0.03259887	0.622425282	0.064572587
ZNF490	0.024195321	0.9999999	0.064625751
WWC2	0.831417096	0.032078662	0.064680722
ITGAV	0.601657125	0.016504222	0.064850353
TOR1B	0.001412785	0.041254697	0.064994917
YWHAH	0.121612052	0.000843442	0.065144211
ZDHHC2	0.03345104	0.949983435	0.065192925
SCAF1	0.737637555	0.02437357	0.065235183
CCDC71	0.97568242	0.026803462	0.065285244
B4GALT5	0.052268372	0.000185827	0.065378829
RAB35	0.782206912	0.037444714	0.065929959
TRIM31	0.001765437	4.65523E-08	0.065937089
SPINT1	0.002694593	3.8805E-06	0.066252163
MAT2B	0.068603809	0.000262493	0.066555173
B3GNT3	0.07566622	0.000645448	0.066651721
LMTK2	0.004867587	2.36299E-05	0.066679305
GYS1	0.016526436	0.716143773	0.066715337
CYB5R4	0.286101252	0.003206113	0.06687639
TP53INP2	0.183149688	0.001069577	0.066892912
ZDHHC13	0.017444274	0.000109102	0.067122096
SSBP3	0.144666109	0.001106764	0.067382323
FUBP3	0.298403708	0.002521488	0.067823923
SP4	0.003540004	0.225200933	0.06784723
CCL18	0.040876484	0.744861787	0.067970211
CITED2	0.764180816	0.022815357	0.068041674
CPOX	0.029476656	0.860977355	0.068254173
ARHGAP6	0.056243084	0.000336545	0.068263427
USP20	0.004339118	0.093406877	0.068302411
CCDC18	0.017043093	0.74807678	0.068303966
CREBZF	0.001393332	0.075288605	0.068549478
ICMT	0.034920928	5.43523E-05	0.06859481
RRNAD1	0.004723053	0.468829996	0.068796033
CYTH4	0.026427947	0.9999999	0.068804037
MAN1A2	0.037092095	0.860956144	0.0690693

SYT13	0.257758391	0.005036909	0.069155325
GALT	0.021428201	0.701152474	0.069208077
ESD	0.338226202	0.042869092	0.069346978
TACC1	0.448619251	0.007687361	0.069377369
BACE2	0.408228076	0.016665437	0.069414795
FAIM	0.037251433	0.976378487	0.069443186
FAM222B	0.138570324	0.000432287	0.069461728
LAMA5	0.003758387	0.182114137	0.069525876
MIR3945HG	0.9999999	0.031753013	0.06988082
AL357033.4	0.9999999	0.031847428	0.070036018
TRIM14	0.04922773	0.75578487	0.070064141
GPR68	0.019892317	0.671446916	0.070073409
MYRF	0.012967494	5.04333E-06	0.07007499
MCF2L-AS1	0.9999999	0.031981026	0.070255432
ARRDC3	0.002748585	0.17657955	0.070303437
PRKD2	0.286120615	0.005667579	0.07031397
PROM1	0.355055247	0.008720917	0.070347302
HSD17B11	0.014911358	3.80913E-05	0.070398651
RAB1A	0.022912985	2.59581E-05	0.070401309
PAN3	0.00459453	0.324422449	0.07044493
MIB1	0.703670461	0.021346512	0.070502014
SDR16C5	0.098959986	0.000220748	0.07059844
TMEM82	0.9999999	0.032261002	0.070714519
LRFN4	0.031190011	0.899796097	0.070752214
TBRG4	0.969133627	0.039210644	0.070825797
AC108134.3	0.9999999	0.032419403	0.070973819
GPRC5A	0.000889498	9.49468E-09	0.071141867
DUOXA2	0.016979525	1.00491E-05	0.071204385
ARG2	0.007667098	0.225200933	0.071247203
RASA3	0.028059843	0.589272319	0.071487862
REXO1	0.008278461	0.506561732	0.071545042
STXBP3	0.576455046	0.014611696	0.071664442
SGPP2	0.057541419	0.000130011	0.071672105
HEATR1	0.003516351	0.182290383	0.072018924
C2orf72	0.9999999	0.033254627	0.072335968
API5	0.007481797	0.458026584	0.072433768
GPATCH2	0.006638571	0.414083895	0.072458307
ZNF620	0.236072962	0.002264888	0.072487223
EAF1	0.656212762	0.025453122	0.073046553
B3GNT9	0.9999999	0.033776945	0.073183525
HNF4G	0.121935869	0.00176159	0.073314722
DMWD	0.096274264	0.000648887	0.07336897
AC022966.1	0.9999999	0.033934868	0.073439148

UBOX5	0.9999999	0.033945975	0.073457115
ZNF737	0.9999999	0.034014875	0.073568541
KCTD11	0.758903408	0.03619148	0.073575698
DENR	0.000484117	0.099531805	0.073820427
RPS6KA2	0.000388156	0.056347604	0.0738287
CTSS	0.122294463	0.00129596	0.074238903
ANKRD11	0.001938581	0.18327702	0.074466997
CHMP7	0.212446991	0.002873563	0.07468147
GGA3	0.190859384	0.002336046	0.074876808
BICDL2	0.756703539	0.030959632	0.074902019
UTP14C	0.559540383	0.015253433	0.074904797
C17orf100	0.9999999	0.034881212	0.074964897
CCT3	0.02469813	0.605489226	0.074985977
MEAF6	0.619975969	0.014503228	0.075105652
CSRNP1	0.003541913	1.73986E-07	0.07514574
COASY	0.009056381	0.401027143	0.07515227
HEPH	0.9999999	0.03509165	0.075302787
MTSS1	0.006023415	0.349897859	0.07535273
GALNT3	0.036730086	0.000126268	0.075362574
CAPZA2	0.282053379	0.004544853	0.075450402
FAM43A	0.592678998	0.016232552	0.075450646
CCNL1	0.020664506	1.44653E-05	0.075468785
TFF1	0.489292713	0.010870208	0.07548259
TSN	0.229825183	0.001526529	0.075501697
RALB	0.536648395	0.014068764	0.075535792
SERTAD3	0.600327229	0.019348098	0.07583477
TUBD1	0.027212131	0.83953955	0.075850093
AC087741.1	0.042444659	0.833750565	0.075870363
TRA2A	0.017147332	0.510887474	0.075991248
TM2D2	0.040811776	0.000335201	0.076283523
ZFAS1	0.021660841	0.544480448	0.076368173
BTBD3	0.092622751	0.001562142	0.076433056
VNN3	0.633100548	0.018891617	0.076463866
IL15RA	0.010182163	0.000318791	0.076556604
C15orf53	0.042244822	0.415229678	0.076680339
FRMD5	0.924004987	0.046747817	0.076754233
BTBD1	0.283009535	0.009542949	0.076786733
IGHV3-7	0.9999999	0.036348815	0.077311144
EIF3I	0.035745615	0.741035019	0.077360099
TMEM163	0.690339622	0.035424848	0.077594839
HLA-DMA	0.308205189	0.004179211	0.077601246
LINC01348	0.9999999	0.036630464	0.077758734
C3	0.016289804	0.440071599	0.078109361

NME3	0.001236632	0.106507826	0.078128774
IL21R	0.038348832	0.775682398	0.07826181
ADCK1	0.9999999	0.037098252	0.078500276
S1PR4	0.010191506	0.018217567	0.078610652
TES	0.077933452	0.000515809	0.078668204
FGD6	0.312273897	0.008827368	0.078915885
LAMTOR5	0.501640203	0.018678524	0.078946965
NBPF1	0.411721277	0.010680599	0.079016458
MPST	0.266009161	0.008194338	0.079093791
PPP2R5D	0.020608478	0.660682556	0.07926063
TPGS2	0.044779366	0.814100611	0.079264827
NCR3LG1	0.9999999	0.037609903	0.079308735
DYNLT3	0.270599089	0.002809558	0.07935158
PI4KA	0.000268992	0.08042589	0.079355941
PLA2G15	0.306921325	0.009559034	0.079679335
SPG7	0.028175703	0.800913969	0.080046085
TMEM45B	0.118381121	0.001315418	0.080400599
TPST2	0.009926695	0.031509812	0.080551519
YEATS2	0.19154098	0.003478779	0.080603793
KIAA1191	0.18792094	0.003169221	0.080780402
PI3	0.002188242	0.001758163	0.0808803
GPRC5B	0.100700041	0.003171163	0.081597683
ARHGEF35	0.415628115	0.01095939	0.081769311
EIF4EBP2	0.567419455	0.025194984	0.081819062
SPINT2	0.084878356	0.00096874	0.081955697
MTHFR	0.122913839	0.002224353	0.081971799
LHFPL2	0.560278845	0.012087496	0.081979066
TTC31	0.002338211	0.199221839	0.082122231
VIM	0.220701391	0.026835247	0.082242518
SCAF8	0.065475081	0.000269504	0.082542922
LTBR	0.695066031	0.027296593	0.082617942
LINC00672	0.04892312	0.82739457	0.082643444
VAPA	0.009112373	0.275911737	0.082932298
ZBTB7B	0.129806143	0.002783657	0.082986061
ZNF45	0.018172515	0.727918745	0.083052934
ZNF623	0.040166861	0.00083297	0.083193101
NGLY1	0.007788273	0.299267491	0.083362212
PEX11A	0.239003352	0.008010202	0.083661216
GATA4	0.931688612	0.033783124	0.083663466
RNF128	0.270406378	0.006032221	0.083961833
IQGAP1	0.034032375	8.16728E-05	0.08398945
AC112220.4	0.001743403	0.208427999	0.084065364
RPS6KA5	0.547548964	0.027280778	0.084077193

C1orf226	0.836168078	0.035133421	0.084156992
AC100810.1	0.035216412	0.781562932	0.084175369
MAP4K3	0.330091612	0.007431183	0.084236936
GGCX	0.029606142	0.751472142	0.08432674
CD109	0.222313693	0.015354346	0.084429091
SCN9A	0.032772305	0.940515124	0.084513616
POF1B	0.077011832	8.6097E-05	0.084586398
ABHD4	0.040694933	0.835271627	0.084694919
MAGI2	0.9999999	0.041126191	0.084794497
ST3GAL1	0.007741108	0.269421365	0.085154232
RAD1	0.041311434	0.962766519	0.085365107
KMT2E	0.172481984	0.004181001	0.085400265
YARS	0.0089049	0.374363076	0.085430843
ATP6V1E1	0.077146351	0.001031771	0.085460341
SLC39A4	0.013881837	0.490541035	0.085472364
LRCH3	0.002586652	0.215282962	0.085626471
RWDD2B	0.015927847	0.636294099	0.085790767
HSDL1	0.427201357	0.019352633	0.085829363
ELAVL1	0.034566387	0.600393796	0.085964505
REL	0.013271802	0.000230363	0.086146951
BAG4	0.003346187	0.471789655	0.086196189
FAM98C	0.032083892	0.822712605	0.086229745
EFNB2	0.248842453	0.003602472	0.086253846
CLDN1	0.018652056	0.000182854	0.086602235
FKBP8	0.0284421	0.477662009	0.086717108
POLD4	0.036475562	0.819724679	0.086844725
RPS7	0.026634035	0.595445461	0.086934344
UGT1A1	0.9999999	0.04285829	0.08745433
RABGAP1L	0.148389366	0.002022401	0.087492683
PLEKHF2	0.145346406	0.004236898	0.087525651
ALPG	0.9999999	0.042984631	0.087647306
PRSS3	0.292818182	0.00130213	0.087691594
COBLL1	0.542308895	0.014882273	0.087816055
FGD2	0.037252587	0.9999999	0.087822817
ATP6AP1L	0.037426247	0.9999999	0.088114077
LINC01559	0.132085656	0.001679248	0.08824772
SSFA2	0.304587992	0.010588193	0.088317806
PLP2	0.020406929	0.595483992	0.088480721
WFS1	0.373633228	0.004165159	0.088562248
BACH1	0.006283209	5.33292E-05	0.088801171
H1FX	0.025788236	0.000720326	0.088898402
AL138724.1	0.012236725	0.225200933	0.088927725
AC007364.1	0.037925621	0.9999999	0.088949526

HOXB9	0.037992302	0.9999999	0.08906085
CELF1	0.048853459	0.677441209	0.089084835
PTDSS1	0.002480564	0.205967556	0.089114886
AGER	0.038097259	0.9999999	0.089235965
SGK1	0.001356937	0.149875554	0.089353804
C4BPA	0.016689038	0.52323258	0.089369655
SERPINB7	0.01805749	0.496354654	0.089775123
AL591845.1	0.038506571	0.9999999	0.089917594
NKTR	0.03922102	0.839241975	0.089925763
ZYG11B	0.346546642	0.014381093	0.09018456
NUDT6	0.038684164	0.9999999	0.090212711
NECTIN2	0.007702719	2.43836E-05	0.090292251
IMPAD1	0.002799982	0.184711141	0.090399053
CD200R1	0.9999999	0.044988918	0.090690576
TTLL10	0.038981691	0.9999999	0.090706283
MLKL	0.378643595	0.009240251	0.090743576
RHBDF1	0.317766525	0.004464401	0.090762739
CLASP2	0.027568429	0.613554899	0.090795568
F11R	0.010737226	1.44864E-05	0.091047058
GTSF1	0.039194583	0.9999999	0.091058807
RNF213	0.019001119	0.300968564	0.091098192
COP1	0.044335532	0.808323879	0.091458867
ETNK1	0.127685034	0.002396223	0.091501448
AC002467.1	0.239003352	0.016107752	0.091575654
BANK1	0.466770231	0.012788173	0.091672959
CMPK1	0.13653994	0.001645726	0.091935669
PIK3CB	0.016735805	0.617014746	0.092009454
AL357060.1	0.039856447	0.9999999	0.092151386
AP003068.2	0.626178611	0.020041086	0.092474832
KLHL25	0.239003352	0.015003937	0.092577806
KIF24	0.04017685	0.9999999	0.092678473
TMEM243	0.008690407	0.22168199	0.092680789
EIF3F	0.046458134	0.652400255	0.092985282
CLTCL1	0.040377202	0.9999999	0.093007473
CD99L2	0.040389904	0.9999999	0.093028316
AC011676.2	0.040520029	0.9999999	0.093241732
THOC2	0.001421771	0.06815864	0.093343625
GTF2IRD1	0.906988839	0.04808432	0.093537812
MS4A8	0.9999999	0.046909419	0.093575873
CMTM7	0.048281555	0.80590789	0.093620783
ZNF529-AS1	0.040903956	0.9999999	0.093870298
ADAT3	0.041097208	0.9999999	0.094186068
E4F1	0.30956315	0.023776182	0.094379442

LPCAT4	0.206981936	0.002620772	0.094469455
APOBR	0.041270928	0.9999999	0.094469571
ZNF627	0.021554786	0.67025996	0.094492421
PLEKHS1	0.194203462	0.006216485	0.094531181
ZNF182	0.041319583	0.9999999	0.094548913
NAA20	0.003570084	0.171200689	0.094695075
TRMT6	0.008605706	0.355646426	0.094793766
ABCA5	0.029495256	0.637825175	0.095008072
NR2F6	0.163847153	0.001700443	0.095054037
CDR2L	0.787885645	0.039948454	0.095183974
ZNF638	0.000763049	0.103831174	0.095243016
MT-ND3	0.004223809	0.14131921	0.095376129
FAM220A	0.745112122	0.022365833	0.095421984
DCAF8	0.005801652	0.258079683	0.095467086
TMC4	0.003486897	0.283015252	0.095520987
UBE2D1	0.279581401	0.008808337	0.095652934
HDAC5	0.258806641	0.001001904	0.09567454
AC093525.4	0.042021086	0.9999999	0.095690003
PNPLA2	0.050773298	7.29941E-05	0.095763939
GTF3C2	0.007154749	0.000268743	0.096135624
VTN	0.9999999	0.048671582	0.096198185
AXL	0.042455628	0.9999999	0.096394184
TGM2	0.002667329	0.216423121	0.09668896
GSPT1	0.008212047	0.301055041	0.096874826
VGLL4	0.001427962	2.83113E-06	0.097042558
HDGFL2	0.203806007	0.004764006	0.0970855
SLC25A37	0.013182032	0.312043116	0.097215167
PRRG1	0.695392792	0.043527955	0.097290834
SLC22A23	0.747714347	0.044133562	0.097549232
NIFK-AS1	0.239003352	0.017679096	0.097676736
AMOTL2	0.495339159	0.024864636	0.097721077
RAB3IP	0.017091409	0.000288033	0.097818692
CDKN1B	0.613851982	0.024350719	0.097841821
SERGEF	0.021213221	0.640337558	0.098069024
VAMP8	0.207282518	0.003349607	0.098072978
MICAL2	0.207327435	0.005133468	0.098488227
HECTD2	0.043791701	0.9999999	0.098546889
CAVIN1	0.043859095	0.9999999	0.098654986
ZNF503	0.190422185	0.008583827	0.098683026
LMBRD2	0.014617928	0.636883324	0.098851461
GPSM3	0.028028217	0.608193855	0.098912778
ZFP14	0.005365454	0.306204085	0.098974273
FBXW11	0.073923013	0.001417007	0.099240833

AC234772.3	0.044296653	0.9999999	0.099355707
PRSS22	0.007258674	0.273976937	0.09938395
MITF	0.041672251	0.806865555	0.099510429
PARP14	3.08015E-07	0.001813067	0.09952345
KIAA0040	0.350185917	0.014826219	0.099875422
NNT-AS1	0.359852074	0.007126694	0.099956377
PRKD3	0.003995741	0.0177051	0.100033579
TMEM106C	0.003152372	0.106277117	0.100049685
TRANK1	0.342122415	0.02029155	0.100254649
B3GALT5-AS1	0.095787987	0.00169483	0.100371771
PDLIM7	0.006625187	0.360143733	0.100507234
AC002401.4	0.045026076	0.9999999	0.100519575
PIWIL4	0.827054533	0.0408961	0.10080025
TMEM131L	0.032481684	0.695444399	0.100877036
TBC1D10A	0.004158846	0.26356951	0.100922418
RTL8A	0.552019792	0.020478076	0.101059008
AC142472.1	0.045433889	0.9999999	0.101168002
PACRGL	0.007440798	0.225200933	0.101169859
AC005726.1	0.045436238	0.9999999	0.101171732
OSBPL11	0.027420803	0.008341368	0.101457071
RUSC1	0.00247344	0.129931355	0.101467865
RALA	0.201672465	0.002838305	0.101485988
SH3KBP1	0.35587399	0.02071904	0.101561665
AC010618.3	0.045687791	0.9999999	0.101570894
EDEM3	0.02938559	0.000262063	0.101788415
SNRPG	0.030211311	0.585981498	0.101910101
NDUFA13	0.001845074	0.089353458	0.102193833
C15orf61	0.795010082	0.046287975	0.102218129
LRP5	0.244574934	0.006412741	0.10229073
EHF	0.364530509	0.006842473	0.102515834
DAAM1	0.368976346	0.010211809	0.102550496
NFIX	0.011839987	0.462175754	0.102774322
BMP1	0.006033151	0.273539956	0.102859272
CCDC51	0.623977922	0.032321042	0.103337192
SLC9A7	0.000506633	0.09454082	0.103449481
TWF1	0.085512781	0.000519207	0.103722099
TPM3	0.010486691	1.41047E-06	0.104022243
CDC34	0.08864721	0.001121204	0.104257745
LINC002481	0.047470581	0.9999999	0.104382614
ATP2B1	0.084135907	0.000649078	0.104850832
CCDC88A	0.028783731	0.528582889	0.104861459
IGSF9	0.180925379	0.018022503	0.105054423
UVSSA	0.23444281	0.006106991	0.105613056

ZNF83	0.634559918	0.010577161	0.105636205
RPS19	0.024763751	0.500419306	0.105651863
NEIL3	0.048315344	0.9999999	0.105704692
TMC5	0.110150272	0.00133758	0.105847243
ALKBH3	0.074610073	0.000333772	0.105906101
ATG16L1	0.002426574	0.022841995	0.106123885
FOSL1	0.36513644	0.009231029	0.106165933
RAP1A	0.005199714	0.209425208	0.106413796
GOLPH3	0.367427722	0.012566286	0.106551372
NDUFA3	0.006703148	0.253643695	0.10723043
PLPP6	0.032830541	0.001404806	0.107493079
RXRB	0.896454873	0.041336099	0.10751762
MIR4435-2HG	0.007151714	0.11509904	0.10770704
GMFG	0.040946699	0.889001287	0.108722405
GNA11	0.368757599	0.014946594	0.108752865
ITM2A	0.048957669	0.921411863	0.109043767
ADAM8	0.001260175	0.064753143	0.109190214
NRM	0.034104336	0.407975213	0.109195861
KAT6A	0.304919043	0.008769949	0.109274337
COA3	0.446756621	0.035331944	0.109563722
DGKH	0.006041116	0.187759127	0.109634444
TNFSF12	0.010615647	0.086566659	0.109909634
GPBP1L1	0.103629581	0.000465679	0.110026051
CIITA	0.373614967	0.008226526	0.110425061
AMN	0.136860221	0.001474161	0.110489591
GBA2	0.586120144	0.029727616	0.110589465
TCTA	0.079198797	0.000799168	0.110711818
SDCBP2	0.191773492	0.003712734	0.11093616
RDX	0.111565375	0.015395675	0.11110729
GPR137B	0.213889322	0.003358943	0.111659936
STAU1	0.1828067	0.004596604	0.111694082
CAAP1	0.43307063	0.028971303	0.112062462
SH2D1B	0.093675586	0.002264595	0.11226462
FARP1	0.134556197	0.001213795	0.112274917
SELENOP	0.25714906	0.003574292	0.112291552
BIRC5	0.922249558	0.034148415	0.112421652
CEP290	0.047635007	0.000463752	0.112883975
HELZ	0.037046115	0.000154959	0.113168745
MIER3	0.168070959	0.002091041	0.113255335
MIR194-2HG	0.385443772	0.012531385	0.113301188
GNG12	0.318938407	0.009667889	0.113371039
EYA3	0.00152049	0.022264397	0.113383378
WBP2	0.16709168	0.006199963	0.11364224

PRDM16	0.786735611	0.043462276	0.113689518
BABAM1	0.009126919	0.222218195	0.113718941
DENND4C	0.01173655	0.323722783	0.11385639
ZNF629	0.727436453	0.025277427	0.113930903
GALNT7	0.234005819	0.004022974	0.113945976
HINT1	0.033227984	0.55615609	0.113946869
ALDOC	0.015287563	0.223783178	0.114078507
SMIM8	0.034860403	0.660284598	0.114141173
TOX4	0.002308773	0.163919882	0.114279559
NR2C2AP	0.012143521	0.33130464	0.114489512
CENPT	0.020741654	0.553277368	0.114835481
CST3	0.004039012	1.43844E-05	0.114845783
TMPRSS2	0.00845695	1.39734E-06	0.114925064
DHPS	0.007371466	0.188547629	0.115084647
PYCARD-AS1	0.239003352	0.035762154	0.115726223
PIGK	0.00164634	0.11174484	0.115755055
CIPC	0.030798744	0.703665207	0.115808402
RNF113A	0.145095116	0.00305416	0.116343808
ARPC3	0.004925731	0.113834132	0.116400802
CTPS1	0.008048906	0.175394743	0.116437562
ATP5MC2	0.018639301	0.347887232	0.116504996
IRX5	0.529251692	0.036574175	0.11652083
DTD2	0.239003352	0.012249635	0.116620655
FAM210B	0.021836274	0.000192838	0.116714966
CA13	0.013345727	7.27247E-05	0.116758116
QSOX1	0.112980603	0.001275075	0.116798737
PBXIP1	0.022374954	0.000101493	0.116810935
MAP3K1	0.13200672	0.00497148	0.117294958
SLC25A24	0.105438389	0.00245494	0.11737545
HIST1H4C	0.179776864	0.000182807	0.117431206
AKR1B1	0.02533811	0.407147208	0.117496383
LAPTM5	0.778654077	0.030929714	0.117666525
LAMTOR3	0.343006156	0.012295713	0.117708492
ZNF185	0.049951367	0.804153936	0.117860344
CPT1A	0.563849578	0.009947096	0.117922995
NDUFB3	0.043862374	0.555188531	0.118074578
HSD17B2	0.128692886	0.000660976	0.118192472
PTTG1IP	0.000143027	1.15987E-08	0.118194858
ITGA3	0.018346605	0.326574065	0.118592277
MCRIP2	0.021382842	0.000409654	0.118607865
NFIB	0.42570082	0.009932807	0.118927738
YES1	0.251272094	0.00679625	0.118997622
MBIP	0.025242304	0.000251434	0.118999238

MEIS1	0.055068265	0.004123659	0.119007827
RAMP1	0.224396057	0.003879404	0.119146769
PAFAH1B2	0.285618596	0.005523454	0.119186029
SEC11A	0.020204591	0.303596451	0.119308249
TFG	0.325902256	0.012231527	0.119518334
PLA2G10	0.813461294	0.041432501	0.119574257
GATA6	0.02582565	0.000162293	0.119973751
GADD45GIP1	0.016355989	0.201391764	0.120117172
EXOC3L4	0.051318786	0.000484903	0.120179549
KHDC4	0.003724817	0.16015753	0.120419155
FRAT2	0.269976024	0.008350737	0.120586266
ZNF761	0.01940105	0.001856445	0.120587808
COPS4	0.10082235	0.001114622	0.120595431
JUN	0.021906407	5.48507E-05	0.120877971
CYBA	0.003822481	0.138093186	0.121013393
UBD	0.643374347	0.038287406	0.121123996
POLR1A	0.120760345	0.009272672	0.12113508
PAPSS1	0.61368569	0.040028687	0.121544171
MTIF3	0.504577801	0.047787367	0.12160462
INTS6L	0.020725567	0.225200933	0.122068048
CCL2	0.065249026	0.011196797	0.122204716
AC027644.3	0.404260745	0.020821157	0.12222426
TIGD1	0.033611188	0.225200933	0.12228042
IGBP1	0.000892466	0.041826818	0.122325444
ZNF669	0.003435452	0.357653206	0.122487336
CCDC97	0.000406844	0.089972238	0.123065639
MT-CO3	0.002585654	0.092443609	0.123115221
ZBTB43	0.142556816	0.00631654	0.123129223
PAWR	0.413977708	0.041209045	0.123311195
TERF1	0.314077042	0.006474823	0.123419571
STK35	0.01474089	0.608602528	0.123538961
PDLIM5	0.012209876	4.50519E-05	0.123859881
IKBKB	0.030166432	0.578062714	0.124144615
BCAM	0.005709561	0.180651042	0.124151268
MAOB	0.101954764	0.007628572	0.124227884
TMEM238L	0.293606487	0.006020408	0.124398717
UBXN2A	0.479816114	0.023198821	0.124489024
KPNA3	2.66606E-05	0.000357331	0.124618987
AC116366.1	0.016073459	0.008917479	0.124778986
C2CD2L	0.266135202	0.013689736	0.124804416
SUB1	0.037727626	0.466769346	0.124845146
PIM3	0.016294392	3.23226E-05	0.125153774
NFE2L1	0.031257689	0.462454991	0.12517842

SELENOS	0.193300184	0.004388862	0.125225193
TM9SF2	0.269857035	0.015184811	0.125420133
TNFRSF12A	0.063363276	0.000241419	0.126151612
MPDU1	0.011518137	0.322272876	0.126434672
DOPEY2	0.655573366	0.042032365	0.126777053
C11orf54	0.010235891	0.000207378	0.126986419
KIF9	0.000839513	0.099842	0.127002501
AGA	0.004129075	0.274904321	0.127377914
C17orf75	0.00352637	0.019783155	0.127514679
YDJC	0.007681355	0.213188778	0.127599312
TMEM92	0.023755996	0.480805987	0.127961928
CLIP4	0.002199677	0.130390902	0.128099452
CDH1	0.071264812	0.000404812	0.128127858
YIPF4	0.115588005	0.004306176	0.128343143
HSCB	0.000241035	0.019252629	0.128425229
CAPNS1	0.002269209	0.124368832	0.128927253
CES2	0.34278444	0.020577362	0.12904637
MDM4	0.019081666	0.315886829	0.129054994
TM9SF3	0.330882916	0.032241151	0.129079998
DYNC1H1	0.01106182	0.262163116	0.129239576
SESN3	0.001569047	0.083987526	0.129245268
KLK12	0.040260609	0.225200933	0.129248155
ILKAP	0.074322798	0.002254274	0.129385362
ZNF24	0.02245763	0.000320111	0.129392263
DNASE1	0.037603626	0.364083752	0.129403522
ZMIZ1	0.371462387	0.016303289	0.129576202
C5orf30	0.207907522	0.004301006	0.129681151
TMEM60	0.107892753	0.005720456	0.129725257
TMEM171	0.00131231	6.29079E-06	0.129781864
DRAM1	0.002587026	0.061378806	0.130292283
ANXA4	0.283288625	0.01540349	0.13032158
SELENOT	0.019182393	0.315517074	0.13074747
NEBL	0.403367391	0.019006191	0.130830056
AP1S1	0.423660791	0.025230021	0.130974644
KIAA0232	0.001042502	0.041548524	0.130991959
FGD4	0.052590681	0.002439255	0.131003147
BHLHE40	0.001178722	0.116807395	0.131063082
PSMC6	0.000150486	0.024957847	0.131426358
NAPA	0.186580578	0.002187272	0.13158988
ZNF706	0.095203243	0.002461322	0.131598159
DCXR	0.04072587	0.486422677	0.131786007
TMEM217	0.026857416	0.225200933	0.132268757
LAP3	0.454073758	0.023349784	0.13254057

IL18BP	0.162993138	0.003928993	0.13276493
SGMS1	0.001191815	0.043182498	0.13325662
AC009414.2	0.645886669	0.04243813	0.1335487
PTS	0.46728625	0.032412515	0.133772864
WWC3	0.028444499	0.493796792	0.13408876
UCK1	0.000713245	0.018921342	0.134808671
EPOR	0.010682847	0.225200933	0.134913342
HDAC9	0.191852468	0.004662402	0.135045238
PLEKHG2	0.022788369	0.410509718	0.135181262
MALT1	0.001387483	0.000393665	0.135281879
CAVIN4	0.642292229	0.033638898	0.135372207
AURKAIP1	0.012848034	0.189981166	0.135545923
JOSD2	0.283339119	0.008769189	0.13572302
PQLC1	0.011645459	0.363187373	0.135824291
WWTR1	0.055341174	0.001693929	0.135962812
ADPGK	0.037817492	0.657952434	0.136249542
ZNF266	0.140541284	0.010482223	0.136399603
PIK3C2B	0.00436825	0.092109809	0.136601705
TRAPPC12	0.031118423	0.557845837	0.136671035
MT-ATP6	0.003764282	0.064641362	0.137394308
RTP4	0.359843387	0.011023476	0.137400945
ANO6	0.014760031	0.386700635	0.137431186
CTTNBP2NL	0.309164001	0.030350139	0.1375198
MYORG	0.023094835	0.00099075	0.137562233
CARD14	0.22689318	0.009670331	0.138177079
ATP6V1H	0.120838426	0.0035465	0.138318024
EPS8L3	0.13476152	0.002094874	0.138416049
MFGE8	0.001063422	0.000226285	0.138571194
TMEM176B	0.55839263	0.047098511	0.138767707
SOX9	0.037021004	2.81852E-05	0.139057041
SPHK2	0.032116015	0.565049279	0.139181495
TPBG	0.036459866	0.000466407	0.139266067
RPS15	0.005474562	0.090524866	0.139771174
RNF123	0.22235829	0.008528153	0.139925189
ABCA10	0.04904322	0.649769366	0.140037834
PRICKLE2	0.015888196	0.290898904	0.140263102
CYSTM1	0.477012165	0.039357545	0.140277655
EFNA1	0.185224913	0.008497784	0.140295091
AC099518.5	0.02257469	0.225200933	0.140466528
ANKRD39	0.006284085	0.105165282	0.140622917
KIF13A	0.583957554	0.042132955	0.141097125
TMEM41A	0.541698629	0.035230504	0.141105896
AL139393.2	0.019733829	0.225200933	0.141236439

EMP2	0.113196575	0.000147909	0.141677611
LEMD1	0.534907922	0.018047787	0.141810043
SLC15A1	0.040613957	0.587687779	0.142067394
TRIM29	0.155041478	0.00479013	0.142745276
MFSD6	0.154509625	0.004213679	0.14283553
PCBD1	0.016501512	0.345579292	0.14286383
UBE2L6	0.451371064	0.017393702	0.143292212
CDC14B	0.305698006	0.018379079	0.143412092
LYN	0.135347064	0.003443731	0.143550132
AL118506.1	0.011335312	0.087772526	0.143740003
PDGFB	0.018211086	0.225200933	0.144139981
ITGB8	0.164843039	0.002972791	0.144170365
WDR4	0.016790022	0.40341115	0.144215061
CSAD	0.036263172	0.607380903	0.144351784
H2AFJ	0.425384109	0.02714288	0.14478551
SMCHD1	0.214767136	0.011874815	0.14486951
GNB1	0.222375753	0.005496811	0.144893837
ANAPC7	0.016109837	0.443097889	0.14556636
EPN2	0.093775134	0.002178313	0.145580783
AAMP	0.185739245	0.00549708	0.145715531
GNL3	0.026484231	0.315384339	0.145786967
SNX14	0.003197372	0.031788325	0.145888233
MAD2L2	0.027795071	0.509785253	0.145959428
AARSD1	0.019561512	0.225200933	0.146119503
STS	0.66086763	0.044569027	0.146263328
IFIT1	0.025857622	0.059349247	0.146289935
PRDX1	0.016106436	0.196188065	0.146782661
RTN3	0.034060961	0.45753586	0.146877121
SETD4	0.006327487	0.06746273	0.147130818
UBA6	0.039270464	0.47445248	0.147359347
MFHAS1	0.075997034	0.00205978	0.147440743
TNIP1	0.020371915	9.39481E-05	0.147575542
ABL2	0.009481582	0.095878721	0.147703172
YPEL3	0.097646488	0.005100477	0.148025287
RCN2	0.323627325	0.012019206	0.148697333
GATAD1	0.289451936	0.020142286	0.148765537
BPHL	0.182729764	0.008683789	0.148974373
GTF2H2C	0.011404958	0.278879028	0.149114144
NIPSNAP1	0.000138282	0.015647495	0.149312488
PAK1	0.360550756	0.025177697	0.149350591
STX8	0.003578867	0.217773748	0.149384171
HNMT	0.380721643	0.008280346	0.149574918
TMEM115	0.245208017	0.017857447	0.149635381

SENP2	0.517652915	0.030143226	0.149694705
GPATCH3	0.036400385	0.394988768	0.149741942
SLC10A3	0.001427299	0.051349133	0.14976588
TAPT1	0.338356835	0.024665134	0.149780374
BEAN1	0.043669449	0.225200933	0.149910207
PPTC7	0.000699754	0.023807279	0.150090928
HK2	0.168621839	0.003448007	0.150433889
NR3C2	0.427739445	0.020017512	0.150614151
BCL10	0.002465297	0.000138634	0.150614622
RAB11FIP5	0.371519812	0.008554067	0.151126803
POLR2J3.1	0.005577889	0.153423083	0.151187079
NFKB2	0.472666432	0.040767477	0.151448199
DNAJA2	0.154377408	0.008813731	0.151810624
IFI16	9.76419E-05	0.008165577	0.152106902
RAB9A	0.379456204	0.015066454	0.152113596
PPP1R15A	0.000653888	9.47993E-07	0.15213648
ARF6	0.399170989	0.042660825	0.152466871
AASDHPPT	0.196123375	0.010551313	0.152798436
TIPARP	2.51539E-06	1.54237E-07	0.153144821
MCPH1	0.007575957	0.266799019	0.153456419
MOB3B	0.023207322	0.000160984	0.153555263
NGRN	0.499683029	0.037338648	0.153708915
TAF8	0.042531927	0.663000699	0.153856952
MDM2	0.014610693	0.236247786	0.153881717
CALM2	0.372210802	0.027445848	0.15398146
ICA1	0.434201246	0.029741841	0.154078488
LRRC75B	0.132153278	0.005282559	0.154194125
WDR6	0.008279047	0.455462763	0.154300028
PGP	0.021937705	0.426480913	0.154402993
PLEKHA6	0.000256071	0.004030198	0.154452733
ICAM1	0.025482159	0.003018715	0.154733834
RANBP6	0.001516018	0.041316948	0.155012493
NAB1	0.035414762	0.001061463	0.1551798
SHPRH	0.003750364	0.063378007	0.155784733
ADAM10	0.025549393	0.000509966	0.155792589
TUBB6	0.047140188	0.564446663	0.156665802
SIGIRR	0.170215988	0.008605875	0.156962457
PUS7L	0.000926283	0.023676709	0.15750215
PINX1.1	0.040955249	0.301796933	0.157693052
EPS8L1	0.102076666	0.00108807	0.157753854
ANXA1	0.060346983	0.000821344	0.158001757
GEMIN5	0.239003352	0.040054648	0.158184905
TPP2	0.002307009	0.116463935	0.158260898

ENC1	0.025076239	0.350669487	0.158367745
MAPK1	0.01490174	0.000387937	0.158459199
RBX1	0.035325518	0.40301988	0.158568201
AP001107.9	0.451075582	0.028712991	0.158930628
TBC1D1	0.421239196	0.018915889	0.158969253
LRP10	0.044443796	0.000129207	0.159042153
PHLDA1	0.001329035	0.045247748	0.15934341
CKAP4	0.068243523	9.32711E-05	0.159360239
SPATA5	0.026317719	0.572625862	0.159417916
COPS2	0.063596635	0.001674534	0.159769041
SERPINB6	0.000164934	0.001190889	0.159860968
MUL1	0.407861884	0.019104892	0.159969218
ZMYM2	0.015582021	0.237813353	0.160089079
TSC22D3	0.023621615	4.49368E-06	0.160478233
KLHL7	0.021566782	0.498049862	0.161186403
DSP	0.117429135	0.001298291	0.161472581
SMYD3	0.320701814	0.022923264	0.161648677
STAM2	0.341249685	0.01542771	0.161710366
HOMER1	0.006970052	0.16179092	0.161744348
SYS1	0.009242679	0.215200882	0.161804351
FANCA	0.029183798	0.657724528	0.161933129
EFCAB11	0.022185742	0.405671311	0.161974966
ZNF615	0.097624692	0.011335791	0.162032205
CDC42BPB	0.000218994	0.013656271	0.162494956
PRSS21	0.040897215	0.225200933	0.162505078
TRMT5	0.380936116	0.012393154	0.162751813
DSG2	0.163381857	0.009115579	0.162918522
MTAP	0.346808543	0.022975478	0.162965767
TATDN1	0.033926302	0.4397612	0.163024873
FOXC1	0.22836799	0.010030674	0.163257393
MARCH6	0.007524771	0.333293839	0.163389978
CDK5RAP1	0.00899769	4.34509E-05	0.163620722
HRAS	0.02916318	0.404188594	0.163733043
KIF23	0.041001682	0.08294518	0.163822869
GPATCH8	0.000787757	0.02519381	0.164040148
COBL	0.100049639	0.004660049	0.164099415
STK40	0.041729397	0.001095776	0.164173838
RIOK1	0.026186795	0.423976501	0.164643691
BNIP2	0.026751054	0.002815701	0.164751185
AHCYL2	0.026799745	0.00017853	0.164801343
IRF1	0.011757616	0.00026592	0.164939006
PAM	0.037441145	0.397847056	0.165270941
STX2	0.002649082	0.010700634	0.165559392

CHMP4B	0.001299089	4.946E-06	0.165739538
PRKCSH	0.001277952	0.020495342	0.166243612
KRT8	0.083603651	0.001032843	0.166298738
EZH1	0.001074434	0.059337744	0.166451502
IFI27	0.017540889	7.15367E-06	0.166526811
APBB2	0.011199525	0.147192704	0.167210531
JAG1	0.330017153	0.010127576	0.167382144
CHFR	0.02565621	0.5440874	0.167778124
BCAP31	0.018251521	0.271993322	0.167795374
RSBN1L	0.034215237	0.40370389	0.168087187
ADPRM	0.323623526	0.005724601	0.168301084
UQCRH	0.023258195	0.311704085	0.16838762
PTPN11	0.022478051	0.43744929	0.168465799
MAP2	0.028526871	0.225200933	0.168476208
TET2	0.083350767	0.001063288	0.168585888
TMEM9B	0.070994341	0.002317516	0.169081487
NADK2	0.013989672	0.292977057	0.169208166
EXOC5	0.439223491	0.03057205	0.169344076
GOLPH3L	0.365082785	0.022116552	0.169761503
INTS5	0.010712589	0.239692816	0.169914315
TNFSF10	0.08467519	0.001983091	0.170436889
PFDN6	0.040649442	0.519657717	0.170626706
PARM1	0.118020718	0.001847358	0.170654996
GPRC5C	0.380978831	0.028458972	0.171051994
RASSF6	0.150236774	0.002576931	0.171310509
ADD1	0.02619588	0.000299244	0.171650539
SYF2	0.11849485	0.001242986	0.17200133
BRD8	0.168895237	0.008747722	0.172049775
CDKN1C	0.050046021	0.000393081	0.172168222
UPP1	0.020577945	0.252535769	0.17221823
ATF6B	0.209560824	0.013020303	0.172397916
ZFP36	0.001126762	2.81422E-06	0.172445336
AC027307.2	0.239003352	0.04449859	0.172583916
CCDC32	0.218243388	0.008654111	0.172786576
RPL13	0.033993876	0.488854655	0.172843338
BAG5	0.206022312	0.013937722	0.173305682
DNAJB6	0.111020238	0.007971473	0.173528128
ERP29	0.00384179	0.070532642	0.173797544
KLF4	0.003058645	4.87275E-09	0.173806139
LPAR5	0.239003352	0.031909542	0.174146235
IST1	0.005119787	8.18847E-05	0.17419694
STK17A	0.00334297	0.030318588	0.174575799
PLEKHA2	0.047038032	0.003115564	0.17485659

RPL18	0.030477789	0.370481856	0.175262764
DUOXA1	0.061681052	0.002305319	0.175335132
POMGNT1	0.668065674	0.042957924	0.17593324
SH3GL1	0.337978495	0.020899138	0.176190231
A4GALT	0.229341762	0.006308994	0.176430922
FAM76B	0.002412534	0.029261662	0.176510034
GRIP2	0.239003352	0.038589185	0.176582329
RAB30-AS1	0.009482674	0.191346066	0.176682992
NPNT	0.415625306	0.002788386	0.176738136
PRELID1	0.020941784	0.196765818	0.176937058
RPL39	0.047687277	0.527344258	0.177123635
INTS12	0.007854512	0.119509621	0.177126023
RAPGEF2	0.032247697	0.000606407	0.177231315
AC137932.3	0.239003352	0.013312919	0.177296844
ERBIN	0.037181709	0.000857304	0.177848207
RBM12	0.143020927	0.006519606	0.177887224
SCAF11	0.015861131	0.000212361	0.178290992
HACE1	0.021714303	0.225200933	0.178380869
MARCKSL1	0.082885987	0.001486141	0.178384529
CEBPG	0.463074184	0.044653208	0.178501055
LAGE3	0.017233132	0.274630212	0.179289858
STT3B	0.028686718	0.473507957	0.179727488
KCTD13	0.051940047	0.002710098	0.179967994
TOMM70	0.514884006	0.038847243	0.180104068
HLA-DRA	0.222893339	0.005708096	0.180272411
EIF3J-DT	0.153028483	0.004924931	0.180569902
MGAT4B	0.192126546	0.010152676	0.181389756
S100A14	0.142661364	0.001541332	0.181465205
ZFP36L1	0.00486719	0.000373558	0.181481752
MTUS1	0.150003248	0.002910994	0.18184503
YTHDF2	0.0674038	0.002076985	0.182384816
MCL1	0.074016948	0.001863187	0.183286555
RND3	0.038105911	0.001260828	0.183401999
MYLIP	0.240245015	0.017075992	0.1835055
TRERF1	0.233718296	0.01277971	0.183607743
FAM83H	0.580658122	0.048682236	0.183776018
PITX1	0.150635211	0.007366265	0.184278482
HLA-A	0.030868893	0.000797369	0.184426877
KPNA5	0.481121791	0.04213007	0.184451091
NEK1	0.035538426	0.207944948	0.184798007
H6PD	0.021994395	0.233730996	0.18521509
GMDS	0.294941717	0.017396441	0.185650372
FBXW4	0.24487201	0.01327128	0.185890869

LAIR2	0.530147954	0.040974846	0.186280511
SFPQ	0.038359088	0.425293608	0.186771136
SEC62	0.000307297	0.006085601	0.186856079
PPM1M	0.019120671	0.094516284	0.186913543
TRAF5	0.012601101	0.008385906	0.18696484
PUM3	0.016948236	0.168026646	0.187013112
ZNF431	0.014750072	0.156764677	0.187089909
TMEM8A	0.085567454	0.003793482	0.187125906
GGT6	0.021710744	0.225200933	0.187367864
PCNP	0.128013874	0.005947528	0.187431562
TCEAL4	0.469059601	0.044072053	0.187722607
ABT1	0.014288738	0.202017556	0.187850239
MT-CYB	0.000685812	0.00911338	0.188093721
SLC2A1	0.009933323	0.126693105	0.18811083
PLXNA2	0.185680717	0.014237277	0.188123328
PJA1	0.001665556	0.143925119	0.188144649
DUSP10	0.00031253	0.000872618	0.188192526
DR1	0.187402127	0.015520584	0.188875221
TMEM176A	0.226355567	0.012591968	0.189027872
TMEM43	0.000464218	0.050329398	0.189098989
FUT3	0.004872648	2.70905E-05	0.189199125
MOSPD1	0.023792785	0.004740824	0.189337954
SLC40A1	0.537483358	0.04553334	0.189370885
AP002807.1	0.481530784	0.035208026	0.189413511
FAM160B1	0.404596097	0.032125949	0.189588242
PURA	0.017473994	0.27830518	0.190081844
ZDHHC18	0.123498584	0.003386551	0.190149091
METTL2A	0.001803664	0.026290759	0.190722566
AFAP1	0.149626152	0.007641581	0.190809364
PPFIA1	0.158333956	0.007331219	0.190895199
TTC7A	0.014255911	4.65343E-05	0.190923233
TM4SF1	0.202059628	0.004274576	0.191041755
PHF13	0.019680438	0.261570569	0.19131947
OASL	0.041666771	0.027054711	0.191366838
BTC	0.176449147	0.006954189	0.191426455
ZNF316	0.017358604	0.335239406	0.191588593
CTDP1	0.023366534	0.436936554	0.191611305
GSE1	0.041601942	0.423117008	0.1917347
ZNF107	0.000892316	0.027379301	0.191935259
TAF3	0.021313732	0.306139577	0.191940475
C19orf18	0.042273625	0.515406916	0.191955064
GTF2F2	0.30687254	0.047085399	0.192054021
MX2	0.012114143	0.000980131	0.192070257

NOSIP	0.013993721	0.193313829	0.192091305
TRIT1	0.239003352	0.033473084	0.192120143
PRELID3B	0.039412611	0.001381926	0.192193371
STAP2	0.009780008	0.00021712	0.192313572
KLHL17	0.376780146	0.043360841	0.192417947
ACOX3	0.006041473	0.037433948	0.19245812
C6orf106	0.043005449	0.38683799	0.192547298
EGLN1	0.001871448	0.047765843	0.19261044
AL078604.2	0.239003352	0.034021304	0.192648088
PSMA3-AS1	0.0055659	0.149683753	0.192656483
DNTTIP2	0.2458969	0.032984002	0.19286074
CDKN2A	0.009509567	0.00033953	0.192954574
NEMF	0.015058075	0.22282675	0.192981583
FOXM1	0.015226088	0.027787811	0.193430771
FBXL8	0.015931945	0.051272499	0.193501456
CPLANE1	0.032612323	0.083178769	0.193556041
SS18L1	0.093994507	0.003860843	0.193561101
TMPRSS4	0.000430502	0.003404829	0.194352195
VDAC2	0.149879431	0.008169329	0.194655253
G3BP2	0.026707015	0.324728395	0.194755534
AC044849.1	0.042369235	0.399177467	0.195130792
PLIN3	0.129169122	0.002911537	0.195146875
MBP	0.073852443	0.001075061	0.1953241
ISCA1	0.001285806	0.008343457	0.195347821
DOCK11	0.014769231	0.166334066	0.195454774
TGDS	0.038604586	0.642243196	0.195518152
ATG12	0.030093127	0.360244057	0.195534664
SEPT8	0.314492283	0.016505536	0.195572138
TOB2	0.133276508	0.004587062	0.196138405
ANKRD27	0.239003352	0.039875723	0.196269686
ZDHHC14	0.073970331	0.000146611	0.196575493
NEK11	0.023056589	0.25833308	0.196603169
CEP162	0.240291829	0.008893579	0.196838342
CNN2	0.002698226	0.035539348	0.196850219
ATXN7L3	0.31364594	0.032022646	0.197177892
RHOF	0.015444054	6.76287E-05	0.197745451
STAMBP	0.154166291	0.010280086	0.198107799
DDX58	0.009645327	0.260759594	0.198199816
FAM83E	0.277940389	0.016929112	0.198546812
MED24	0.034970457	0.437447866	0.198715413
ALG5	0.036384116	0.319860781	0.198848332
USP47	0.154595932	0.002182737	0.198886562
COX6A1	0.001325264	0.031772377	0.198968546

WDHD1	0.093213351	0.03993127	0.199029187
HSD17B8	0.023472821	0.322670244	0.199090754
B3GNT5	0.084415096	0.003871852	0.199380715
PDP2	0.105186314	0.008963422	0.199654029
KDM4A	0.385021827	0.023779089	0.199694624
MFSD3	0.24455492	0.026655017	0.199987084
TBP	0.002532871	0.001007355	0.199998928
TLE4	0.089787593	0.002436383	0.200020901
OSBPL2	0.005865706	0.134139755	0.200501784
PPP4C	0.001635249	0.031493007	0.200616663
AL392172.1	0.432646531	0.048471401	0.200764729
PRPF39	0.000627132	0.070460803	0.200779852
LSAMP	0.019025284	0.225200933	0.200894842
RNF141	0.161352516	0.007579174	0.201039856
MET	0.025851607	0.000382261	0.201560703
BTN3A1	0.000589661	0.022299551	0.202410456
PDE8A	0.111931958	0.007803397	0.202577624
TMEM165	0.01397448	0.000710864	0.202834835
CENPL	0.035077701	0.287068061	0.203996773
B3GAT3	0.005473746	0.10903958	0.204101357
ARL14EP	0.01019167	0.214644515	0.20412493
CS	0.001038312	0.030905081	0.204132365
IRF2BPL	0.207711227	0.008840168	0.204509253
SPTLC1	0.208383416	0.006003287	0.204918531
OCIAD1	0.000467196	0.010763164	0.205235724
SH3GLB1	0.025946169	0.001520904	0.205291702
PKP3	0.015205782	8.13263E-05	0.205570479
NDUFA2	0.023844386	0.209936548	0.205582333
COQ4	0.013216329	0.330301659	0.20563577
ADCY7	0.039533512	0.225200933	0.205797949
ADRM1	0.000503453	0.007433983	0.205886145
FAU	0.026994689	0.267496121	0.206165784
CDS1	0.131233465	0.007570003	0.206469464
TTI2	0.535553713	0.035939669	0.206693065
ERLEC1	0.095305718	0.001655499	0.206750837
CASP4	0.411345683	0.027867714	0.207047554
TP53BP2	0.158135127	0.009739995	0.207109996
MED26	0.39531448	0.039013221	0.207176715
EIF2AK4	0.001332408	0.042998579	0.207273611
SEC22C	0.008981842	0.134744595	0.207359145
MAF	0.013013115	0.048324062	0.207550762
AUP1	0.020401195	0.162563213	0.2077437
SMURF2	0.01615227	0.330500779	0.208332244

TMED1	0.003681304	0.023694776	0.208747838
GPX7	0.043940208	0.032918933	0.209557033
RGS6	0.326384434	0.022443816	0.209585781
CNKSR3	0.127737636	0.013412247	0.209643936
RGS14	0.022645375	0.302929066	0.210079742
NRF1	0.036991938	0.534306814	0.210137735
PCGF5	0.117430723	0.005515108	0.210220738
PHLDA2	0.138690517	0.004250659	0.210542626
EHMT2	0.031133213	0.411790669	0.210737476
FXN	0.021147404	0.267631569	0.210944603
DAB2IP	0.005581306	2.61854E-05	0.210954643
ZMYM4	0.048831326	0.544119917	0.211222472
MUTYH	0.239003352	0.047918087	0.211297245
ILF3	0.028364668	0.53863042	0.211321431
MIER1	0.298691008	0.025710975	0.211797426
ALDOA	0.049143227	0.389031882	0.212088841
VCL	0.002278934	0.020033794	0.212095777
ZNF512B	0.043106154	0.441292982	0.212217953
ATP5F1B	0.025030849	0.313481536	0.212431637
AL358472.3	0.021599362	0.225200933	0.212558156
CTDSP1	0.007003571	0.292062261	0.213639184
SLC44A4	0.06006475	0.001272605	0.213942572
PAAF1	0.009263558	0.142837081	0.21410671
CARMIL1	0.027908455	0.31302377	0.214308635
SRSF4	0.046487418	0.001755907	0.214332979
ARID5B	0.402583072	0.04239581	0.215084074
HLA-DRB1	0.377781963	0.04156235	0.215324449
SNX13	0.17913677	0.007360342	0.215442828
GMIP	0.035598924	0.41197075	0.215472324
ETV7	0.007351162	5.87698E-05	0.215844248
NFE2L2	0.006783732	7.56018E-05	0.216116108
ANAPC13	0.144083851	0.00932658	0.216212002
SNAP29	0.003482453	5.95377E-05	0.216661485
SIPA1L1	0.011229847	0.000732215	0.216797634
TBC1D24	0.239003352	0.032714865	0.217194738
PWP1	0.52231436	0.037738576	0.217197487
PTBP3	0.001434532	2.52282E-06	0.217508068
XRN1	0.003477089	0.151631715	0.217535715
SMIM25	0.027534412	0.225200933	0.217919832
CRNKL1	0.047515834	0.382306859	0.218269786
BET1	0.001577053	2.80716E-05	0.218300165
ZNF710	0.378204777	0.033963213	0.218386285
KDM1A	0.005264863	0.037833069	0.218529339

STXBP2	0.326121356	0.025994742	0.218674144
TOR4A	0.015673459	0.000463754	0.218915866
CYTH1	0.358669451	0.039304119	0.219585541
COMMD5	0.254445644	0.02963638	0.220037676
SH3GLB2	0.283540711	0.021298321	0.220168641
PPP2R5E	0.050284217	0.000851876	0.220576481
ANXA5	0.039015969	0.464412237	0.220732909
CHMP4A	0.004951425	0.094049891	0.221342918
SLCO3A1	0.01658848	6.67156E-05	0.221638483
DDIT4-AS1	0.239003352	0.046451249	0.221849734
MICU1	0.081405956	0.002180962	0.22258358
ELOC	0.010973548	0.144714118	0.222739654
TRMT44	0.142410481	0.013547116	0.222813518
AC026979.2	0.011236796	0.099583465	0.222873306
KIF21A	0.005242473	0.00010324	0.222943908
TUFM	0.022793185	0.211160986	0.223197055
PTPRK	0.22759333	0.022042603	0.22346224
BRAP	0.404103585	0.019325883	0.223788145
IRAK3	0.002150902	0.014755931	0.224171692
RNF145	0.048102921	0.356833964	0.224225998
ASB8	0.001584562	0.002757723	0.224234692
ZFAND2A	0.164232036	0.00925027	0.224353729
IL17RC	0.289612511	0.021255221	0.225072935
MT-CO2	0.000455838	0.00457672	0.225203354
SH3RF1	0.307352207	0.034780226	0.225203708
RFNG	0.082816724	0.004285052	0.225210638
MINDY2	0.014611358	0.142726592	0.225702572
RNASEL	0.468480932	0.007075396	0.225713764
DHX16	0.021508964	0.000928519	0.226109853
SUMF1	0.392943229	0.026451862	0.226149087
MPC2	0.303361966	0.021617795	0.226313201
USP18	0.051312389	0.001272959	0.226665817
CREBL2	0.021365508	0.00194272	0.226989014
VAC14	0.011573163	0.197450806	0.227236372
UBTF	0.17070316	0.008127861	0.227489499
PMEPA1	0.003251739	0.000931058	0.228571142
PSMB1	0.010380599	0.100853702	0.228869809
SLC2A13	0.00547454	8.34088E-05	0.228940795
SLC22A18AS	0.090825472	0.005817666	0.229003155
CHMP2B	0.01146841	0.00032126	0.229553199
PGK1	0.011980219	0.094945777	0.229604092
C4orf3	0.046781213	0.35391324	0.230076399
SCCPDH	0.383669203	0.023550686	0.230469733

SLC41A2	0.193539448	0.023221615	0.230888978
NTAN1	0.038519416	0.296194956	0.231306899
TUBB	0.043316668	0.237145255	0.231474853
ENDOV	0.027808291	0.39054204	0.231616596
TECR	0.042260194	0.322838475	0.231817659
MRPL1	0.034816383	0.299491385	0.232077531
SNW1	0.004677663	0.0622566	0.232456589
KLC4	0.036330606	0.406482896	0.232459406
FKBP2	0.00395461	0.044962554	0.232461696
SP100	0.003025661	0.000200065	0.232676992
BMP2K	0.095737402	0.005622513	0.232968499
PPP2R2D	0.027611645	0.000861315	0.233294534
SLC10A7	0.020061554	0.000734878	0.233329891
AP006621.3	0.044120189	0.225200933	0.233952859
CLCN2	0.047513221	0.374751146	0.234115114
ARL6IP1	0.003904357	0.000112494	0.23435311
ATP1A1	0.047072331	0.005466335	0.234516904
LSS	0.014000975	0.224824319	0.234574729
RAB11FIP2	0.05755471	0.004632119	0.235102821
TJP1	0.008350674	5.81267E-05	0.235425441
CD46	0.003427566	0.037828832	0.235426238
VPS13C	0.251656807	0.026493229	0.236260105
USP11	0.133637096	0.010368447	0.236431441
THAP2	0.000712997	0.006045458	0.236438055
SNX3	0.012161686	0.117564186	0.236850675
PSEN1	0.195605957	0.023198195	0.23690367
FBXO31	0.299706102	0.041421585	0.237069634
ZBP1	0.001091764	0.009173502	0.237468437
ANKRD9	0.003525015	1.94092E-06	0.237489352
MKRN2OS	0.26924622	0.031585552	0.237713421
FCHO2	0.18207472	0.006513756	0.237973053
RPH3AL	0.093314033	0.018203402	0.238118549
NUDT2	0.031875716	0.340555754	0.238390227
ZDHHC4	0.026148128	0.209852189	0.238770313
RTCA	0.007657176	0.148334098	0.238845004
SF3A1	0.094519594	0.011330185	0.238857053
PPP4R2	0.035717041	0.000173126	0.239191332
KIFAP3	0.010241969	0.271625132	0.239284101
NAP1L4	0.130460704	0.002994174	0.239305565
ELOA	0.000498684	0.022913505	0.239633254
TSPAN13	0.0868422	0.003651918	0.23972967
EHMT1	0.12470218	0.004188385	0.239738462
NUBP1	0.410733374	0.020798177	0.2397606

IGF1R	0.18874076	0.026004992	0.239797423
OAS2	0.001810044	0.050622952	0.239819687
TMEM39A	0.018318393	0.175799485	0.239961558
TAF15	0.027411259	0.179192429	0.240123775
ETS2	0.124034216	0.003753041	0.240243229
RBMS2	0.027574614	0.001075847	0.240556677
PMPCA	0.021276714	0.204675576	0.241032648
GADD45B	0.032997927	0.000273427	0.241587468
SLC39A6	0.090829934	0.008682049	0.242551206
FHDC1	0.011198873	0.131080746	0.242618679
DYNLL1	0.133349151	0.00387328	0.242673935
BTN2A1	0.069560978	0.001769349	0.243165859
RNF4	0.041596445	0.002876798	0.24339657
CYCS	0.241213297	0.018615846	0.243464812
ZSCAN30	0.016734645	0.174000486	0.243704561
ELF3	0.011318843	0.000332154	0.244253626
BCL3	0.045218949	0.387293211	0.244284733
GBA	0.002281559	5.54579E-06	0.244403002
FBXO34	0.035977049	0.005984724	0.244451676
ATG4D	0.362550399	0.03557342	0.244507901
MAGI1	0.213762109	0.027582282	0.244828868
AL365226.2	0.455384464	0.041341545	0.245182392
SMIM6	0.079342218	0.007528825	0.245422686
TAF7	0.012698361	0.000553947	0.245586196
OLMALINC	0.044355285	0.401723638	0.245617673
AURKA	0.012875474	0.029531378	0.24573263
EEF1A1	0.022162133	0.300514675	0.245803989
NFKB1	0.011170732	0.145196506	0.246493868
DNAJC7	0.046746545	0.294534148	0.246516341
SELPLG	0.538557688	0.036402417	0.246693202
PHACTR2	0.085561683	0.008583952	0.246974921
TCF7L2	0.09959889	0.009413998	0.247269297
MYO6	0.285513189	0.032651546	0.247438198
IL22RA1	0.123762613	0.005043226	0.247543632
ATP1B1	0.079973774	0.00282588	0.24805794
PARP4	0.019496826	0.19832293	0.248768422
STAM	0.027194798	0.268538994	0.249110075
TNFAIP2	0.02261729	0.082270475	0.249153674
TAB3	0.24618099	0.022079446	0.249297007
IFRD2	0.341201202	0.022245853	0.24935638
SLMAP	0.013597143	0.000488642	0.249370907
UXS1	0.038749373	9.39347E-05	0.249485663
ERGIC3	0.020408092	0.000384173	0.250103035

CDC42	0.024139115	0.000609454	0.250186079
RICTOR	0.009688898	2.57905E-06	0.250347764
FAM208B	0.035138645	0.310880923	0.250461177
C19orf53	0.020944902	0.193113841	0.250703049
KIAA0355	0.461419214	0.049076487	0.251116982
PPM1D	0.033283027	0.080104792	0.25113701
STAT4	0.009530246	0.103184971	0.251241239
AMMECR1	0.323801921	0.046909695	0.251242313
CCSER1	0.34154159	0.042699071	0.251632128
HDLBP	0.360483343	0.04638075	0.252287457
GPS1	0.001756319	0.086411307	0.252643637
CHMP1A	0.253339129	0.027503896	0.253072201
NBL1	0.003880818	0.037010282	0.253484198
C19orf33	0.300581276	0.023181292	0.253740377
GSDMB	0.033880454	0.000913733	0.254086937
IFI27L1	0.302974255	0.040616779	0.254228531
AGO2	0.008828577	0.034222551	0.254775006
CD3EAP	0.026825767	0.171923147	0.255155012
NFAT5	0.025022009	0.200799377	0.255174688
DNPEP	0.004204651	3.58777E-05	0.255259099
FMN1	0.073969559	0.007723027	0.255421362
NR1D1	0.147703214	0.000169559	0.255528046
CSTF2T	0.016549432	0.19197684	0.255622591
ETHE1	0.013874619	0.143509639	0.255662752
NT5C3A	0.063113821	0.002190757	0.255753398
PHKB	0.200227435	0.008909459	0.256169453
HNF1B	0.263324001	0.019126772	0.256206921
PITPNM1	0.289528324	0.035209746	0.256309278
TRABD2A	0.022717547	0.291828116	0.256441042
CXCL5	0.004446923	7.47067E-05	0.256793202
MAPK3	0.314407047	0.044478751	0.256874882
CLN3	0.289531066	0.028163861	0.256881871
IRS2	0.111932215	0.001421042	0.257317502
DGCR2	0.072740482	0.006580726	0.257694232
AP3D1	0.035326844	0.401971409	0.257876592
PCNX3	0.018494206	0.255039973	0.258321793
SZRD1	0.258186308	0.031604437	0.258489029
PPP2CB	0.247994219	0.028743582	0.258718062
SMDT1	0.045246527	0.307893959	0.259093564
ARCN1	0.045670906	0.000491842	0.259724702
RFT1	0.006336724	0.023999533	0.259888413
HRH1	0.017187167	0.050913052	0.260189765
ARL6IP5	0.042833695	0.290003616	0.260298438

CD74	0.058612185	0.00289836	0.260469759
RPS19BP1	0.155712589	0.008107362	0.260605553
CREBRF	0.001022204	6.70038E-06	0.260827208
USP14	0.013235247	0.171370524	0.261391901
GUSB	0.189774343	0.014724236	0.261505716
SOCS2	0.039287263	0.225200933	0.261556649
CDKAL1	0.003248697	0.252945942	0.261652151
UBP1	0.010575218	0.020093723	0.261843418
PPP2R3C	0.000237933	0.008262548	0.261961698
MID1	0.051817301	0.001887105	0.262589846
LARP4	0.000287236	3.64747E-05	0.262609099
ZFAND3	0.023789935	0.001202049	0.262711716
SATB1	0.011165162	0.000115878	0.262805362
CMIP	0.005978749	0.086236296	0.262894746
SLC1A1	0.062377208	0.011233834	0.263105519
COL17A1	0.074330926	0.000742377	0.263457622
ZNF468	0.236434983	0.018388662	0.263776697
BST2	0.023189826	0.219843157	0.264223645
FICD	0.045028731	0.360702588	0.264506612
AC093525.6	0.107667493	0.008587281	0.264604958
PRRC2C	0.0001998	0.006367013	0.264800736
CLDN7	0.042264375	0.001614519	0.2648859
MPP6	0.138735182	0.027842657	0.264966802
SIAH2	0.035939118	0.450769481	0.265047206
ADAMTS6	0.021876047	0.041976038	0.265316131
PDK3	0.305825003	0.03202782	0.265605865
ZMAT2	8.3147E-06	0.000312664	0.265809167
HLA-E	0.000177816	1.65445E-05	0.265908671
OSBPL10	0.076914219	0.012014922	0.26621958
IFNGR1	0.021480775	0.002342086	0.266343753
LARP1	0.063472914	0.003108375	0.266504473
FOXA3	0.172220111	0.035101984	0.266541322
NDC1	0.02786317	0.304927659	0.266558654
OCIAD2	0.144552781	0.002829007	0.26665867
DRAP1	0.021108806	0.197139535	0.266777034
RNF114	0.002751504	0.031716782	0.267322938
ARSA	0.009586152	0.095241381	0.26733194
SRSF6	0.272640796	0.041627951	0.267576972
LINC01426	0.028458324	0.036482987	0.267918121
SLC35D2	0.09189051	0.009192018	0.267992566
IFI35	0.167771232	0.011696475	0.268236113
RELA	0.179683265	0.015060816	0.268369102
LRRC37B	0.00513251	0.060093057	0.268587529

GNG5	0.033862935	0.000455712	0.268604402
SLC9B2	0.00927815	0.04230259	0.268784777
RFFL	0.118990936	0.006035383	0.268822745
GALNS	0.245768971	0.029921907	0.268969315
EIF3M	0.044749482	0.36510648	0.269111505
ALCAM	0.048781545	0.00055991	0.269298113
MTOR	0.194083484	0.018426477	0.26934499
DIP2B	0.002751295	0.101826744	0.269649734
COA5	0.005310669	0.00011734	0.269738266
PURB	0.042504315	0.003856731	0.270009817
OTUD7B	0.242009065	0.008366152	0.270308581
RNF2	0.008399318	0.096453382	0.270902251
ZNF555	0.015869068	0.114827104	0.27098779
PTPMT1	0.072374956	0.006052574	0.271138578
PDAP1	0.288935772	0.029197414	0.271558117
PRDX3	0.029399154	0.137134041	0.271953871
STX12	0.008063148	6.51005E-05	0.272217413
DAZAP2	0.06269988	0.006292113	0.272279003
CDK17	0.312083038	0.02278879	0.272289534
USP42	0.36492559	0.036963409	0.272366396
PRRC1	0.12044499	0.010739035	0.272413418
INTS2	0.025084907	0.203090058	0.272514688
STAT6	0.0070918	0.000169193	0.272868276
COPB1	0.015931603	0.001217716	0.273108211
UBB	0.138111431	0.005691045	0.273110608
EPHA2	0.024338763	4.18668E-05	0.273410558
INO80D	0.085373338	0.009687575	0.273607671
NUDT12	0.43280706	0.044320714	0.274546502
MTCH1	0.003126844	0.073214732	0.274760896
MYO5B	0.407803434	0.041995967	0.275474455
RABGAP1	0.238298505	0.018998972	0.275650755
TPM4	0.03567811	0.000370851	0.275711744
ZNF597	0.097633221	0.025186259	0.275774899
SFN	0.134222552	0.014472177	0.275980133
TACSTD2	0.024757329	0.000292376	0.27625372
SIAH1	0.002303901	0.000688636	0.276276269
COPRS	0.003375123	0.044309396	0.276332928
TAP1	0.005125624	9.79301E-05	0.276563946
FAM50A	0.299851647	0.028646167	0.276848798
TBC1D19	0.038007976	0.225200933	0.276967393
KLHL12	0.165177945	0.018197334	0.277029407
XIAP	0.044898161	0.294707029	0.277409608
COX7A2L	0.011286955	0.24819933	0.277477771

SLFN5	1.44225E-06	0.000432708	0.277694464
ASH1L-AS1	0.332374796	0.026853352	0.278120552
DNMBP	0.00500971	0.000208588	0.278343185
FGF12	0.040256232	0.225200933	0.278874998
ITM2B	0.019900219	0.000328265	0.278914013
CNOT4	0.360803154	0.042740075	0.279034543
RRBP1	0.013691647	0.00016083	0.279056082
RC3H2	0.105026194	0.011620513	0.279503017
MAT2A	0.004880462	0.108693384	0.279841718
PROX1	0.021023579	0.003622257	0.280235527
UBE2A	0.026507718	0.00056564	0.280547772
CCP110	0.005229306	0.013687723	0.280561226
GTF3C5	0.074337347	0.001207215	0.280604218
MT-ND4	0.000970555	0.004423771	0.281167337
LRFN3	0.030463695	0.000144334	0.281677072
PPP2R2A	0.010254046	0.000145483	0.28176497
LRRFIP1	9.31157E-05	0.003356971	0.281802283
GNAS	0.039252901	0.222723293	0.281829657
CD9	0.098496756	0.003918246	0.281848634
TAF4B	0.044984321	0.225200933	0.282210121
RIPK1	0.034880262	0.000823146	0.283167534
TRAFD1	0.002660472	0.000993965	0.283527135
SLC12A7	0.153285055	0.007396694	0.284220599
RPL34	0.045327039	0.304292078	0.284501282
SERTAD1	0.110901947	0.009121254	0.284585176
ITGA6	0.011942138	5.2604E-06	0.284918115
USP3	0.129176152	0.00836511	0.286068131
ABLIM1	0.028816464	0.184836821	0.286317819
SMU1	0.134558182	0.013155395	0.286398322
PSME2	0.011087773	0.09973536	0.286550382
PNPT1	0.038076952	0.29357244	0.286692454
ZNF440	0.142810999	0.031648769	0.286887837
LIG4	0.079211639	0.008329513	0.287021873
ZNF225	0.047981901	0.225200933	0.287142619
AC245595.1	0.020507364	0.262213081	0.287429474
RAB25	0.316506915	0.034275751	0.287669081
VAMP3	0.14664337	0.005096813	0.287883193
TMEM18	0.586737131	0.027291237	0.28813656
CALM3	0.214908754	0.021397772	0.288188419
KMT2E-AS1	0.043528771	0.00381234	0.288492227
YJU2	0.032256774	0.166978609	0.289017225
ZNF672	0.0745512	0.005340272	0.289209494
TWISTNB	0.008298008	0.220546277	0.289223568

DBP	0.02793353	0.058148813	0.289889888
LRRC58	0.276534657	0.038490578	0.289890387
PRPSAP1	0.048204803	0.274520919	0.290607175
CLP1	0.000779869	0.011774507	0.290948116
COMMD2	0.021414675	0.278167973	0.291002955
EPB41L2	0.038995844	0.231445549	0.291271869
CCDC125	0.027438124	0.000235898	0.292479552
MAD1L1	0.206519483	0.000742426	0.292479944
GSTCD	0.047941682	0.083523982	0.292504974
SLC39A1	0.001371197	0.010834958	0.292536247
MTF2	0.046764867	0.279019286	0.292561544
FRS2	0.011252997	0.000409	0.292597749
MKS1	0.047577265	0.225200933	0.292755926
ANXA2	0.083954319	0.001989489	0.294015774
THUMPD1	0.025949768	0.000713199	0.294647614
STX4	0.006030078	0.092722882	0.29501553
DEK	0.497721127	0.039876135	0.295687729
CASP8	0.009071834	0.002607024	0.295830756
TAF5	0.04380097	0.329858474	0.29601769
KCTD9	0.226155403	0.034764858	0.296163243
PIGH	0.001928858	0.013077712	0.296376632
SERTAD2	0.000748205	6.1737E-06	0.296526742
SRPK1	0.002152947	0.039774256	0.296638615
IFT80	0.049612723	0.333169779	0.296933735
LINC01137	0.105261297	0.005842773	0.296938565
ERCC2	0.008929739	0.225200933	0.297114293
EIF2AK2	0.062465929	0.009941273	0.297316054
LUC7L2	0.050074187	0.002713994	0.297503468
PSMC4	0.004020723	0.065955418	0.297553003
COQ9	0.023251079	0.001590214	0.297646515
ARFGAP2	0.282409746	0.027636887	0.298138856
HECTD3	0.259698619	0.028598231	0.298623249
ZFP37	0.099816684	0.034914917	0.298795862
STXBP5	0.41333686	0.03027116	0.298899451
TMEM41B	0.001269005	0.007520412	0.29985192
TBC1D22B	0.223792377	0.006614191	0.299891503
SLC35D1	0.114828545	0.024516475	0.299945897
PIK3R5	0.031321369	0.180470148	0.300214747
TRMT1	0.011156325	0.222866235	0.300394472
GRIPAP1	0.044114383	0.003872064	0.300968931
CHIC2	0.025797027	0.00273242	0.301162186
S100A13	0.041160281	0.000477986	0.302148801
HIVEP2	0.019505018	0.16031897	0.302568858

ACIN1	0.241266163	0.02552297	0.3025813
ECI1	0.06067344	0.006263446	0.302729416
TMEM179B	0.108687221	0.007152584	0.303154111
TP53I13	0.275328263	0.031768885	0.303167638
WDR82	0.046460065	0.005247123	0.303182153
ZFYVE16	0.039308421	0.001468962	0.303471923
RFX2	0.094417035	0.035288997	0.303563869
UBAC1	0.254103805	0.037425283	0.303699862
NNT	0.140681499	0.024267387	0.303761286
UBAC2	0.075865776	0.004855987	0.30390264
KNOP1	0.037543543	0.001673288	0.304092596
CDKN2B	0.216798249	0.007230663	0.304506151
REEP5	0.024158884	0.151617337	0.304532492
BZW1	0.108702986	0.005423205	0.304716834
AC005332.7	0.157949052	0.005553994	0.305109085
WASHC4	0.001252025	0.019530119	0.305340282
BTG3	0.037383915	0.268117925	0.30545573
PTPN2	0.037542894	0.233413704	0.305592203
ACVR1B	0.023835849	0.00053255	0.305787002
BCL2L13	0.003585367	0.000209654	0.305846516
UGGT2	0.004258714	0.057898646	0.30605872
SDF2	0.044678122	0.261198973	0.306075785
CCDC43	0.244587367	0.03754274	0.306321715
SFSWAP	0.049795603	0.005189929	0.306412128
MAF1	0.185521174	0.011303314	0.306414562
LRRCC1	0.003736695	0.03301126	0.306705411
DOHH	0.084188414	0.006777873	0.306909248
GNAI3	0.022047778	0.001385064	0.307025599
ANKRD12	0.088426573	0.007871388	0.307028137
EIF4E2	0.186574219	0.018019659	0.307057348
DPP3	0.009189071	0.001660462	0.30711145
MT-ND1	0.000199953	0.002309039	0.307447198
C1orf112	0.359608838	0.049920071	0.307537108
TPT1	0.016291723	0.000232872	0.307645348
PARL	0.001513667	0.006602368	0.307730224
FAM222A	0.027032099	0.001337066	0.307908757
PTPRA	0.025451811	0.009616097	0.308307833
PIM1	0.006923072	0.037497082	0.308540791
POLR2M	0.086702994	0.009222983	0.308950706
KDSR	0.010291449	0.000269398	0.309178974
SLC8B1	0.018854926	0.000322769	0.309380048
CDK16	0.00704409	0.000152334	0.309429424
GCDH	0.038986164	0.395540385	0.309580716

SPIN4	0.09670882	0.037896408	0.309787023
CDA	0.049740424	0.213056036	0.311824118
TLNRD1	0.019564867	0.001807196	0.311830186
NBEAL1	0.040309415	0.322698394	0.311995534
LCLAT1	0.012324677	0.172285526	0.312115582
AL139246.5	0.040495465	0.160624473	0.312173685
CHMP2A	0.016613064	0.00081001	0.312405749
BEND3	0.404190852	0.031080577	0.312788319
CSRP1	0.011485059	0.104285034	0.31297935
CCDC93	0.007869621	0.095890416	0.313741607
NELFB	0.028541455	0.264307044	0.31404153
SNIP1	0.040795257	0.219681603	0.31409633
IP6K2	0.015657104	0.00809831	0.314441638
ARPC2	0.031008927	0.140450089	0.314500667
TMEM129	0.024066714	0.194859634	0.314558512
TMEM242	0.005106423	0.1657805	0.314938318
WNT4	0.545603043	0.040348235	0.315245598
POC5	0.098231424	0.007737618	0.316201904
TOR1AIP2	0.000803445	5.35205E-05	0.31652355
MARCH5	0.002865634	0.032687677	0.316573757
ZNF438	0.018708089	0.160862395	0.31679307
WDR55	0.225164241	0.035315069	0.317051155
FASTKD1	0.004553125	0.10363661	0.317097857
MAPRE2	0.114596506	0.003984208	0.317221048
COX4I1	0.070691824	0.001794201	0.317370722
VPS26A	0.08073571	0.005175353	0.317874921
IL15	0.008787538	0.083147154	0.318477139
AP003486.1	0.09670882	0.031733967	0.318486257
UACA	0.055472723	0.002116782	0.318836106
PGM3	0.321943976	0.030098016	0.318838203
NFKBIA	0.003949923	0.055713813	0.318859734
UQCR10	0.350900539	0.029955469	0.319206232
PDPK1	0.058395757	0.007393814	0.319926059
KYNU	0.004078605	0.14223827	0.320408605
DHCR7	0.011061391	0.074463622	0.320467757
OTULINL	0.254286029	0.020266576	0.32058409
VARS2	0.091538177	0.015262009	0.320896211
SDCCAG8	0.024574672	0.213943701	0.320968318
MYL12B	0.008051638	9.44296E-05	0.321238738
ZSCAN31	0.278259479	0.04290834	0.321635671
MEF2D	0.016759884	0.00073042	0.321889689
KLHDC4	0.003520542	0.008450094	0.321956631
CCNH	0.086477988	0.010108858	0.322461412

LURAP1L	0.114514456	0.020155426	0.322944478
FKBPL	0.011696869	0.125209489	0.322987235
HMGN4	0.045681944	0.026992199	0.323117489
SCOC	0.15728332	0.010624955	0.323238158
IL32	0.022586992	0.000895604	0.323425705
XKR9	0.00032408	0.00393546	0.323749112
ATP5F1D	0.026751098	0.187431861	0.324271205
UFM1	0.016579067	0.177650992	0.324461861
ZNF606	0.043574995	0.225200933	0.324473684
ZNF117	0.02895063	0.041998611	0.32492582
CCDC57	0.021228069	0.188792605	0.325197841
TALDO1	0.048662832	0.255352316	0.325216266
RETREG2	0.07505041	0.010291568	0.325321281
PRPF40A	0.007277696	0.096991987	0.325331121
MB21D2	0.279125126	0.012444568	0.325549718
TOM1L1	0.315348794	0.039565089	0.325845793
NUB1	0.037345095	0.006481807	0.325947253
NSF	0.264569264	0.044230705	0.326241645
FBXO8	0.073916048	0.007707818	0.326588446
AC004130.1	0.038342883	0.093327347	0.326857377
GAA	0.153185112	0.015077442	0.327766877
FOSB	0.032103993	0.002513318	0.327816172
PLBD2	0.11815757	0.004290658	0.328316834
ZNF37A	0.023464979	0.14234743	0.328517713
STK26	0.015233463	0.062064992	0.32878762
GCFC2	0.097500646	0.011523046	0.328800918
HSPA2	0.239003352	0.034964383	0.32891347
PRKCI	0.165029574	0.0131282	0.329066817
SDCBP	0.131709248	0.013617557	0.329079923
ZNF570	0.044559243	0.225200933	0.329302326
ADCK2	0.159491257	0.037829978	0.329572883
PPM1B	0.172933268	0.027795184	0.329823345
SRP54	0.027138921	0.148992785	0.330181229
CDK2AP2	0.024586796	0.248625799	0.330269573
RAC1	0.166341275	0.028609736	0.330317279
PHETA2	0.0030216	0.082998907	0.330543001
RAD51B	0.191369982	0.02789363	0.330821582
GTF3C3	0.026185328	0.206266455	0.33114372
CTBP2	0.009608654	0.000206102	0.331611579
ERP44	0.030304532	0.002066564	0.332049664
MPZL1	0.009094458	0.073068207	0.332078966
TMEM161A	0.018435909	0.170834503	0.332225984
CMTR1	0.043148635	0.252553929	0.33325815

ATXN1L	0.04204188	0.010244503	0.334368858
SAMD4A	0.479112029	0.049212632	0.334633846
UHMK1	0.165563536	0.022526991	0.335018504
PTPN14	0.024048418	0.165576743	0.335636053
GTF2I	0.20382307	0.044031333	0.336081455
GDA	0.243951981	0.036306224	0.336348857
SUPT20H	0.048628617	0.315174354	0.336651154
NDFIP2	0.224348606	0.038320317	0.33670103
FAM91A1	0.068881285	0.001505866	0.336960365
DUT	0.245144483	0.038963383	0.33717906
MRPS35	0.2271253	0.013363351	0.337190383
PLXNB2	0.212249569	0.032562155	0.338123701
SEC14L1	0.00444182	0.039851002	0.338377605
EPB41L4A-AS1	0.044456103	0.289002348	0.338558856
TPPP	0.162208173	0.003741314	0.33887033
HELQ	0.004686579	0.037121914	0.338926603
NFATC2IP	0.000892993	0.009890508	0.339093007
SPCS2	0.020589288	0.094760126	0.339167193
AP3B1	0.003625635	0.041438597	0.339222937
AAMDC	0.018568897	0.083316929	0.339382299
SMARCD1	0.001531541	0.010724565	0.339469027
MARCH3	0.198796791	0.007213033	0.340162991
EIF4G2	0.081739022	0.006223529	0.340230779
FAM219A	0.154636606	0.030601773	0.340921475
LARS2	0.04106632	0.338052462	0.340949251
POLH	0.035650629	0.197803274	0.341175904
IP6K1	0.219378044	0.035471214	0.341313748
RAB14	0.29366869	0.039376682	0.342274868
MORF4L2	0.038124811	0.004277535	0.342504318
AHR	0.027650896	0.002450663	0.342513936
HID1	0.206167912	0.047272685	0.342551134
NEDD9	0.007623421	0.000228552	0.34266376
MFSD11	0.033507325	0.101539237	0.342793607
FCGRT	0.284322597	0.048622711	0.343341412
UBLCP1	0.000268152	0.000307273	0.343545526
BCL2L14	0.05556374	0.005168225	0.34386403
B4GALT1	0.006898904	0.04389226	0.343872913
PRRG2	0.043292482	0.259402916	0.34397473
IFT172	0.078722608	0.013199819	0.344166305
PPP1R12A	0.000493602	0.018424003	0.344236665
GLRX5	0.149996569	0.011823558	0.344638714
SETX	0.034665863	0.174407642	0.344905333
CNEP1R1	0.087791721	0.006719154	0.34491725

CLCN3	0.017311786	0.05295095	0.345866369
GIN1	0.04321141	0.317658579	0.345971924
MARCH9	0.347617858	0.031038886	0.346113954
FBLIM1	0.071974468	0.005073012	0.346271665
AAED1	0.038775367	0.186612045	0.346303735
SH3PXD2A	0.001370297	0.011062572	0.346308544
PNPLA4	0.174212202	0.024308317	0.346557124
ZNF580	0.120724193	0.016121898	0.346985456
PDGFA	0.200543769	0.028585406	0.347013063
IER2	0.01559923	0.00031767	0.347217176
PSD4	0.002463171	2.27386E-05	0.347225551
DCAF10	0.001877422	0.000108848	0.347486529
CNKSR1	6.81804E-05	0.001890831	0.347589268
ASPH	0.013213925	0.054989658	0.34807662
HIGD1A	0.299637	0.040575708	0.348087557
PAN2	0.239003352	0.014384811	0.348247422
SEMA3F	0.025178836	0.000466373	0.348303766
ATXN2	0.080688406	0.005403107	0.348473088
NR1D2	0.215317378	0.027722309	0.348581474
SHOC2	0.090302284	0.00604907	0.348699717
SAMSN1	0.017297286	0.19181661	0.348889874
PPM1F	0.04969828	0.384267074	0.349062525
CCDC71L	0.039982713	0.302526594	0.349111197
MAP3K11	0.081661579	0.006064267	0.349483581
TRIM5	0.001410756	0.015920656	0.349549438
FNTB	0.023400483	0.001847305	0.349790171
RNF103	0.065915194	0.003649834	0.349795304
LRRC41	0.00893308	0.015753031	0.349841346
DIAPH1	0.001783128	4.19047E-05	0.35043187
ELMO2	0.137954901	0.015248146	0.350646921
HP1BP3	0.028885557	0.151170226	0.351475396
MBOAT1	0.021237839	0.098722665	0.35151673
RNPEPL1	0.110160725	0.013105261	0.351727302
SLC7A1	0.138316599	0.011363141	0.351788366
REPS1	0.004561842	0.00319654	0.351833488
SEC23IP	0.098719947	0.032386759	0.351965346
ADGRG1	0.158573991	0.038495347	0.35198852
TTC9	0.2181001	0.044181597	0.352060886
DPM3	0.009248286	0.053748547	0.352357046
GNPDA2	0.017858482	0.222828062	0.352678468
KHDRBS1	0.139072486	0.015425852	0.352804862
DCTN1	0.005621859	0.079151358	0.352972869
ZC3H15	0.043408745	0.003960139	0.353255463

CCDC50	0.170996898	0.02528828	0.353275439
LIMD1	0.0642498	0.011815677	0.353406456
GADD45A	0.329958862	0.024631571	0.353608206
IARS2	0.042065092	0.343734532	0.353694758
AC025580.2	0.239003352	0.034125602	0.353748216
ADSS	0.001619928	0.00835486	0.353785965
FARP2	0.373098941	0.030276424	0.353885134
CGRRF1	0.072466679	0.008908233	0.353911215
LINC00511	0.065458953	0.001512724	0.354184578
PEX7	0.026919225	0.008617752	0.354430994
ZBED4	0.117015896	0.0091162	0.354474968
RHBDD2	0.010028605	0.111119936	0.354764861
HCCS	0.021020593	0.004891218	0.354909655
TBC1D25	0.01600914	0.009602527	0.355106477
OAZ2	0.000324849	0.003958989	0.355827113
CABLES2	0.224730031	0.046746325	0.355953214
THBS3	0.022576308	0.195194514	0.356253495
MUC12	0.034620759	0.022622672	0.356750251
RNH1	0.000113508	0.005631259	0.356830212
RPS12	0.043995703	0.266754823	0.357211389
EXOC7	0.148778063	0.028444978	0.357299792
MRGBP	0.007133124	0.057033552	0.357329879
PELI3	0.113004365	0.02609654	0.357611294
CCRL2	0.032525287	0.005256577	0.357895716
CD2AP	0.012076446	0.000700927	0.357998131
STAU2	0.000549862	8.5269E-05	0.358519492
CA11	0.044525686	0.225200933	0.359182565
HAUS2	0.11088726	0.010361497	0.359278795
NPEPPS	0.265339249	0.027912867	0.35932162
RHOV	0.218196625	0.036569959	0.359365357
NUCB1	0.000992706	0.027295764	0.359983885
IQGAP2	0.063678215	0.008918059	0.360005397
GCA	0.286707758	0.031692947	0.360022631
COX16	0.137786312	0.013425008	0.360115504
PDSS1	0.039762503	0.225200933	0.360426347
TMF1	0.000118044	0.002014888	0.360443825
EI24	0.034193266	0.000442851	0.360959754
SUPT16H	0.062323179	0.010304371	0.361012114
GTPBP10	0.013822167	0.060272478	0.361034922
MAPK1IP1L	0.155527135	0.032150318	0.361076291
HHEX	0.05565106	0.016782252	0.36107869
SLC4A1AP	0.0358914	0.16818816	0.363184785
EFHD2	0.012953506	0.001823377	0.363335184

FAM177A1	0.004365946	0.02258035	0.363568141
SEC22A	0.227792543	0.033971884	0.364274132
VTI1A	0.180803062	0.019863507	0.364509138
HSD17B7	0.02934477	0.036516296	0.364586002
TTYH3	0.045655513	0.21524447	0.364614905
MAGOH	0.028154172	0.191486447	0.364636929
SORT1	0.004273794	4.13595E-05	0.364905209
ATP5F1C	0.040713865	0.165835564	0.364920335
ABCD4	0.009717584	0.038660714	0.365091147
SSNA1	0.295034869	0.041421887	0.365093768
ZSWIM8	0.004976786	0.028254404	0.365594606
YTHDF3	0.013882914	0.00019967	0.365670877
ZFP41	0.093823629	0.046723583	0.365864496
POGK	0.033196672	0.011161626	0.366223561
SRSF3	0.072702371	0.004143523	0.366587168
APRT	0.001411939	0.013315799	0.367555064
TTC1	0.234667402	0.020574618	0.368234548
MT-ATP8	0.005339429	0.045621844	0.368483826
KIF18B	0.039779131	0.225200933	0.368953588
PNISR	0.021347038	0.133620863	0.370252844
IER5L	0.18730856	0.030771972	0.37046952
C1orf35	0.029936787	0.244174948	0.370784072
PPAN	0.025198382	0.193620025	0.370940598
MEST	0.037507708	0.220684114	0.371013566
BPTF	0.00529286	0.027946365	0.371073536
PPP2R1A	0.016106205	0.100520145	0.371137767
RRAGC	0.005128233	0.000367862	0.372296496
DCLRE1C	0.027879345	0.00278229	0.372496191
SMURF1	0.042782762	0.002020871	0.372879641
SAP18	0.001579361	2.3149E-05	0.373043279
RNFT1	0.213290305	0.017140812	0.373121801
ZNF462	0.019557323	0.06900064	0.373380449
SERINC1	0.001973973	5.06246E-06	0.373471185
LGALS9	0.143456464	0.023269365	0.374079948
GEMIN2	0.117692806	0.005174664	0.37421867
FAAH2	0.093184471	0.012082295	0.374344016
ATP6V0A2	0.012315668	0.113015088	0.374438971
TRAF7	0.090920979	0.013835634	0.374463131
LINC01285	0.239003352	0.033468215	0.375012272
HLA-C	0.011751032	0.000817127	0.375219917
TMEM59	0.055941232	0.004402541	0.375383689
AC004264.1	0.09670882	0.033896032	0.375464687
NSMCE3	0.004656055	0.03921671	0.375803365

UBXN11	0.095742506	0.022254767	0.375912709
GSTK1	0.02517466	0.140520121	0.376229381
SMPDL3A	0.072984615	0.005074801	0.376333474
CLPTM1	0.122451602	0.003137503	0.376492095
HNRNPH2	0.052272062	0.002386483	0.376615272
UBXN6	0.116664376	0.015449576	0.376796608
INTS11	0.006630173	0.059491685	0.377110871
TAP2	0.004417994	0.028848074	0.377406881
DHX58	0.01588443	0.00245901	0.377532029
MORC2	0.025023352	0.247673933	0.377582319
HLA-G	0.040281668	0.021208214	0.37812461
ARHGAP5	0.050314038	0.00589084	0.378569989
RAD50	0.018694079	0.079171873	0.378787013
CCDC12	0.172606261	0.030043483	0.379040615
XPNPEP1	0.257217677	0.028857184	0.379620267
NDUFA5	0.008484503	0.000920116	0.379681612
LAPTM4B	0.18441812	0.027441168	0.379738312
SCAMP1-AS1	0.026589235	0.034102819	0.38014718
PFKL	0.012482073	0.011142697	0.380694123
SH3D19	0.071292296	0.004259122	0.381030591
GNPTG	0.043303237	0.009507313	0.381482796
NFYA	0.086480604	0.015656079	0.381828423
MAP3K4	0.027749719	0.027534324	0.38185876
AC245297.3	0.026037062	0.129328396	0.38222193
FOXN2	0.004499342	0.000215049	0.382663456
C1GALT1C1	0.066238073	0.001781895	0.382855289
DENND1B	0.024851898	0.16543047	0.383081163
PCSK7	0.00143048	0.003367155	0.383185604
HDDC2	0.001979342	0.005533265	0.38373121
ERICH1	0.175686572	0.037045954	0.383782993
CLUAP1	0.029273348	0.208127183	0.383982359
EIF4ENIF1	0.040307069	0.003754495	0.384480399
ZNF121	0.006285055	0.012345551	0.38512522
PDP1	0.015374823	0.000985144	0.385227978
DDX1	0.161000761	0.023115472	0.38524498
MYL6	0.012796334	0.034261992	0.385445941
GAS8	0.08247062	0.014321999	0.385521049
MKNK2	0.0391533	0.007404921	0.385682019
IMPACT	0.031914728	0.011264864	0.38616556
PELO	0.049372911	0.267034163	0.386270505
MUC1	0.039211551	0.003000163	0.386658048
SASS6	0.016698218	0.036033419	0.387560062
PLEKHF1	0.018085897	0.046335705	0.387803069

GTF2A1	0.07199587	0.013792303	0.388753941
SEL1L3	0.234369517	0.019169138	0.388949476
CSTF3	0.09358596	0.008009879	0.389350692
PIGM	0.035102886	0.146254764	0.389758211
ERV3-1	0.048728334	0.012701778	0.389762422
ZNF714	0.03847698	0.225200933	0.390415582
JUNB	0.060422251	0.009331049	0.390460758
JAGN1	0.037142057	0.213462988	0.390616031
CFTR	0.017397451	0.225200933	0.390669092
NDUFB10	0.007744678	0.049335199	0.390709619
SUMO3	1.47004E-05	9.13906E-05	0.390713287
TCEANC2	0.196824554	0.034832328	0.391075098
CEP135	0.313939241	0.032501899	0.391194583
PPA1	0.035044219	0.040308791	0.391219105
FAM174A	0.047041953	0.005141539	0.391327529
DHRS7	0.006529554	0.092792317	0.391338452
DNAJC1	0.11570181	0.012006558	0.391482397
C15orf39	0.075008323	0.001432336	0.39174039
DCTN2	0.003061991	0.037078671	0.391767624
TTC38	0.056682423	0.005604921	0.392529385
C12orf73	0.161821792	0.033926336	0.393906414
GSTO1	0.021564675	0.085343106	0.394014779
GATC	0.083951692	0.028181483	0.394230513
PPIA	0.025593537	0.10349473	0.394258831
RAD23A	0.047220846	0.002773762	0.394284047
CDK18	0.001637725	0.000142721	0.394310131
IRF2	0.009115179	0.063704316	0.394560507
DCTN5	0.024028266	0.154585232	0.394813951
SAR1B	0.023658829	0.002589946	0.394888299
RELB	0.034136024	0.159868344	0.395198046
DYNC1LI2	0.058658105	0.004712796	0.395252264
AGMO	0.039190915	0.225200933	0.395385523
HNRNPUL2	0.008448145	0.00025818	0.39543621
GLRX	0.046672167	0.166032128	0.395473004
TMEM56	0.045408698	0.225200933	0.395512118
DNAJB1	0.115962932	0.014003648	0.396224092
ERI2	0.019763974	0.004656807	0.396621102
ZNF260	0.082320808	0.014323717	0.397303129
PPCDC	0.015780336	0.039416571	0.397588157
TMEM184B	0.260199878	0.028963789	0.398016328
DYNLT1	0.139716039	0.017194462	0.398036763
RHOC	0.005835916	0.000351097	0.398124823
VASP	0.072221559	0.001337416	0.398553004

VPS18	0.065437755	0.004075484	0.398573712
DNAJC22	0.027552757	0.005742053	0.398671892
OTUD6B-AS1	0.152831155	0.03195465	0.398922373
ZNF562	0.049045757	0.146624359	0.39903141
HLA-B	0.00802439	0.000468353	0.399321337
CDKN2C	0.240294035	0.037653234	0.399546968
SOCS4	0.011683184	0.080151878	0.399775355
FAM96A	0.061727561	0.012134411	0.399936746
EFNA3	0.023365026	0.125992629	0.400104313
SECISBP2	0.201375413	0.039157025	0.400422706
RUVBL1	0.085334385	0.015483834	0.400775197
BCAR1	0.012862743	0.001671014	0.401220645
SCIN	0.095955561	0.008127791	0.401878672
CCDC9	0.050775476	0.00506979	0.401892946
SERAC1	0.09372887	0.015084513	0.401978854
FAM126B	0.020192502	0.009157785	0.402307448
MBNL2	0.012274019	0.058391482	0.403423976
RREB1	0.021075025	0.076668992	0.404105755
PHGR1	0.11841785	0.019523359	0.40456078
NRBP1	0.066670107	0.009181467	0.404801057
FOXJ2	0.027011859	0.122091818	0.405126921
POLR2F	0.033147031	0.130189143	0.405711368
PSMD12	0.024062402	0.121197871	0.405773368
FNIP1	0.002254664	2.56917E-05	0.406498214
MAFG	0.02197112	0.005364487	0.406629101
CXorf56	0.014770116	0.22927899	0.406722948
SELENOF	0.003981676	0.031364732	0.407120455
COG7	0.020173224	0.15469392	0.407130628
PHLDB2	0.168339714	0.014665211	0.407911697
TBXAS1	0.215740672	0.044600373	0.407925267
CTSB	0.023177236	0.140274207	0.40903606
NMT1	0.176806509	0.033578909	0.409951976
KLHL20	0.186541899	0.035639684	0.410123647
TMEM87B	0.004979616	0.001365766	0.410387022
DSC2	0.04438551	0.001330998	0.410806542
ACTN1	0.042448924	0.153964416	0.410861417
FLAD1	0.016585757	0.131263412	0.411609955
CYB561D1	0.097065591	0.031913076	0.411620285
SPSB3	0.007667126	0.041177535	0.41167043
TDP1	0.126233081	0.009051358	0.411867503
CDHR2	0.014026083	0.002922061	0.411938972
SYNJ1	0.016747103	0.017998493	0.41198392
BCAP29	0.022433504	0.095716138	0.411996067

ANAPC16	0.001588621	0.003982738	0.412197469
HSPA14	0.033287163	0.007307358	0.412403685
ZC3H13	0.00976363	0.000603367	0.412510192
SMIM14	0.08135648	0.002529369	0.412868707
ERGIC1	0.088255763	0.007142937	0.413689187
MTFR1L	0.080806776	0.005334905	0.413901684
ARMH1	0.010739688	0.109979558	0.414217891
PCNX4	0.06098662	0.003922231	0.414536085
RPN2	0.004526351	0.038284489	0.414995431
ECHDC1	0.001536005	0.014720533	0.415104939
AKIP1	0.042241447	0.024886444	0.41541108
DIP2C	0.120824591	0.009196925	0.415539864
C2CD2	0.020780859	0.008136741	0.415765465
STAT3	0.005959254	0.034998726	0.416066493
NELFA	0.015086062	0.000862887	0.416542104
ABCB9	0.041690134	0.225200933	0.416883164
PFDN5	0.043927511	0.149122769	0.417332319
RALBP1	0.009663342	0.00074953	0.417836331
STX5	0.023689943	0.002185233	0.417999563
APOC1	0.100177129	0.022426914	0.418243111
HES6	0.016197047	0.064923902	0.418299829
PGGT1B	0.061870843	0.005899714	0.418795586
CHMP6	0.236446318	0.019672021	0.419117283
EXT1	0.011458928	0.065723355	0.41935663
ARFIP1	0.057670207	0.014399008	0.41942575
LRRC6	0.022067059	0.225200933	0.41947466
MZT2B	0.117372082	0.007024952	0.419583461
RBKS	0.015211615	0.254205161	0.419826132
ZBTB1	0.013203217	0.106202773	0.420066286
PABPC1L	0.022021066	0.018368876	0.420192939
POGLUT1	0.007704122	0.061807672	0.420194176
PPP4R3B	0.229402	0.019991719	0.420665269
GALNT11	0.044707252	0.439338059	0.420680757
OGFOD1	0.01608543	0.09548355	0.420818372
NELFE	0.061771369	0.005998053	0.420905
TSR3	0.026533432	0.106622522	0.420954156
HNRNPU	0.012768215	0.001096157	0.421054504
TNRC18	0.04475137	0.009636676	0.421185343
SOX4	0.008289861	0.010887786	0.421252094
RNF149	9.17469E-05	0.000207536	0.421528698
SLC35E4	0.023875828	0.044690774	0.421732025
MRPL13	0.025942692	0.12067364	0.421896055
RTF1	0.161800413	0.025173047	0.422268692

ZNF747	0.100753963	0.022421166	0.422669495
MIGA1	0.005165686	0.006075438	0.423264291
FGFR2	0.093065314	0.03862345	0.423441358
ALDH18A1	0.033051095	0.0184563	0.423447233
DGAT2	0.039291201	0.225200933	0.423628654
GULP1	0.139481199	0.00118341	0.423840782
ZNF75A	0.099594638	0.036588362	0.423851046
BROX	0.039565645	0.118631697	0.425139368
ARRDC1	0.004738591	0.00027961	0.425196295
PDE5A	0.139885579	0.024060715	0.425456455
NXT2	0.011573395	0.078298295	0.425543002
PJA2	0.002228928	0.001781348	0.426567352
JADE3	0.042787397	0.225200933	0.426626418
KCNK1	0.002463355	0.000697561	0.426894692
RPL36	0.034827863	0.138089882	0.427061309
MT-CO1	0.006187569	0.029334825	0.427199815
PER3	0.22248456	0.04811603	0.427514727
JPX	0.007546394	0.001619767	0.427576327
CTNS	0.016227519	0.044864878	0.427577325
KRTCAP2	0.006627697	0.035959591	0.427928366
APOL2	0.001254892	0.03143394	0.428014503
CFL1	0.012397044	0.027971644	0.428106487
PNPLA8	0.146680611	0.049950584	0.42821223
AC040162.1	0.042509908	0.005889761	0.428232723
CTSO	0.180237928	0.043779913	0.428546028
SETD6	0.233428238	0.028276182	0.428833938
VPS37B	0.117488775	0.01235522	0.428991002
PRDM8	0.020264387	0.14610226	0.429109263
SURF2	0.038574581	0.150037339	0.42914887
CPNE1	0.006655646	0.029961542	0.429371951
EMP1	0.156810618	0.016358079	0.42993506
PHTF2	0.03722341	0.073997385	0.43085922
HHLA2	0.258394126	0.009036833	0.431178607
CTNND1	0.03620144	0.00199063	0.431375739
MBD5	0.033572642	0.130276566	0.431973547
HIVEP3	0.323830776	0.014545182	0.432001001
PI4K2A	0.034056897	0.228149531	0.432122696
UBE2H	0.015092265	0.000759845	0.432319246
ARHGEF28	0.263363236	0.043733157	0.432338357
UIMC1	0.004682446	5.16143E-05	0.43247199
KRT19	0.278208719	0.042101275	0.433176845
EIF1AX	0.108895699	0.011720011	0.433213248
DISP1	0.194854452	0.030595342	0.433224593

CSF1	0.002258188	0.022146534	0.433243358
S100A11	0.013558775	0.058215969	0.43342927
CNOT11	0.010576185	0.065291832	0.433446227
PSMB8	0.046270162	0.00205234	0.433642303
COX17	0.036984249	0.001052461	0.433746974
CWC25	0.017333577	0.000728258	0.434116856
DYRK1A	0.031086782	0.001652739	0.434157209
BEX4	0.041562192	0.083672841	0.434302523
POLR2C	0.09680151	0.019019959	0.43445697
PLAA	0.083906634	0.009008879	0.434589718
TERF2IP	0.000918136	0.004349224	0.434623948
SNRPD3	0.032359014	0.191746389	0.434884781
CDC42EP1	0.178980467	0.0178034	0.435187497
ADIPOR1	0.050748394	0.005907193	0.435893914
GABPB2	0.000459214	0.001345593	0.435908671
PPIL2	0.002919075	0.003605105	0.436057199
AP006623.1	0.003792419	0.134165439	0.437257102
AP002360.1	0.114244275	0.042070676	0.437888805
GATAD2A	0.054943393	0.007444259	0.438546071
YIPF5	0.024906089	0.135972214	0.438598861
PUM1	0.014254057	0.133191528	0.438656242
PMAIP1	0.025812096	0.031601196	0.439415226
TMEM167A	7.44557E-05	0.001798286	0.440092808
YWHAB	0.002960993	0.000176007	0.440532163
ILK	0.005144715	0.027583407	0.440604089
SENP5	0.02123718	0.055519837	0.440939441
LZTS2	0.146224528	0.023745243	0.441244385
LINC01133	0.201724983	0.032504226	0.441689746
UBALD2	0.027431344	0.055063474	0.441701039
CYBC1	0.004307718	0.023444621	0.44179606
FAHD1	4.23521E-05	0.000122842	0.442369104
SSR4	0.012598866	0.028478228	0.442644347
RBMX	0.001119875	0.019383839	0.442674971
IAH1	0.037741773	0.174009324	0.443304999
TMEM208	0.023763226	0.11278643	0.443835233
MAEA	0.047762361	0.004388574	0.44404321
SLC27A4	0.053300355	0.037228035	0.444044631
LRRC23	0.027481583	0.02515211	0.44423747
VPS37A	0.068839849	0.014833834	0.444992112
BTF3	0.036934655	0.113030634	0.445213079
PHF20L1	0.0005256	3.83903E-05	0.445457155
APOLD1	0.0491906	0.088128156	0.44548478
COL4A3BP	0.001146244	0.008495429	0.445649199

ATM	0.035016028	0.104854039	0.445861547
DYNC1I2	0.022388607	0.002970563	0.446841199
NDUFA8	0.215921267	0.042223225	0.446961404
PRR15	0.03698465	0.03563124	0.447511249
KDELR2	0.101555532	0.018235379	0.448514952
CRAMP1	0.049714462	0.019948791	0.44857489
BRPF3	0.040678922	0.01664709	0.448625907
MYL12A	0.0050516	8.10745E-05	0.448703323
SERINC5	0.047506678	0.234292549	0.448861426
MRPL9	0.057394653	0.001617509	0.449242657
SCAMP4	0.001348665	0.005168455	0.449901975
ZNF140	0.04414909	0.217161253	0.450119476
PAQR4	0.09975691	0.010968168	0.450445818
PLS3	0.003992697	0.00667952	0.450640401
AC018557.1	0.239003352	0.034609647	0.450851278
ERAL1	0.106771448	0.018491916	0.451242462
POLR3GL	0.007729515	0.042000214	0.451590352
RAB40B	0.161343575	0.023968108	0.451884782
GART	0.004350278	0.032281462	0.451971635
ATXN1	5.26506E-05	0.000242865	0.452038198
C12orf57	0.028163676	0.080472706	0.452288383
CD2BP2	0.002956944	0.022928883	0.452925464
ATG2A	0.013915835	0.001798465	0.453076117
RBM15B	0.16522441	0.007800165	0.45381142
KLHL9	0.22472262	0.035553372	0.454222187
ARHGAP35	0.038510493	0.002530566	0.455014273
IDH3A	0.058409421	0.006149492	0.455129645
SOD2	0.029994163	0.054988142	0.455176386
CTDNEP1	0.120849928	0.014342814	0.455408447
DNAJC3	0.001287981	8.007E-06	0.455474767
PACS1	0.05501678	0.009393231	0.456435485
LEPROT	2.25682E-05	0.001093601	0.456475153
TMEM203	0.141676033	0.035258298	0.456827233
SLC25A23	0.108066085	0.020371485	0.457691428
DCAF5	4.26101E-05	9.11704E-06	0.457794234
UNC119B	0.239003352	0.033754268	0.457980423
TMBIM1	0.024624268	0.00279156	0.457994969
LAMTOR1	0.101113099	0.015074169	0.458030803
DESI2	0.01010231	0.057092013	0.45844724
RAP2B	0.085144804	0.017113236	0.458654491
HDAC3	0.005725199	0.015910574	0.458900399
CAMTA1	0.007421672	0.000245233	0.458997488
RHBDF2	9.59443E-05	0.000137083	0.459384925

PPL	0.12550416	0.026576307	0.459528646
ZNF326	0.015439109	0.087668538	0.459791217
VDR	0.004230007	0.001901918	0.460415827
FAS	0.010286197	0.001686972	0.460872765
PSMA5	0.037136988	0.099089089	0.461190789
TMEM216	0.064532591	0.02115131	0.461310831
PTPN21	0.023264829	0.00318971	0.461638481
SLC39A3	0.008350064	0.04284731	0.461713203
GRTP1	0.038766886	0.015102388	0.46217695
NET1	0.011730673	0.025049772	0.462249494
SLC35E3	0.027060983	0.029346559	0.462335738
SPTAN1	0.008986677	0.000521723	0.462651386
S100A2	0.054381872	0.015529799	0.462811433
CCL8	0.027904501	0.107112479	0.463063369
CLPX	0.034807031	0.002474783	0.46314259
TSC22D1	0.00856155	0.000133011	0.464113866
CASKIN2	0.023115971	0.126545874	0.4648933
ANAPC1	0.05333244	0.002052742	0.465163608
TMEM50A	0.001962771	0.010608453	0.465622977
TRIP11	0.005997673	0.000245762	0.465875815
PTPRB	0.09372887	0.013148778	0.465912288
ANKFY1	0.007779922	0.00024477	0.465952661
ANXA7	0.000193389	0.000172105	0.465975671
TIMM8B	0.039327732	0.001594202	0.465989617
GRB7	0.054317474	0.008551741	0.466191396
PTAFR	0.002289904	0.003935208	0.466410063
PFDN1	0.009917727	0.028401122	0.466671921
SETD3	0.161740804	0.047662722	0.466944321
FAM120B	0.011032061	0.007258619	0.467041452
IFIH1	0.020251655	0.083608977	0.467102256
GALNT5	0.160333896	0.040391607	0.467291541
INO80C	0.014360061	0.000847995	0.467374123
EHD1	0.024909632	0.152091873	0.467416749
RPLP2	0.024725596	0.130557349	0.468166498
DDHD2	0.002741361	0.014396677	0.468245882
SKIL	0.034985762	0.125785704	0.469106193
MRPL40	0.042410934	0.166290433	0.469271885
ZNF888	0.108074761	0.041004905	0.4695915
NR4A2	0.142439554	0.030891599	0.469597271
OGFOD2	0.031574715	0.072489205	0.46979319
DAZAP1	0.037330637	0.123341392	0.469943472
UBR4	0.02411422	0.137313201	0.470553951
MFSD8	0.007487098	0.08910367	0.470626727

JPT2	0.034701896	0.104249635	0.470788736
UBN2	0.005107266	0.011210354	0.471378883
SCAMP2	0.225683011	0.049845842	0.471633338
DHTKD1	0.041909053	0.196496482	0.472564534
APPL2	0.180252355	0.019408513	0.472633158
LRRC47	0.057208127	0.004492325	0.472833175
PARP9	0.00519561	0.045472466	0.472863479
PDCD2L	0.187281703	0.035868823	0.472988598
ZC3H4	0.026779053	0.09728134	0.473472913
GDPGP1	0.164428187	0.043729834	0.473585871
LINC00467	0.028820942	0.201082508	0.473616453
EIF5A	0.038367498	0.11995072	0.4740911
COX7B	0.208839749	0.041596502	0.474984564
DHRSX	0.013850515	0.034064993	0.475032018
NDUFV3	0.015151113	0.026907157	0.475067928
TMEM127	0.146364025	0.02017739	0.475267328
PTER	0.023966934	0.129766604	0.47527178
SHROOM1	0.044246148	0.019107118	0.475609877
SRGAP2C	0.048816637	0.167583743	0.475771515
TRIM22	0.008965937	0.086329449	0.47582048
SPRYD3	0.018632266	0.1779034	0.476211921
DHX57	0.02986746	0.01237971	0.476223729
FZR1	0.012167069	0.087449514	0.476506707
EPHX1	0.010506517	0.041017806	0.476719898
IL10RB	0.026367351	0.09913887	0.476790108
WARS	0.033062314	0.003546545	0.477143078
ACVR1	0.06529768	0.013710057	0.477324798
ADAL	0.188167792	0.026221068	0.477666906
NTMT1	0.003229722	0.007293428	0.477957931
C1orf167	0.005399351	0.008690304	0.478003198
JAK1	0.013102656	0.08411378	0.478377079
PPP3R1	0.133858207	0.030755314	0.478501059
SSB	0.041955914	0.178246091	0.478929162
CEBPD	0.060819958	0.008785908	0.480052972
SLC4A4	0.208082671	0.015685788	0.480096915
PSMD1	0.150719985	0.043644829	0.480441929
SURF4	0.131623416	0.019263844	0.48059222
NCOR2	0.159654443	0.034967436	0.480688399
SRRT	0.130819424	0.027050793	0.480923022
TMEM120B	0.081099254	0.01459531	0.481315075
CTDSP2	0.121716379	0.044822002	0.482390043
ZDHHC12	0.033849393	0.000989707	0.482715538
C6orf222	0.010364219	0.000201763	0.482854768

ABCA1	0.121456664	0.037317677	0.482902844
HNF4A	0.028264351	0.09431176	0.483444322
AKTIP	0.019090014	0.143960186	0.483618364
PCDHGA8	0.019131809	0.099768973	0.484289648
DENND1A	0.035640421	0.006873299	0.484314048
TRMT10A	0.028954076	0.003794589	0.48439858
PSME3	0.019034914	0.097319204	0.484968349
GRHL2	0.151132207	0.038911636	0.485108909
TENT2	0.001028003	0.00179144	0.485400195
ARF4	0.018701025	0.074528224	0.485823396
SBNO2	0.00344409	0.008316383	0.485900753
UBN1	0.037579846	0.157801172	0.486064303
MRPL28	0.025210433	0.095520054	0.486448293
ATAD3B	0.022960564	0.218376041	0.486759585
C1QTNF3	0.043128621	0.225200933	0.487267375
ZFP90	0.018403494	0.116836279	0.487353485
ATF1	0.173607146	0.028864556	0.487504546
ZNF551	0.084503379	0.042690875	0.488094365
TPP1	0.111038492	0.039736717	0.4883628
NARFL	0.043418008	0.233842385	0.488401031
G2E3	0.003422264	0.075114946	0.488615147
MOSMO	0.038639125	0.09717276	0.488656215
LMNB2	0.00417844	0.000416008	0.488847142
PANK3	0.028625267	0.000789039	0.488887558
CCDC6	0.070602164	0.011365253	0.489608101
KIAA2026	0.181215957	0.02118208	0.490603886
SYPL1	0.001693272	0.006815887	0.490876058
RBM39	0.02099167	0.001493223	0.49092281
TBCEL	0.240400888	0.035202177	0.490966674
MED10	0.030120173	0.002733496	0.490984976
CGGBP1	0.13968924	0.038510239	0.491181243
GPR107	0.14764029	0.029635707	0.491516905
SGPP1	0.003165519	0.017680939	0.491884084
NRBP2	0.017357605	0.033869269	0.492429162
MORN2	0.009478699	0.064198386	0.492529807
TBC1D9B	0.196632611	0.023035379	0.492714945
GXYLT1	0.062584608	0.003152559	0.493039158
PGM2L1	0.005367257	0.000158456	0.493566906
ETFB	0.241225678	0.043265718	0.493772312
KRT18	0.31796393	0.047997403	0.494565693
WDR83OS	0.037535663	0.108981875	0.494674859
SURF6	0.001048445	0.012936221	0.494760947
ASB1	0.011009698	0.023643086	0.495153811

N4BP2	0.027346135	0.111091167	0.495466612
TEDC1	0.008072781	0.01154908	0.495487294
AC004687.1	0.094417035	0.032279434	0.497668013
ETV5	0.118056372	0.049799043	0.497871899
RMND5A	0.094475031	0.025403093	0.498045573
NABP2	0.004476091	0.036675718	0.498102188
LRRC8A	0.003072266	0.010723071	0.498694025
LIN52	0.05375988	0.006603094	0.499233028
SLC35F6	0.04072554	0.00498463	0.499360536
SLFN12	0.062940628	0.008177015	0.499407246
NAA15	0.03173876	0.117369605	0.500100654
CXCL3	0.073014336	0.021674158	0.500115134
IGF2BP2	0.027491217	0.000740847	0.500471729
MTRF1L	0.002253078	0.000333984	0.500620259
ZC3H12A	0.145869639	0.031583107	0.500696854
STARD7	0.006468913	0.000430992	0.500748414
EID2	0.026164001	0.143223744	0.501394281
IQCE	0.008427614	0.065529621	0.501536259
SOS2	0.159543284	0.025225574	0.501689161
GAK	0.010790065	0.030916695	0.501761962
TOM1	0.163009451	0.033953896	0.501818385
TMX3	0.03535572	0.127452336	0.502189272
CPEB4	0.01701184	0.079625959	0.502753701
THUMPD3-AS1	0.013512279	0.001109443	0.502908947
SLC28A3	0.003047218	0.012004716	0.50291136
PPIL3	0.046635333	0.040087946	0.503103476
SUMO2	0.013330198	0.035097309	0.503403958
LGALS2	0.054544765	0.001957052	0.503452991
COX20	0.025361772	0.105102851	0.503508809
SON	0.003249351	0.015958338	0.503689634
UBXN4	0.163753456	0.043239878	0.503981576
ZFYVE1	0.001612612	0.000961891	0.504211067
MRPS18A	0.087186487	0.02418162	0.50580508
SEPHS1	0.0956391	0.032449025	0.505841903
GLRX2	0.019613496	0.055890589	0.505996451
P4HA1	0.015444719	0.025146246	0.506181006
GATAD2B	0.042381033	0.208526838	0.506246907
ANXA3	0.046489313	0.009438438	0.50626392
CHMP1B	0.02248759	0.000374771	0.506375342
CDKN2D	0.074452309	0.001735334	0.507136206
LDHD	0.043641027	0.103274865	0.507782582
P4HA2	0.032743421	0.005456737	0.507948906
MT-ND2	0.00012684	0.000119979	0.509371082

KLC1	0.007080689	0.010335481	0.50976542
CSNK1G3	0.080074922	0.015047428	0.510191638
SETD2	0.033160028	0.008112864	0.510214514
RPF1	0.007925714	0.013092587	0.510326592
FBXW5	0.139146753	0.011727595	0.511184258
SDC4	0.021026126	0.042756736	0.511296336
UBE2F	0.0023175	0.001841643	0.511677302
TCAF2	0.15467962	0.013194288	0.51185243
AP5M1	0.173440676	0.015790261	0.512005834
BRD4	0.027053381	0.002771633	0.512553515
DONSON	0.044779306	0.318144309	0.512946544
CARS	0.038702262	0.189129974	0.512985956
TAF9B	0.016036157	0.059207186	0.513288078
TTC37	0.040309209	0.095467558	0.513302903
KBTBD12	0.096233562	0.001391846	0.513350248
ZNF33A	0.025301429	0.142921519	0.513590494
NUMB	0.051149953	0.006718973	0.514084904
HSD17B12	0.003781558	0.006504736	0.514188145
KIAA1671	0.002387188	7.80419E-05	0.514215441
PQBP1	0.02507289	0.0884717	0.514395942
RCHY1	0.032112725	0.007864969	0.514610695
CXCL1	0.036593079	0.116572233	0.515520174
BAZ2A	0.012805815	0.055102432	0.515796485
BAMBI	0.004023066	0.006418879	0.515811271
FBXO6	0.037044176	0.00166983	0.515998316
BBX	0.001080272	0.004835135	0.516345539
CUL4A	0.124003788	0.022810315	0.516617592
TEAD3	0.004747915	0.043782348	0.516710759
DROSHA	0.008269718	0.227800108	0.516963312
CEP192	0.00981521	0.072817823	0.517197336
MINK1	0.021256632	0.075876943	0.517274311
SDHD	0.100814619	0.021174746	0.517288735
DCUN1D1	0.018560168	0.002149138	0.517633716
SESTD1	0.161228114	0.032868559	0.518800167
RNF185	0.111518547	0.028131964	0.5194727
KCNE3	0.050244573	0.01533594	0.520063055
MARCH8	0.027189808	0.009605626	0.520186602
CENPS	0.027998964	0.062302687	0.520449153
GABARAPL1	0.00259773	0.00217552	0.520671927
NMRK1	0.039665924	0.26287562	0.521156314
NDUFAF1	0.003463602	0.01532787	0.52212993
AC097376.2	0.031991325	0.007690118	0.522168134
DTNB	0.040002422	0.012819984	0.522732137

LINC02542	0.114374563	0.031740348	0.522795534
APOO	0.01404828	0.113725631	0.523070397
NDUFA12	0.064885196	0.003531301	0.523148024
GK5	0.001624696	0.091110322	0.523510877
NDUFAF6	0.007088641	0.10937072	0.523526271
CHERP	0.024525216	0.009157496	0.523608267
SAMD9	0.001241525	0.001397517	0.524073064
BRI3	0.177979161	0.02926579	0.524142723
WDR78	0.047132829	0.245184057	0.524236495
DDX55	0.044032666	0.121739077	0.524341241
ZNF720	0.023513806	0.018376996	0.524523532
CAPN2	0.045571639	0.125798238	0.52458484
TMEM33	0.088594236	0.020528213	0.524815511
PHF11	0.001152181	0.001253532	0.524903431
BSG	0.000967357	8.56199E-05	0.52515684
EXOC3L1	0.131415135	0.034454209	0.525284674
ALS2	0.059044548	0.032364453	0.525629724
ZNFX1	0.002570591	2.94006E-05	0.525639556
CHML	0.032869876	0.147737931	0.526427608
PLXNA1	0.016223911	0.041071458	0.526437684
RBM48	0.046923769	0.185381099	0.526505384
MAN1A1	0.027340308	0.047802782	0.526558789
JPT1	0.002081505	0.001723042	0.526676634
MADD	0.103108342	0.029309572	0.526793318
UMAD1	0.094975304	0.024012656	0.527006739
TRIP4	0.003059016	0.010842457	0.527135753
KDM6B	0.013954596	0.008208523	0.527143271
YAP1	0.013748362	0.021039514	0.527558117
EIF3E	0.028834119	0.146067105	0.528427254
ACTG1	0.062518199	0.001187843	0.528785003
AGFG1	0.001065054	3.96753E-05	0.528860045
CD5	0.04514383	0.139179459	0.528900637
STYXL1	0.055368331	0.03148973	0.529496821
ST3GAL2	0.007681723	0.039745075	0.53004096
MVP	0.04090525	0.008806004	0.53075635
DOCK5	0.180290931	0.044788407	0.531172989
RWDD2A	0.024760092	0.086178524	0.531747577
PCNT	0.179774212	0.042261677	0.53181147
FOXK2	0.008378057	0.016261441	0.532300132
SREBF2	0.007932349	0.014674248	0.532603776
RSF1	0.098375497	0.033392707	0.532884391
BUB3	0.042614136	0.142293975	0.533564229
KDF1	0.125162874	0.024287026	0.534057755

ARNTL2	5.76331E-05	0.000112503	0.534219857
EIF4B	0.015716276	0.049944495	0.534275033
KPNA6	0.005917524	0.067440854	0.534494691
ZNF713	0.041209261	0.095079336	0.534565216
MX1	0.023905375	0.002932124	0.535153113
DTX3L	0.034938934	0.083631726	0.535796916
PPP1R11	0.000144878	0.001091722	0.536644497
CTNNA1	0.011223876	0.000706052	0.536704682
LETMD1	0.054633755	0.022604119	0.537301482
MED27	0.024867544	0.150569319	0.537832053
MAP3K7	0.067253817	0.023769857	0.538733181
AKIRIN1	0.097659806	0.022524733	0.538745925
HES1	0.096391662	0.025098157	0.539385896
SLC24A1	0.10410533	0.03940902	0.539790914
EIF4A2	0.01326121	0.006070822	0.540024306
FAM89A	0.250533123	0.04086933	0.540463853
PRCC	0.044653211	0.182079066	0.540632255
EMC2	0.034785905	0.005747824	0.540658722
GORASP2	0.033371436	0.003876965	0.54115475
LBX2-AS1	0.065931755	0.029920081	0.541551119
EIF6	0.00157075	0.002640922	0.541916707
CTTN	0.013002844	0.004838352	0.542001834
SC5D	0.042053304	0.133636062	0.542665724
PPIC	0.024761197	0.052191867	0.543646659
ABCG1	0.120668934	0.027183026	0.544046064
FPGS	0.011047002	0.038397307	0.544120764
ATP11A	0.005802509	0.000582499	0.544164391
FAM118B	0.016711281	0.058122434	0.544175446
FBXL15	0.002199688	0.025560744	0.54433057
SLC16A3	0.00010143	0.000175653	0.544571499
CASP8AP2	0.036011406	0.14375509	0.544832572
RIN2	0.052087995	0.007781472	0.545099565
SH2B3	0.037650172	0.001559918	0.546009763
ARPC5L	0.01137425	0.042468954	0.546102977
KLHL22	0.041842785	0.141642426	0.546161621
SERF2	0.05768191	0.005581503	0.546179255
AL050403.2	0.017905965	0.083313401	0.546260226
CACNB3	0.023550056	0.095980418	0.546431449
C16orf72	0.059019649	0.017184425	0.54645192
FNDC3A	0.002727533	0.000832813	0.546514098
KIAA0319L	0.003819215	0.015543302	0.546535734
HSPA4	0.017245668	0.065059512	0.546554105
IGFBP4	0.015672721	0.03166251	0.546701521

NFU1	0.01133468	0.003529507	0.546738049
CYP2J2	0.091177311	0.030078647	0.546769815
H3F3B	0.006824018	0.000273057	0.54690895
OSTF1	0.001370644	0.019325903	0.547371599
RSAD2	0.119966174	0.003161345	0.547455283
ORAI1	0.090070968	0.01089734	0.547462596
ODC1	0.11609986	0.021948567	0.548236986
YARS2	0.189686664	0.044761509	0.548240302
GOLT1B	0.015643818	0.078013476	0.5488951
ATG4A	0.031504964	0.01019134	0.549647233
ZC3H14	0.015134557	0.014801966	0.549713571
TNFRSF1A	0.08260391	0.019569471	0.549766766
SMARCA5	0.260549382	0.043324459	0.550521904
XYLT2	0.054718094	0.009222872	0.551196993
TMEM222	0.005635267	0.048893001	0.551250595
TMEM173	0.132457585	0.025802309	0.5519839
GLG1	0.023818579	0.005469783	0.552133467
CDYL	0.123949376	0.023222268	0.552242083
OPTN	0.012668441	0.001867543	0.552289525
UBE2B	0.003231585	2.22754E-05	0.552466365
TCHP	0.101898892	0.031405486	0.552720308
NDUFAB1	0.054138651	0.005962719	0.55280946
DGKE	0.002969959	0.022905511	0.552832499
ADAM15	0.070590258	0.022187727	0.553473964
UBE2J2	0.072324279	0.023197472	0.553683883
TNKS	0.018203339	0.017685332	0.55371453
CEACAM1	0.001626342	0.001013954	0.553726159
PRKRA	0.013364046	0.115972988	0.5537923
AIMP1	0.011377864	0.029553205	0.553975936
CHD4	0.009244879	0.045521761	0.554291915
KLF11	0.062230753	0.012010532	0.554333013
NT5C	0.047764794	0.140641056	0.555167619
RNPS1	0.025258081	0.002732099	0.555275653
EP300	0.024340083	0.007279403	0.555337132
CORO1B	0.008356998	0.045881417	0.555627395
HELZ2	0.07712232	0.013420732	0.556589179
CDK11B	0.025253314	0.091088053	0.556693856
DHRS3	0.019966039	0.074088664	0.557352875
BAG3	0.069313892	0.008676067	0.55761481
SEC63	0.045167542	0.231349729	0.557798722
COPG1	0.024820788	0.0923731	0.558814201
FAM129B	0.000450493	0.002698027	0.559492155
SMAD6	0.043572922	0.241335602	0.559856072

HIPK3	0.162698854	0.049912895	0.559917524
TRIB2	0.008851231	0.014416011	0.560073793
TMED5	0.118770235	0.0081329	0.560268664
GLCE	0.071825344	0.024039489	0.560354269
HGSNAT	0.007348252	0.087372782	0.561314428
HSBP1	0.005255683	0.000376418	0.561372796
TP53RK	0.037273187	0.065745845	0.561438268
NXN	0.041625307	0.039550642	0.561773909
ADNP2	0.001557949	0.00236556	0.561800226
SP110	0.00279649	0.024547742	0.561961482
VTA1	0.001236907	0.002392891	0.562182837
RFX7	0.073942893	0.011084449	0.562231061
SLC7A6	0.046745916	0.225200933	0.562395619
GPX3	0.019795089	0.162050169	0.562542753
FPGT	0.068940746	0.002966909	0.562724927
BBC3	0.006330417	1.93249E-05	0.563116796
SART1	0.160046373	0.043792273	0.563780913
YWHAE	0.03687113	0.087072778	0.563839472
MAML3	0.014143238	0.0065947	0.56398486
DMAP1	0.005849365	0.033170365	0.564295201
FAM83B	0.009097041	0.086338469	0.5645498
ATF4	0.004057001	0.016947218	0.564553156
ELK3	0.021118856	0.044012695	0.564715895
C11orf68	0.028179975	0.172064188	0.564743309
CRKL	0.047928137	0.020322609	0.565231975
ZMAT5	0.049750029	0.084242178	0.565499804
ICAM4	0.124218546	0.033126698	0.565642209
STK3	0.092322847	0.034199838	0.566242987
TAX1BP1	0.067287307	0.00996347	0.566450715
ZFYVE27	0.072492973	0.035761067	0.566469516
PRR14	0.030948387	0.132331394	0.567230593
MSX1	0.092690903	0.032962728	0.567257204
PIGBOS1	0.014585357	0.018303918	0.567650177
SLC12A4	0.019220047	0.08382173	0.567832281
USP6NL	0.051331696	0.002546583	0.567900686
PLEC	0.003264476	0.000117715	0.568272304
SDHA	0.008594862	0.001534657	0.568330313
KDM7A	0.034324282	0.156338433	0.568790456
H2AFY	0.01329637	0.036734202	0.56893687
PSMB7	0.005011945	0.015086735	0.569334976
AC106791.1	0.044621377	0.090311952	0.569542136
PGAM5	0.139602517	0.039314192	0.569920025
BCKDK	0.014450194	0.024844116	0.570234899

SLC6A6	0.118379474	0.013606134	0.57058221
C11orf58	0.047347627	0.010541225	0.570838354
PINK1	0.06945527	0.023190116	0.571446468
RINT1	0.011763988	0.071800542	0.571693573
TCOF1	0.129756253	0.047234983	0.572117427
TCEA1	0.135629269	0.026498579	0.572273576
PRPF3	0.024347661	0.008722031	0.572359398
FAM78A	0.040044718	0.147607805	0.572524218
ZNF276	0.037131735	0.210647945	0.572543139
SCAP	0.079811715	0.018783675	0.572674649
SETD1A	0.142704743	0.043153891	0.572883934
COX19	0.011152162	0.002303625	0.573459089
STK24	0.002382554	0.000356629	0.573742336
IRF2BP2	0.115877407	0.03825332	0.573915699
USP45	0.019963975	0.035512775	0.574389396
ZNF614	0.111434308	0.014610826	0.574442852
SPPL3	0.024084521	0.00630969	0.575867791
ANKIB1	0.128845075	0.02929774	0.576065074
MRPL36	0.035000195	0.015254819	0.576260503
ISCA2	0.000603823	0.000985989	0.576321489
GLYR1	0.044595062	0.047438304	0.57637778
TMEM63A	0.003756908	0.005175858	0.576866467
SEMA3B	0.158537876	0.022204031	0.577101227
COX5B	0.020827649	0.049475509	0.577406166
HEXIM1	0.064885407	0.012677007	0.577484794
BMPR2	0.000959528	9.83581E-06	0.577580462
HSPA13	0.008165435	0.022969482	0.577658612
GORASP1	0.02145738	0.0516756	0.578127402
SYVN1	0.033540009	0.141176758	0.578323866
CARD10	0.081670526	0.014216309	0.579251391
HMGN1	0.013515693	0.035098107	0.580391356
AC114760.2	0.043336949	0.225200933	0.580476219
HNRNPR	0.049980592	0.181695977	0.581199405
MAN2B1	0.094734144	0.033499504	0.581542409
RNF220	0.109339813	0.026532272	0.581662375
SLF2	0.032615822	0.006141173	0.582443244
HERPUD2	0.033131762	0.007626381	0.582942345
MUC4	0.013140036	0.002616111	0.583420537
BRMS1	0.038587135	0.095634568	0.583428344
TBC1D32	0.007593145	0.072838478	0.584022227
RBM4	0.004569998	0.01293217	0.584328263
RANGAP1	0.015104128	0.039937712	0.584384601
PET117	0.090264327	0.017861328	0.584386488

KDM3A	0.01592187	0.026613566	0.584993767
NUDCD3	0.008402662	0.015572518	0.585073632
LAMC2	0.024582195	0.045063087	0.585211161
LYPD6	0.012585556	0.043434173	0.586068736
METTL23	0.032493488	0.112856816	0.586129105
RITA1	0.060739525	0.007490142	0.586495786
NKIRAS2	0.000102809	0.00270426	0.586843845
NDUFS1	0.089184433	0.04200784	0.587107184
PODXL	0.148742984	0.036800296	0.587209978
FAR2	0.036035721	0.015424567	0.587263
ANKS1A	0.000105384	0.001962432	0.58733889
RND1	0.045571008	0.101924019	0.587466276
MOB3C	0.241831743	0.034253967	0.588614281
TOMM5	0.048557551	0.149267527	0.588929839
CDK5	0.182290294	0.033507569	0.589116447
GRAMD1B	0.003785923	0.000360843	0.589354476
C3orf38	0.039241046	0.002806047	0.589371633
EML4	0.05064475	0.001464415	0.589432649
PEX19	0.029618059	0.010236295	0.590434068
TRIO	3.33818E-05	9.5582E-05	0.590959526
SRRM1	0.033951053	0.023655259	0.591312316
FAM207A	0.05598276	0.014815898	0.591429559
SF1	0.012267035	0.056357318	0.591655884
AP001160.3	0.169676744	0.033452791	0.591687715
CUL3	0.004315842	0.000978702	0.591973718
KRT10	0.0126274	0.039388123	0.592125025
FAM84B	0.285444424	0.03072349	0.592896724
TYMP	0.012538029	0.029351481	0.593266097
PAIP1	0.060295144	0.031374928	0.593543193
APLP2	0.021290504	0.000203264	0.593836678
MIR22HG	0.018108912	0.006678069	0.593891107
TMC8	0.018266622	0.048198115	0.594382696
P4HB	0.005936615	0.033274176	0.594423642
SEC31A	0.031371926	0.168092091	0.594480527
SHC1	0.035649348	0.002391661	0.594504726
C1orf52	0.008918677	0.065036608	0.594968163
BAP1	0.001585369	0.000529369	0.5954095
ARL14	0.086981693	0.040729887	0.595651081
TGIF1	0.046337665	0.00928952	0.59639823
UBL4A	0.069383825	0.019825965	0.596598839
NUDT9	0.023316124	0.01200109	0.596832663
LGALS3BP	0.033715533	0.002811217	0.596936408
CD63	0.025830252	0.003285907	0.596990444

JARID2	0.014806729	0.163945445	0.597875914
CHMP4C	0.12981065	0.025782384	0.597985143
INAVA	0.004433521	0.000154829	0.598621683
PRF1	0.044965052	0.26087726	0.598659692
NCOA3	0.029306742	0.048825261	0.599969423
PDE4DIP	0.018040204	0.054298107	0.600103328
PPP1R35	0.001876883	0.007288024	0.60020925
NCOA2	0.069428238	0.016597064	0.601011507
BTRC	0.01755169	0.01338603	0.601038363
RBM7	0.030237944	0.002392908	0.601432386
PHB	0.035398074	0.009065861	0.601807488
NAE1	0.003648126	0.06430376	0.601822396
TRPV2	0.010753375	0.014630831	0.602090322
MPHOSPH8	0.040470219	0.142476487	0.602507074
WTAP	0.015017344	0.003972364	0.603010361
PTHLH	0.04077935	0.087110928	0.603750366
CDADC1	0.032696752	0.088548903	0.604285632
LTBP4	0.04186043	0.158271447	0.60448862
TMEM167B	0.017977396	0.121329273	0.604547675
HNRNPF	0.003093483	0.014181878	0.604706205
SEPT11	0.072375268	0.013305743	0.604732141
RSRC1	0.000147541	0.073129363	0.604815738
VPS8	0.075807353	0.014166269	0.604843707
GPAA1	0.011591805	0.004665581	0.604917399
S100A16	0.111148857	0.007277072	0.605175378
RFWD3	0.025710818	0.02425654	0.605574727
SARAF	0.051498543	0.005405209	0.605620939
ADAM28	0.028537835	0.081429214	0.605652418
FBRSL1	0.002225815	0.014469801	0.605816553
SRD5A3	0.030803944	0.087152342	0.606316618
MYO9B	0.022769249	0.046359542	0.606429357
NPC1	0.119943432	0.049570731	0.606595831
ZNF775	0.038225431	0.088890491	0.606953904
PNRC1	0.006077772	0.008392043	0.608297656
DNAJB2	0.096583482	0.048082413	0.608310457
PTP4A2	0.001547334	0.001777204	0.609241148
GGNBP2	0.037022408	0.007784181	0.609586841
NUP85	0.111602771	0.021663824	0.609798848
RNF6	0.006676577	0.005656091	0.610741236
SPRY2	0.024030862	0.006588057	0.611719872
FBRS	0.02411955	0.007611372	0.611927791
C6orf62	0.002846205	0.009751139	0.612056788
TMEM63B	0.118037221	0.019066087	0.612430416

FAM111A	0.049843994	0.070585297	0.612512429
FAM120AOS	0.149451615	0.027062614	0.61252949
RHEB	0.005489463	0.005408921	0.612814605
SMAD7	0.076182472	0.04710897	0.613067499
LMAN2	0.084051564	0.018886989	0.61324768
ING2	0.077089477	0.025120658	0.613272806
DGCR6	0.126976185	0.033570158	0.61333654
RBM23	0.002895209	0.005674312	0.613800608
RP2	0.034510549	0.047411928	0.615055882
KRAS	0.012214357	0.002245114	0.615311682
DNAAF4	0.103004317	0.019149807	0.61559372
NECAP2	0.041832927	0.008949663	0.615627525
C19orf66	0.083976739	0.022133685	0.616790907
GNL1	0.004995435	0.040832084	0.617067839
PRKAG1	0.007381598	0.012701268	0.617105259
SPAG9	0.126034546	0.02413583	0.617827261
NUP93	0.004303855	0.010905563	0.61847747
AL355312.4	0.060422924	0.014815441	0.618677111
PTPRH	0.117703666	0.032412988	0.618742569
ATP6V0B	0.01457343	0.02112261	0.619650794
CARHSP1	0.006929162	0.013521138	0.620111654
NFKBIB	0.009861497	0.031143371	0.620366056
UBE2Z	0.127927464	0.038813648	0.620801371
ALG6	0.104234803	0.031786948	0.621810682
ASB7	0.221041262	0.047907743	0.621877623
MECOM	0.025881043	0.002873074	0.621936137
TTLL12	0.125463471	0.038843611	0.622782043
XRCC4	0.004960284	0.001390706	0.622786582
CCM2	0.000586366	0.000599344	0.623227304
CYLD	0.148055685	0.033341581	0.623744065
C5orf63	0.03310401	0.088651911	0.623971612
IRF7	0.00020596	0.002018704	0.624396187
FEM1B	0.003709477	1.24791E-06	0.624587348
RNF14	0.039251408	0.041945138	0.624737104
SMARCD2	0.009005771	0.006922918	0.62479671
TXN	0.047395108	0.262286921	0.62479887
SLFN11	0.027584651	0.15129284	0.624898886
C1orf43	0.111648097	0.026422174	0.625485997
PTPRE	0.089815863	0.022377044	0.625543647
DPH3	0.014061616	0.00301319	0.625830228
PTOV1	0.008419275	0.034556959	0.625860767
TSPAN5	0.004710974	0.011669839	0.626129053
RHBDL1	0.041031821	0.225200933	0.626419809

TSPAN15	0.002663917	0.001335281	0.626662929
VKORC1L1	0.10371179	0.042915173	0.626785169
FKBP14	0.01362183	0.092763061	0.627626342
NDUFS2	0.00809596	0.001589624	0.627732431
PTK2	0.001384314	0.000100177	0.627766163
CNP	0.008408636	0.000252934	0.628036742
RAPH1	0.031297575	0.005276382	0.628201624
NAAA	0.00537865	0.028036576	0.628302234
CARM1	0.007270586	0.038785424	0.628385551
ARF1	0.014232985	0.002877459	0.629057253
MTMR11	0.003362171	0.015540868	0.629150101
AGRN	0.003188231	0.006607855	0.629213766
NUDC	0.015915721	0.077492108	0.629663717
AMFR	0.031645847	0.005066019	0.629825881
ECE1	0.015405289	0.080393425	0.629906967
ZNF865	0.023841586	0.094311169	0.630094617
LYSMD2	0.009379708	0.080260493	0.630233338
SPHK1	0.084868371	0.006046951	0.630695596
DNAJA1	0.050879737	0.018313849	0.630948045
TRAM1	0.067647136	0.019058572	0.631367342
PLA2G6	0.091735486	0.0309952	0.631427436
TRIB1	0.024766012	0.085327895	0.631555647
CNBP	0.080287224	0.02124647	0.631944774
GLMN	0.029306513	0.089874427	0.632250823
WNK1	0.047519979	0.004257096	0.632569678
ING3	0.107512774	0.048869006	0.632892799
BRD9	0.014983277	0.081756121	0.633237927
ERBB2	0.043588364	0.021110327	0.633326806
SEPHS2	0.000505538	0.000120709	0.6334036
MMP14	0.063680217	0.018641258	0.634004174
M6PR	0.01582252	0.053468002	0.634231231
RPUSD1	0.057619134	0.016222845	0.634522482
SF3B4	0.005669971	0.000407959	0.634544521
AL049597.2	0.044278115	0.034586878	0.635183351
NAIP	0.071873896	0.01024525	0.635307331
ARL4A	0.023449839	0.030800324	0.635414784
CHP1	0.039737766	0.012732667	0.636049249
GAREM1	0.095646863	0.014061287	0.636104156
TCTN2	0.020951204	0.043206628	0.636314501
CNDP2	0.039618969	0.065650006	0.636317398
TXNIP	0.002308402	0.01337183	0.63721427
ZNF777	0.0463542	0.073416342	0.637981973
HIPK1	0.005635697	0.003862163	0.638253098

MT-ND4L	0.002055799	0.004030212	0.638559047
ARMC7	0.035849174	0.141325507	0.638932114
CAPZA1	0.009368563	0.00521333	0.639385453
C9orf72	0.02075427	0.063784147	0.639645752
ARMC6	0.009581582	0.033449351	0.63981303
PKD1	0.040379203	0.085220404	0.64023132
ATAD1	0.059731439	0.00789094	0.640289887
LPCAT3	0.046788414	0.024362555	0.640525748
KIF13B	0.065527869	0.013064656	0.640654501
GOLGA7	0.009591326	0.031162736	0.641003459
OXLD1	0.04855431	0.196338956	0.641205244
NDUFB5	0.006405053	0.007087602	0.641343749
CCL20	0.116362279	0.015203543	0.641721444
XRN2	0.000276121	2.94298E-06	0.641883104
OSGIN2	0.038491888	0.080967835	0.642242927
ENOX2	0.115416631	0.033942246	0.642243575
SQSTM1	0.011140295	0.005078409	0.642638256
C12orf75	0.133374904	0.024554847	0.642652373
SLC6A14	0.006370912	0.000396733	0.643558796
GON4L	0.101625732	0.032557926	0.643597735
TYROBP	0.070423327	0.04325956	0.643629026
FAM214B	0.045397668	0.125931787	0.644248161
AC080037.1	0.092804213	0.005904424	0.644480929
PATJ	0.074026778	0.01218074	0.644734787
ODF2L	0.036858645	0.014501156	0.644820124
MBTPS1	0.0376633	0.089853203	0.645079291
PFDN2	0.003786192	0.006616191	0.645208164
PTK6	0.144682316	0.042058343	0.645708578
SELENOK	0.021727288	0.054872351	0.646477091
SNX5	0.041385338	0.125351481	0.646494799
CDC42SE1	0.006923595	0.01504813	0.646541023
NT5C2	0.024036864	0.01064754	0.646575557
TRIP10	0.009816883	0.026396757	0.646699564
MLPH	0.03489655	0.005670111	0.646927085
PRR12	0.011180514	0.004572862	0.647069125
WDR33	0.003023851	0.002750398	0.647097903
GIGYF2	0.004200633	0.018098834	0.647458893
RFK	0.001157285	8.18015E-05	0.647796967
LMNA	0.007306248	0.001446671	0.64780265
OGA	0.000798775	0.000807396	0.64804854
CANX	0.021946872	0.057947005	0.648406252
CMPK2	0.035951221	0.035121832	0.648840049
RER1	0.074337414	0.019394643	0.649572254

ASB6	0.002495703	0.003051935	0.649595627
RBM4B	0.024046775	0.006522351	0.649715138
MTERF3	0.046842676	0.08294518	0.650221606
RAB40C	0.006982477	0.000357382	0.6503623
PTBP1	0.000961336	0.000116498	0.6505836
SESN1	0.048934653	0.095079336	0.650726394
INF2	0.079205377	0.027877509	0.650728509
GATD1	0.045741207	0.147288121	0.651043376
RDH13	0.164044133	0.043837525	0.651080322
SUPT6H	0.065623848	0.048247346	0.651179224
TMEM218	0.027026006	0.048737234	0.651320816
SKP1	0.044256432	0.002060381	0.65187324
NRIP1	0.004671003	0.004550554	0.652093829
ATP5ME	0.042861078	0.063407006	0.652219302
ZNF358	0.025863472	0.147734773	0.654050927
MALAT1	0.025091595	0.062076018	0.654866555
ZNF148	0.083802821	0.037177102	0.655075497
FOXN3	0.007836806	0.05083759	0.65528376
HGS	0.031774557	0.052753068	0.655372119
ATG101	0.030433229	0.003340923	0.656005498
SLC38A2	0.031387592	0.044883354	0.656353618
PHC3	0.161048843	0.042839498	0.657009223
EIF5	0.007195844	0.004853521	0.657329027
SMAD3	0.000214431	0.000525497	0.657613456
CSGALNACT1	0.068372562	0.037228159	0.658460806
AMN1	0.009541702	0.024522499	0.658750807
TBRG1	0.002263614	0.018620056	0.658965183
RGS12	0.061240039	0.011760153	0.659064949
IER3IP1	0.028658499	0.01016708	0.659335687
EXOC3-AS1	0.023096462	0.014526264	0.659662741
BARD1	0.004651899	0.013622175	0.659744606
APMAP	0.05749567	0.039885346	0.659818328
CTCF	0.057654508	0.017838177	0.659911077
PLSCR1	0.008245151	0.000926435	0.659984337
TLE1	0.084741553	0.02107138	0.660985001
SHTN1	0.032271448	0.01883358	0.660998066
ERCC6	0.047129811	0.099770545	0.661074463
FOXL1	0.039633138	0.015515307	0.66130243
MRPL58	0.000118288	0.00154121	0.66162518
UBE2D3	0.008173517	0.00264054	0.661979142
CPSF4	0.018533135	0.010020322	0.662391358
SYNE2	0.017676293	0.011751066	0.662462685
LRPAP1	0.044314481	0.005447044	0.662902567

RAB21	0.001779083	0.014047033	0.663143098
MRPL14	0.042411803	0.007514179	0.663698778
FBXO42	0.011581932	0.043044702	0.663785497
CYP3A5	0.073126421	0.002106142	0.66379462
ADGRE5	0.000805805	0.003701777	0.663905369
USB1	0.00184823	2.32176E-05	0.664107901
TMCC3	0.043798251	0.140065048	0.664138087
PCMTD1	0.000950049	0.001738747	0.664298346
CCDC137	0.021684654	0.045893438	0.664711763
OSBPL1A	0.133962543	0.040169343	0.665245697
CASP1	0.028979514	0.136784319	0.665310936
CNIH4	0.042573238	0.091562103	0.665452175
SLC44A3	0.042951569	0.109314715	0.666503219
SVIL	0.009880353	0.000744657	0.666533717
PRR14L	7.98983E-05	0.000151766	0.66669289
REV1	0.094320184	0.049053682	0.666788024
LAMA3	0.003305238	0.000728713	0.666821853
CPSF7	0.034746982	0.15805174	0.667017497
TIPARP-AS1	0.04084604	0.225200933	0.668011699
EVA1C	0.022205835	0.09448299	0.668470039
BAZ2B	0.113577168	0.035340148	0.668475895
DDX5	0.000237853	0.000643108	0.668924895
PDXK	0.016485541	0.047148368	0.669183109
PCBP1	0.012832144	0.001537062	0.670184087
TBC1D2B	0.03774382	0.086030348	0.671820258
KLF13	0.025049843	0.034807313	0.671895143
MAPK14	0.000882969	0.007733996	0.672276011
HIST1H2AE	0.022166661	0.039274149	0.673054925
AK4	0.024079969	0.16049007	0.67412389
ZPR1	0.026272012	0.018847108	0.674124547
VNN1	0.01105662	0.033708042	0.674253203
PTK2B	0.002442155	0.056604828	0.674981173
ARHGEF5	0.029350828	0.018682358	0.67530821
TMTC3	0.004444782	0.015098965	0.67580252
UQCRFS1	0.021715915	0.05186894	0.676628124
TUBA1C	0.006508392	0.000422006	0.677735249
R3HCC1L	0.064419895	0.03268321	0.677880354
ATL2	0.016625427	0.00193373	0.678336779
RUFY3	0.001066068	0.003601403	0.678609239
MTERF2	0.239003352	0.033036475	0.678641821
CDC5L	0.000749975	0.000768554	0.678718565
AES	0.02610831	0.001690797	0.679292592
SPEN	0.017714702	0.033394279	0.679470142

S100A3	0.038996911	0.104225562	0.679593162
NDFIP1	0.04139245	0.013731528	0.679706409
AP001157.1	0.019066434	0.03964072	0.681010134
RARB	0.137701004	0.029149322	0.681423571
ATF2	0.042879048	0.012348549	0.682371335
LTA4H	0.00066948	0.099012886	0.682632881
DDT	0.060661921	0.012327163	0.682818112
GMEB1	0.040770539	0.115225326	0.683469239
CIR1	0.004837011	0.00142731	0.683652726
ARFIP2	0.008223789	0.013362269	0.683735281
SLC38A7	0.006015049	0.016400121	0.683820182
CDV3	0.007568376	0.014842195	0.684245326
MAPK13	0.094061294	0.026689304	0.684586105
PGRMC1	0.082080596	0.017790036	0.684597586
TMPRSS3	0.018929437	0.018760612	0.685717393
MAPKAPK5	0.00081833	0.00155782	0.685865343
GAN	0.007876189	0.005736085	0.686989632
PARG	0.011913972	0.025072098	0.687468816
YIPF6	0.028785122	0.012458115	0.688996289
FBXO32	0.041524405	0.01172717	0.690029054
YBX3	0.01691565	0.071353192	0.690052487
ACP5	0.06847225	0.045867228	0.690606718
ARAP1	0.020119327	0.041309527	0.690654602
SERPINB1	0.027419541	0.013587903	0.691167932
PDZK1IP1	0.025574548	0.006814822	0.691334303
CEP112	0.101029963	0.034593566	0.691376969
MVB12A	0.003921168	0.025987444	0.691509901
GOLGB1	0.018872378	0.032721555	0.692423381
ITGB4	0.004262568	0.001481985	0.692553983
UBE2R2	0.096369931	0.042867683	0.692672017
KLK10	0.099491324	0.001174931	0.692926335
TMC6	0.101777339	0.049983183	0.693545186
FOXP1	0.027011332	0.023561917	0.694746623
RNF126	0.069834277	0.003619737	0.695374371
MYNN	0.084986495	0.03269531	0.695912295
DLGAP4	0.045274597	0.12020492	0.696000002
HMGA1	0.015302501	0.010162324	0.696216187
MAP3K8	0.031862349	0.002459488	0.69660108
MSI2	0.002433128	0.000425129	0.696848119
RAB5A	0.078193351	0.026545309	0.697371499
TXNDC12	0.030414573	0.019434971	0.69762623
EXT2	0.020824691	0.042200698	0.697804059
FBXO11	0.045672526	0.137220759	0.698451532

ATG5	0.021758438	0.093540077	0.698572158
GBP4	0.02714344	0.002100397	0.699998465
TNKS2	0.000297202	2.37233E-05	0.700155703
EIF1	0.010935849	0.012453256	0.700393314
ARGLU1	0.045500562	0.120651553	0.700463036
ETFRF1	0.014566559	0.005969106	0.700476342
NCBP2-AS2	0.025588986	0.038518394	0.700567593
NDUFA6	0.011098675	0.017642555	0.700810798
CASP10	0.039274427	0.02243468	0.701220208
UBE2G2	0.048001747	0.007571489	0.701466995
SERPINB9P1	0.00688366	0.005507837	0.701553507
INTS4	0.04004953	0.138200594	0.701647974
PNPLA6	0.16251233	0.008925091	0.701767685
GEMIN8	0.045517034	0.161765386	0.70183788
C1R	0.02354461	0.0293215	0.701972154
LIG3	0.028471547	0.099989223	0.702942244
TROVE2	0.014682097	0.002814908	0.703052426
FIG4	0.01507288	0.009133709	0.70308739
MED1	0.026638323	0.053722535	0.704049376
LSM12	0.014435836	0.005492221	0.704124249
NPLOC4	0.004317772	0.000360686	0.704344644
TRIM47	0.048701637	0.087669428	0.705182884
RPL13A	0.090389657	0.037622182	0.705275338
ILF3-DT	0.002510304	0.000266312	0.705340183
ZNF587	0.017260834	0.016508883	0.705555079
LHPP	0.112767412	0.047696221	0.706256768
TRAPPC10	0.00661733	0.011671416	0.707807337
ITGA2	0.011888862	0.016021586	0.708093972
TNFRSF10A	0.010573903	0.003713063	0.708307294
CLTB	0.115270433	0.035476314	0.709300662
RMDN2	0.045187947	0.083391044	0.709411732
UBE2V1	0.047527628	0.016066339	0.709427641
AFF4	0.001910032	0.003608219	0.709995024
ZBTB38	0.019531854	0.004241009	0.710885784
RALGAPB	0.008374198	0.0040147	0.711011209
MLLT1	0.011512195	0.064413219	0.711193211
LGMN	0.006932093	0.006252459	0.711701385
WDR45B	0.003399944	0.000678777	0.711838535
DHX8	0.041853618	0.015834407	0.712405698
S100A10	0.02038061	0.016096458	0.712605633
ZKSCAN1	0.005490527	0.023097912	0.712629811
GPAT4	0.00205565	0.011490174	0.712712443
OAS1	0.022972489	0.016796471	0.713052803

C16orf58	0.090826756	0.020429179	0.713053298
SLC17A9	0.131139954	0.025255907	0.713539002
ELOVL7	0.038565262	0.037681762	0.713664178
ZFP91	0.015359316	0.00211764	0.713709552
SUPT5H	0.009500065	0.012000288	0.714039423
ZSWIM4	0.001655211	0.00129029	0.714470141
ZFAND5	0.005525005	0.001256344	0.714571103
ZFY-AS1	0.03041801	0.034105819	0.715326441
OTUD4	0.004077003	0.002814183	0.715406223
CD59	0.000731913	0.000170639	0.715746395
SLU7	0.001750371	0.005239002	0.716153835
MED19	0.067640117	0.037264975	0.716226552
RBM5	0.028196371	0.024446215	0.716631716
ANKEF1	0.099766262	0.037061553	0.716762891
ZNF292	0.000683127	0.007157353	0.716794934
KHDRBS3	0.049507824	0.029240836	0.716933142
TEN1	0.009091419	0.001855726	0.716980489
NAMPT	0.011951096	0.005057014	0.717203028
AHDC1	0.004537946	0.0057836	0.71762158
CDKN1A	0.000201201	3.69309E-05	0.717717205
ZCCHC2	0.014641084	0.006436341	0.717826327
HS3ST1	0.022077721	0.000831843	0.717845552
AP1B1	0.012031236	0.004675674	0.719053396
PLAUR	0.000457013	1.00427E-05	0.719115305
KCTD10	0.045010695	0.128193214	0.719502373
FAM96B	0.038874981	0.028979346	0.719601502
MYH14	0.00033654	5.35792E-05	0.719893239
CGAS	0.003224091	0.003967258	0.720004076
ESPN	0.066153965	0.041263778	0.72007192
RIPK3	0.005899443	0.020120639	0.720429567
PRRG4	0.010878236	0.036785092	0.720572459
DDX56	0.001275621	8.04964E-05	0.720860882
MYH9	0.029240556	0.009427092	0.720993499
MFSD5	0.001130553	0.00012243	0.721162316
TBC1D9	0.102297796	0.038847133	0.721227839
ARHGAP21	0.036156991	0.011132638	0.721231474
AFAP1-AS1	0.009235192	0.003394865	0.721995623
TUT4	0.030525588	0.026648712	0.72207888
SAP30	0.000875545	0.001651551	0.722184307
MRPL30	0.042040518	0.007570945	0.722925054
RB1CC1	0.008260809	0.083696215	0.72321326
MIF4GD	0.026046433	0.227010615	0.72351893
IER5	0.092931971	0.018454519	0.723696352

OTUD5	0.0029512	5.93467E-05	0.723936557
KTN1	0.01150286	0.004188494	0.723969289
PLEKHB2	0.003159724	0.006293777	0.724100926
FAF2	0.014214524	0.025317964	0.724166232
SHLD1	0.03368413	0.033992426	0.7243587
CAP1	0.003208704	0.000141347	0.724462485
PXYLP1	0.040291118	0.225200933	0.724934289
ATG3	0.287707926	0.042251718	0.72607238
RAB7A	9.31632E-05	7.17113E-06	0.726163869
SHISA5	0.016502491	0.041812617	0.727453543
NT5E	0.024598972	0.00223827	0.727457875
NAPRT	0.026600715	0.001648192	0.72768445
ZNF500	0.092807058	0.041331676	0.728152037
ARF3	0.074146335	0.038945327	0.728159542
CAMK2G	0.006416991	0.03033763	0.728315659
VPS28	0.001138917	9.58191E-05	0.728422824
PDE12	0.048717584	0.089813661	0.728739242
IPCEF1	0.039625764	0.240940913	0.729355465
PGPEP1	0.113996108	0.016860557	0.729718516
CDK7	0.013463717	0.004345895	0.729942248
NORAD	0.052522715	0.020573494	0.730559039
ATMIN	0.166890458	0.028349699	0.731046193
EN2	0.23331272	0.034828542	0.731299326
BIRC2	0.058663524	0.04371617	0.731314862
AFDN	0.032577307	0.010304414	0.732483915
DOK4	0.010253483	0.011779498	0.732895924
CHD8	0.00130375	0.000719305	0.733235835
TGOLN2	0.032859019	0.011384951	0.733929133
CLUH	0.00132162	0.012401148	0.73422139
SREK1IP1	0.083097666	0.036614442	0.734515384
CHURC1	0.068602193	0.021385637	0.734636275
RNF38	0.008358536	0.002669853	0.735127593
NEU1	0.005523887	0.009461743	0.736591186
LAPTM4A	0.007670733	0.017073932	0.73665505
FAM210A	0.003554137	0.002612876	0.73777039
THRAP3	0.0299535	0.044131791	0.738282148
GRPEL1	0.01767735	0.006044674	0.738459815
ZNF207	0.012053442	0.006156252	0.739355
TSNAX	0.01042865	0.003376725	0.739587041
KIF3A	0.199626649	0.036027881	0.739827499
TNFRSF14	0.000871405	0.004000722	0.740017493
FUCA1	0.011315492	0.033719795	0.740039217
RYK	0.127208588	0.046267587	0.740088908

HTATSF1	0.089045921	0.031561621	0.740517023
LCAT	0.031758192	0.019049289	0.740610903
ANTXR2	0.005900974	0.007598471	0.741096328
ALPK1	0.021580925	0.017494602	0.741513265
COX7A2	0.009775663	0.000331013	0.741698033
N4BP1	0.01149824	0.010579206	0.741874677
ATP10B	0.006737541	0.005679092	0.742764675
HIP1R	0.017955997	0.005393164	0.743421695
SNRNP27	0.080587652	0.017518226	0.744020712
MXI1	0.011032752	0.009278732	0.74442824
FAM135A	0.000487875	0.007193008	0.745343202
ATP2A2	0.048538583	0.020632494	0.745814088
OGFR	0.060353088	0.0298179	0.745840508
FOXA2	0.110425569	0.017711084	0.746867807
AP1M2	0.087338616	0.034887986	0.74753171
UNC50	0.031759876	0.016445472	0.748290545
FAM3A	0.084436255	0.040610811	0.748485185
TIMM23	0.010986264	0.010987674	0.749101079
IKZF1	0.058136239	0.004073728	0.749165614
KLK8	0.099086971	0.042891487	0.749783177
NMI	8.33009E-05	6.274E-05	0.751073256
ATP5MD	0.025412565	0.009513185	0.75174382
TPD52	0.071747905	0.042513285	0.752201697
GPN2	0.034182706	0.011215465	0.752351864
ANKRD13D	0.043459565	0.112357315	0.752379716
APOL6	0.004042814	0.003411946	0.752520757
MAFK	0.120198296	0.042099277	0.752689541
SEMA4C	0.022956362	0.097468612	0.753516253
AKNA	0.001084332	0.000919192	0.754068083
OTULIN	0.005175663	0.004033261	0.755546921
TMCO3	0.003016428	0.000339507	0.755580049
USP40	0.020185308	0.008585538	0.755814387
MIGA2	0.041732226	0.013653946	0.756033112
APOL1	0.003262793	0.000255572	0.756478049
MICU2	0.000534365	0.008711437	0.756801735
COX8A	0.193169647	0.047834751	0.757756367
FEM1C	0.095584215	0.017226792	0.758883975
PPIG	0.034156931	0.060931764	0.759126263
SPTLC2	0.057644185	0.025473588	0.759548912
HYAL1	0.022451613	0.189275335	0.759731233
AC016065.1	0.051395131	0.04077816	0.760162465
PLGRKT	0.009976706	0.024623844	0.760246699
NFKBIZ	0.002859031	0.00351454	0.760522052

PSMG2	0.014870066	0.027384423	0.76077753
GABPB1	0.001183709	0.005089126	0.760941196
TSPAN14	0.006125805	0.0063475	0.760976927
HMG20A	0.000117796	0.000951615	0.761093462
MEF2A	0.000715888	0.002734681	0.761410724
RCE1	0.00685597	0.002186628	0.76148371
SNX1	0.026374074	0.015380687	0.761503432
QDPR	0.008406513	0.038283472	0.761786493
VWA1	0.04527715	0.016229501	0.762109247
RPL36AL	0.050897728	0.049219252	0.762203785
PTGR1	0.027285819	0.014098122	0.76284862
TIMP1	0.000389403	0.000271937	0.763400119
SEMA7A	0.03393048	0.045608321	0.763819438
TMEM14A	0.089250019	0.032563202	0.76385501
FDPS	0.081150285	0.025432039	0.764223649
GHITM	0.003884953	0.001398162	0.764277298
THSD4	0.039736621	0.103199088	0.764588533
CDS2	0.001136084	0.00777636	0.764710846
ENAH	0.056280392	0.042957669	0.76471984
GPN3	0.079913733	0.046843886	0.765289263
PCMT1	0.009043586	0.002847366	0.765400127
ATP6V0E1	0.029002049	0.036627938	0.765681557
AC020978.5	0.095317905	0.014948732	0.766283113
ZNF608	0.022126309	0.01013844	0.76676248
TATDN2	0.001791702	0.038764306	0.766831197
RC3H1	0.002302486	0.005006383	0.766979174
ZG16B	0.003938576	0.010518225	0.768151385
NECTIN3	0.033593233	0.006016848	0.769011824
SMC6	0.009843238	0.007309483	0.7697284
CCPG1	0.043821346	0.127618546	0.769800367
PLBD1	0.010362888	0.004272311	0.770174333
ERO1A	0.000742709	1.78417E-05	0.770517414
MYO1C	5.27371E-06	0.000384841	0.770586747
IFT22	0.07161931	0.030823882	0.771363012
MTMR14	0.019529929	0.03405011	0.772185772
OSBPL3	0.020574779	0.009425562	0.772961622
YKT6	0.004340612	0.002060712	0.773152519
CCDC107	0.039390101	0.021575552	0.773344777
LMF2	0.00014131	0.000178766	0.773673455
BOK	0.018918244	0.022456104	0.773833691
ELOVL1	0.048194009	0.015065343	0.774684911
AACS	0.047107179	0.02462348	0.775016136
NDOR1	0.181571994	0.011861118	0.775395408

USF1	0.026371156	0.024816028	0.775906212
SLC38A6	0.00741064	0.01984279	0.776377896
MACF1	0.016257645	0.034077704	0.776655598
DENND6A	0.002298226	7.09075E-05	0.776655987
PSMD14	0.00421558	0.028855394	0.776734523
AFTPH	0.053261012	0.0289568	0.77746366
FAM208A	0.003277498	0.006942135	0.778747467
BCL2L15	0.004932028	0.001422406	0.77890029
ASXL2	0.002679497	5.74294E-05	0.779128099
ARPC5	0.0205248	0.0141419	0.77926217
UROS	0.000615285	0.000119157	0.77958611
ZNF410	0.040291118	0.139648087	0.780736738
PTPN12	0.011375177	0.009562691	0.780883408
MIR222HG	0.019959778	0.015833363	0.781327739
DNAJC8	0.034580483	0.018038569	0.782127594
WDR1	0.019663834	0.014034073	0.782334867
LINS1	0.002698065	0.000933882	0.782449692
MED8	0.028035202	0.071549131	0.782800006
ZNF574	0.049660502	0.032637615	0.782837277
SYNC	0.081378223	0.037764445	0.784431508
MISP3	0.091423268	0.032227771	0.784457729
EEF1E1	0.030118114	0.036591417	0.784976149
MAGED2	0.022239881	0.026474013	0.78536822
DDX59	0.063891153	0.03908787	0.7856865
KIF1B	0.029973112	0.041548845	0.785989113
FUBP1	0.010054038	0.002961247	0.786102332
ATP6AP1	0.010347071	0.006759029	0.78619807
ZBTB41	0.031191037	0.098887971	0.786228286
SERINC3	0.009030597	0.011816799	0.786669426
TMUB1	0.021931924	0.006357019	0.787405368
GID8	0.011773542	0.015820924	0.787889062
CERK	0.014726531	0.029915084	0.787958435
ABLIM3	0.028519037	0.039359072	0.788259647
PPFIBP2	0.059998875	0.021944117	0.788518881
LRRC14	0.046865056	0.015783377	0.788542371
LAMB3	0.010295971	0.009404174	0.790207885
SCAMP1	0.093492986	0.04505982	0.790429756
TFB1M	0.009283707	0.013087166	0.79044013
SLC35A2	0.028035107	0.014821754	0.790569453
IL12RB1	0.036830228	0.067762526	0.790748927
GLMP	0.00117917	3.59317E-05	0.790962835
DDX3X	0.048731557	0.033583526	0.79104464
TRIM11	0.008103048	0.003303344	0.791061922

PDHX	0.089657816	0.044816531	0.791218939
RIT1	0.013208102	0.130467251	0.791295919
BAIAP2-DT	0.018734677	0.01172179	0.791719806
PLEKHA1	0.116475781	0.026590387	0.791994986
RNF157	0.041002621	0.225200933	0.792232516
HS6ST1	0.007523549	0.004392908	0.792362745
KLF10	0.064376165	0.015463134	0.793613663
ELF2	2.58034E-05	7.02319E-06	0.7937023
HECTD4	0.00480746	0.007676202	0.794349076
RARS	0.027651018	0.012508837	0.794478472
HNRNPA0	0.005245324	0.000557917	0.794532595
CHD6	0.03509563	0.076332438	0.794603404
SF3B1	0.027323446	0.016531984	0.794999499
ZNF337	0.107477277	0.033965502	0.795300276
TMCO1	0.006632379	0.003458959	0.795602476
NEPRO	0.042439997	0.083090496	0.796218405
ZNF571	0.045234882	0.034307548	0.796541263
HIPK2	0.043487664	0.132160428	0.796629362
NSMAF	0.10050145	0.007734106	0.79729108
MT-ND5	0.000391247	0.000202847	0.797428632
ITSN1	0.012366375	0.003364301	0.798127099
ATP6V1A	0.027001697	0.005322725	0.798152864
RPP25	0.107658343	0.012674433	0.798173044
INSIG2	0.017244043	0.02981653	0.798392259
HS3ST3B1	0.046602543	0.225200933	0.798424302
KRT17	0.012188991	0.019103484	0.79903771
SINHCAF	0.002814047	0.014347097	0.799869848
ZNF668	0.083942568	0.010680942	0.800256123
TRPM4	0.052116464	0.008954851	0.800261872
FOCAD	0.009993048	0.029830108	0.800647192
DPP9	0.040078023	0.021135336	0.801297701
DDX42	0.008164491	0.008800637	0.80223014
MRPS22	0.042837404	0.005651373	0.802333394
HSD3B7	0.004461614	0.003616003	0.802367695
NDUFV2	0.002747529	0.000700489	0.802483331
RBSN	0.041374576	0.049507206	0.803436797
PDIA3	0.025184244	0.020563037	0.803974666
HSPBP1	0.042810249	0.056699406	0.804567283
TIMM17B	0.024926196	0.010613028	0.804780663
RAB1B	0.000921169	0.001419784	0.805879293
SAR1A	0.042933124	0.005607552	0.806368636
TMEM267	0.050230561	0.032051111	0.806660267
CHCHD1	0.080599179	0.039507757	0.807519041

ARSK	0.095728594	0.033309524	0.807599804
PTGFRN	0.003181988	0.018650493	0.807859365
ACOT9	0.018780082	0.035725887	0.807970847
NBAS	0.048688627	0.250110237	0.808695407
STMP1	0.009651846	0.013654306	0.809024947
EXOSC7	0.029505462	0.017844727	0.809534085
HNRNPUL1	0.07674295	0.044726661	0.809742116
TRIM8	0.003424279	0.00741313	0.811702195
NFKBID	0.090495213	0.028219711	0.811930984
TMEM65	0.077514353	0.014512354	0.812422958
GLTPD2	0.05446098	0.047192018	0.812992495
SDF4	0.000232092	8.97609E-06	0.813549293
CAPRIN2	0.193580546	0.006811906	0.814473518
FBXO7	0.054867265	0.025536512	0.814640379
PAK2	0.001705593	0.001903185	0.815668131
DHX32	0.016405082	0.003583405	0.81572828
ZNF408	0.011264296	0.004803764	0.815945759
LNPEP	0.015383064	0.008451353	0.816271274
CCDC66	0.138985971	0.039551496	0.816303867
SH3BP5L	0.041037929	0.044426147	0.816728637
MAP4K4	0.000478998	0.000677031	0.816778851
RAB11FIP4	0.008251776	0.001322976	0.816980389
PLEKHA8	0.027790798	0.074594402	0.818158887
NLK	0.008570017	0.005808726	0.818166605
MAP3K13	0.018977546	0.00698072	0.818267139
REEP3	0.017746441	0.006545034	0.818384349
ADAR	4.58841E-05	6.12643E-06	0.8188802
RAF1	0.018242994	0.01546923	0.819094023
FIGNL1	0.055189037	0.021238567	0.819163865
LPP	2.02188E-05	0.000224195	0.819172529
NBN	0.019278538	0.029901783	0.819335226
IL2RG	0.033677831	0.0264945	0.819703121
PHF10	0.111217511	0.026018608	0.820132643
CNOT6	0.007987405	0.033409258	0.820450328
BTBD6	0.043580182	0.046002494	0.820901043
TNRC6B	0.012734634	0.023821185	0.821669644
APEH	0.049348622	0.031344665	0.821855446
CCDC47	0.018899137	0.016718944	0.822051406
SFT2D3	0.003128011	4.20253E-05	0.822187477
SIL1	4.31262E-05	0.000357805	0.822325857
C1orf131	0.004537489	0.003365386	0.822826746
CTDSPL2	0.003462389	0.010612435	0.823921505
KLHL21	0.05392179	0.04608117	0.824080322

MED9	0.040487982	0.035335691	0.824095719
TPRG1L	0.0077649	0.007119853	0.82437976
ARMT1	0.022686992	0.002931359	0.824466176
ENTPD4	0.044099907	0.092130604	0.825328438
CUL1	0.042217966	0.023454846	0.825720903
UBXN8	0.01585664	0.096805426	0.825781984
AC058791.1	0.096786338	0.045082921	0.82578703
ESAM	0.046881596	0.014940177	0.827092348
DENND5B	0.005526804	0.027933545	0.827938629
ELAC2	0.023511457	0.047152987	0.828134729
TCTEX1D4	0.044940073	0.046226933	0.828704766
COL9A2	0.016712904	0.012834262	0.829317353
HIF1A	0.003099038	0.000999315	0.829516059
N4BP2L2	0.013143899	0.010958525	0.829992615
TNS3	0.071282587	0.028041381	0.83007257
CSNK1G1	0.023643643	0.003467568	0.83097968
ONECUT3	0.066463669	0.019340991	0.831032214
PHLPP2	0.096298499	0.034966607	0.831322003
ZNF789	0.215194124	0.03750847	0.831397078
SSR1	0.019482726	0.027318744	0.831621081
ABRAXAS2	0.03033092	0.038878558	0.831769177
LRP12	0.239003352	0.035805983	0.831813121
UCKL1	0.00620352	0.012802103	0.83194132
EPAS1	0.02793867	0.020177337	0.832131781
AL450998.2	0.022063752	0.002760566	0.832266397
GFPT1	0.018434452	0.001698878	0.832321507
SLC25A28	0.013894642	0.013218392	0.832443032
EPS15L1	0.015026653	0.023726663	0.833400768
MSRB1	0.025701754	0.023220067	0.833445944
FOXD2	0.094893311	0.044179871	0.835865011
SMIM29	0.037022805	0.03366951	0.835900296
CTU1	0.001584158	0.00618786	0.836522578
IL6ST	0.025192766	0.020918061	0.836945403
METTL13	0.00596319	0.018872792	0.83725046
KYAT3	0.058072734	0.012266164	0.83826479
MMGT1	0.006233624	0.003347861	0.838809404
GCC2	0.009308032	0.003548314	0.838906052
SH2D4A	0.001805437	0.000117276	0.839217609
ACSL5	0.000423409	0.000153758	0.839472469
TFE3	0.008031872	0.005384174	0.840505539
QRSL1	0.053362957	0.028885547	0.840754378
LGALS8	0.003037867	0.003416732	0.841030789
EIPR1	0.048067268	0.047829777	0.841108159

FBXO21	0.040920793	0.086892641	0.8413698
CTBS	0.040965414	0.025836966	0.84164684
KIAA1217	0.001799877	0.000390153	0.842093617
PNRC2	0.025534271	0.012893938	0.842260569
MCM3AP	0.014246042	0.017967367	0.844585118
FBXW7	0.02851651	0.011501937	0.844587641
CDR2	0.004081765	0.004135951	0.844969485
SYTL4	0.007900944	0.001393066	0.845064131
C1D	0.080789415	0.044268782	0.845268083
SECISBP2L	0.042839235	0.035579957	0.846626985
PAK4	0.022369323	0.0087597	0.847066428
SMIM31	0.024333452	0.009667156	0.847626104
MAP4	0.027681198	0.048559588	0.848092773
DDX41	0.014565454	0.010698792	0.848203833
TMSB10	0.036592257	0.007186683	0.848645585
FOXO3	0.005660168	0.001509118	0.848891728
GFM1	0.021454893	0.008775102	0.849500718
CYB561	0.016889664	0.034796653	0.850338857
SRP14	0.005050569	0.000971308	0.850507562
PYROXD1	0.022778836	0.063528777	0.85065051
TMEM187	0.031718745	0.034770766	0.85072977
BZW2	0.018736478	0.02276002	0.851469878
LRRC56	0.054837683	0.031770359	0.851566992
IFIT3	0.008603751	0.000472383	0.851771777
PLEKHM2	0.029559585	0.024831651	0.851950857
TBCCD1	0.049455907	0.082979484	0.852200068
MOB1A	0.012733459	0.002706113	0.852554045
LPIN1	0.043145861	0.027058026	0.852826967
GTF2B	0.016822087	0.016076492	0.853166011
GGA1	0.002760779	0.004798893	0.853260829
TMEM120A	0.024399559	0.007255391	0.854845763
CUL2	0.014109448	0.018252628	0.854907396
TMX1	0.013223774	0.005344472	0.85530728
DCTN6	0.046130787	0.018104261	0.856064721
EIF2B1	0.012329616	0.013943523	0.856255171
STK38	0.062593383	0.045308233	0.856598139
CSNK1D	0.069217359	0.028777247	0.856662701
TTC13	0.027934829	0.087657242	0.857636629
ANKLE2	0.029271937	0.00423414	0.858117171
UTP4	0.084588491	0.036984353	0.858238842
SOCS3	0.00783243	0.004197207	0.859772372
TNFAIP3	0.002379558	0.00177293	0.860011001
TBX15	0.04678759	0.043164994	0.860665837

RTRAF	0.043806934	0.021605888	0.861120586
LYRM2	0.029423781	0.04657634	0.861177408
IL23A	0.028862603	0.021371434	0.86131475
UBE2K	0.00015646	0.000139779	0.861533422
RPS27	0.026919641	0.02090878	0.861665633
IFT52	0.011498177	0.01036617	0.862006699
ARMCX3	0.01921373	0.032356329	0.862636408
IRGQ	0.02796193	0.037584364	0.862879498
NSD3	0.002457907	0.000382595	0.862939748
HDDC3	0.074640871	0.039777684	0.863153017
CEMIP2	0.00070144	0.000247259	0.863486027
NOC2L	0.039342333	0.023318915	0.864707579
ATP2C1	0.025011053	0.030327155	0.865062344
TAPBPL	0.007407136	0.013084309	0.865083927
LRIG1	0.022887359	0.011414009	0.86513134
SRP19	0.046740047	0.04052343	0.865192678
FUS	0.007809838	0.010679204	0.865658288
WDR73	0.022776995	0.018043447	0.865731032
PPP1R15B	0.040454581	0.0805694	0.866257579
ZNF33B	0.017949779	0.00660148	0.866599543
PIGG	0.023911238	0.020772269	0.866784339
PQLC3	0.035642754	0.03877284	0.866885066
CDC42EP2	0.022720357	0.006794926	0.867054571
C4orf19	0.028684052	0.01182883	0.867078665
CSNK1G2	0.00379295	0.003270055	0.867146623
CXorf40B	0.04049688	0.039351533	0.867373762
ORMDL3	0.032076112	0.00209323	0.867818603
SLC49A3	0.007515151	0.00210133	0.867969879
ACTR2	0.020932158	0.009896206	0.869383666
USP33	0.007468256	0.005359502	0.870377771
SPRTN	0.031376835	0.011663932	0.870595995
MAGED1	0.048394474	0.212449905	0.870975485
LAMP1	0.058375388	0.049574769	0.87108582
SEPT10	0.055291292	0.03924803	0.871786111
ZNF800	0.019550541	0.027501665	0.87196798
DDX19B	0.000425924	0.01277092	0.872636898
ZBTB34	0.048084858	0.052635241	0.872881323
RNF44	0.002667168	0.005042918	0.873279603
LGALS3	0.01659196	0.009822345	0.873466459
DBR1	0.081842479	0.017447407	0.873688626
EMC10	0.015213274	0.009459925	0.87372245
STRN3	0.019293266	0.015790301	0.874343464
EAPP	0.000649387	0.000127667	0.875146435

RBPJ	0.016091536	0.026965709	0.875192865
NMD3	0.022051548	0.025021	0.875260563
MAP3K9	0.035196403	0.092663426	0.875463179
IER3	0.004096365	0.012078159	0.875554525
CPTP	0.126938974	0.041501202	0.875590241
RTN4	0.001764593	0.001044741	0.875932106
XPO4	0.074373385	0.005662621	0.876824675
ELOB	0.024912479	0.012167781	0.876902923
ARV1	0.036581329	0.051391985	0.877042822
CLOCK	0.021939451	0.011844585	0.877556147
MAU2	0.011923355	0.001151423	0.877589547
BLOC1S3	0.177564	0.034600168	0.877738645
FAM53C	0.005917146	0.002228875	0.877925856
CPM	0.009294358	0.02976267	0.878060365
KIZ	0.029739834	0.039393046	0.878551173
BRCA2	0.042994299	0.029654533	0.878782118
CASK	0.024736268	0.029562313	0.879776297
MBOAT7	0.024892922	0.027465615	0.879962696
PLAGL1	0.002472478	0.000344591	0.880042399
AIDA	0.003201706	0.000257494	0.880209729
IGSF8	0.022241756	0.022339642	0.880309442
UGP2	0.019991648	0.01979542	0.882021855
ZFYVE21	0.000503166	3.25043E-05	0.88212067
ATP6AP2	0.034068028	0.038688719	0.882306962
CDC23	0.007062478	0.068167742	0.882535509
SUN1	0.079660536	0.038199481	0.882677485
MAPK6	0.012758723	0.009006891	0.882856147
TAOK1	0.00596626	0.003006679	0.882950577
NAA25	0.039352052	0.05157767	0.884146339
MXD1	0.000920513	0.000119275	0.884333638
CTPS2	0.008154518	0.01994273	0.884380427
CCR4	0.146358577	0.033346046	0.884640139
ZBTB44	0.00291966	0.00594658	0.885073651
HMCN2	0.081338102	0.016613383	0.885217541
RPS11	0.035646451	0.035700417	0.886016036
C1RL	0.208669787	0.025737778	0.886242199
PTGES3	0.020628521	0.016463467	0.886394147
GSK3A	0.000115936	5.53724E-05	0.886553597
ISOC1	0.048532948	0.007324834	0.88759689
EMC6	0.032674779	0.006864031	0.888446777
AC074117.1	0.046135816	0.043982682	0.888464294
EMC4	0.003948253	0.004881412	0.888608031
WDR72	0.031449266	0.041328052	0.888741422

TMEM170A	0.044257407	0.075021892	0.888973985
SBDS	0.006975954	0.002129703	0.889560582
SASH1	0.086717627	0.038744604	0.889701483
ATP6V1G1	0.00531204	0.00267874	0.889745115
ZNF317	0.040765338	0.02113729	0.890018026
DDIT4	0.04273565	0.042692271	0.890558096
LRRC27	0.029900313	0.041875054	0.891444652
AL133453.1	0.002131305	0.002205448	0.891752849
RBPMS	0.087014845	0.043081953	0.892740078
POFUT1	0.027647092	0.059386089	0.893331737
HDGF	0.01891064	0.017719323	0.893958757
ROCK2	0.123945895	0.041336989	0.89415634
LONP2	0.02129393	0.007388613	0.894226553
ORMDL2	0.062608606	0.017859343	0.894382514
ITGB1	0.014027855	0.001544267	0.895394525
PPP1R9A	0.078142914	0.036775047	0.895707808
STARD3	0.038323761	0.01794664	0.896310903
LINC00526	0.016478379	0.082914837	0.896348562
ARL4C	0.005712116	2.1583E-06	0.896568409
BAZ1A	0.00060313	0.001998961	0.897302271
GBP2	0.015429783	0.004903051	0.897722163
ZKSCAN4	0.047537361	0.099594649	0.897918256
PERP	0.021961112	0.000459293	0.898230479
RMND5B	0.009111277	0.016074628	0.899143033
RUNX2	0.076855128	0.020758182	0.899246331
CXCL2	0.027882093	0.15275812	0.899607934
GRB2	0.01716952	0.040481488	0.900081177
SF3B6	0.028647526	0.016966073	0.900092868
LETM1	0.022619405	0.030691608	0.90072185
MOGS	0.00987244	0.003237727	0.901020335
MRPS6	0.019340328	0.015650535	0.901113505
DDX27	0.005136443	0.001974125	0.901808391
C9orf78	0.043483489	0.041745119	0.901989539
DDX39B	0.039726753	0.04572466	0.902971278
MAP2K7	0.044679919	0.044686235	0.903355776
MLXIP	0.020402076	0.005174539	0.903621272
LLGL2	0.012144572	0.001978089	0.904629413
SLC50A1	0.001626207	0.002878866	0.904787781
NUP214	0.058142638	0.032224283	0.905254852
WAPL	0.037516435	0.021040562	0.905325196
NR3C1	0.099034086	0.048540241	0.906108404
COG3	0.0414493	0.067083566	0.90621338
RPL7	0.121573064	0.049594745	0.906707793

YAE1D1	0.02549464	0.015570061	0.907292953
BORCS6	0.04805396	0.056862976	0.907332451
TRAF3IP2	0.010988215	0.007793526	0.907390306
C6orf226	0.02118702	0.020464701	0.907611789
DDX60	0.003107509	0.002918127	0.908799354
PHACTR4	0.002303992	0.016848813	0.909054107
GABRE	0.043543043	0.033119824	0.909067721
CLSTN1	0.016799394	0.011272173	0.909229095
IGLC2	0.03952443	0.065725628	0.910391252
PEX2	0.017320022	0.043598203	0.910412882
ACTA2	0.022311669	0.014785404	0.910449028
AP2A1	0.054088511	0.044835256	0.910641225
SNRPA1	0.019900144	0.020396835	0.911174872
ZC3HAV1	0.000698887	0.000624763	0.911566769
RTN4IP1	0.01910085	0.126972876	0.912007834
HBB	0.041043502	0.050799283	0.91280833
MELTF	0.031529404	0.034000861	0.913200044
CAST	0.012433838	0.008741771	0.913717171
DVL1	0.000265183	1.00743E-06	0.913943309
CSTF1	0.021018323	0.013387166	0.913953725
MIA3	0.061698465	0.035616244	0.914306892
STK19	0.018642907	0.007397585	0.91482556
TAPBP	0.002743243	0.002990515	0.91502242
DNAJB14	0.011475289	0.012691341	0.915603338
ETV3	0.000868058	0.000769928	0.915790222
DUSP18	0.026516944	0.025617486	0.915937014
LEPROTL1	0.035753392	0.009835659	0.917484246
MBTD1	0.034232484	0.00886298	0.917607299
HNRNPM	0.009409408	0.002994046	0.917780822
WASHC3	0.000993962	0.001880795	0.918594966
PEAK1	0.005308572	0.001258632	0.919323877
SMG1	0.020518846	0.028663862	0.91941574
RIPK2	0.04359954	0.03326369	0.919569327
EWSR1	0.004018387	0.002554474	0.919573528
EIF3H	0.009321882	0.002730767	0.919628773
PPRC1	0.031347113	0.045985575	0.920724415
AC073111.5	0.034113679	0.075282134	0.92218265
ZNF780A	0.135562538	0.033793069	0.923282063
PSMC1	0.021355587	0.028479474	0.923422769
SYNJ2	0.001046778	0.003594884	0.923567445
MOCS3	0.043726412	0.112109775	0.923747588
MAML2	0.024104302	0.008192411	0.924038572
MZF1	0.068417061	0.044104804	0.924350456

ETV6	0.001149822	0.006168086	0.924388345
ZDHHC9	0.023082499	0.026324095	0.924397127
COPB2	0.030607181	0.017051983	0.924403075
DICER1	0.024088116	0.01543019	0.924717244
KDM5A	0.108184731	0.049564121	0.924831273
DDA1	0.005849029	0.009165507	0.92494684
RPS27L	0.03943196	0.024976584	0.925430906
SLC12A6	0.012201538	0.009579596	0.925554464
ERBB3	0.003007141	0.003608561	0.927503546
EPPK1	0.001676456	4.45119E-05	0.927714226
SWI5	0.026903683	0.116050408	0.927721691
PLPP3	0.06173571	0.009057278	0.927845185
DCAF7	0.005624133	0.010326298	0.927871225
ZNF451	0.029647496	0.017609475	0.927906325
CD47	0.030220253	0.017219155	0.928222499
FNBP4	0.00749504	0.015397056	0.928345913
TAOK3	0.022096301	0.008400539	0.928576238
RTCB	0.054166924	0.018779355	0.928976071
ARID1B	0.021538645	0.016611017	0.929243869
DCP1A	0.004345005	0.023498829	0.931281044
CAV2	0.071601903	0.045266655	0.93155116
GTPBP6	0.070325902	0.04975468	0.931774983
CEP104	0.00184044	0.008196281	0.931818633
FXYD5	0.006562124	0.003419969	0.933359902
EHBP1L1	0.027598852	0.006164065	0.93433827
CABIN1	0.027214439	0.025917236	0.934467005
ZBTB20	0.013780114	0.035890939	0.934743313
VTI1B	0.027385021	0.010760365	0.934880168
OTUD1	0.008821132	0.005317909	0.935453876
DST	0.012802557	0.000888734	0.935861356
PCM1	0.003594682	0.00355208	0.935886916
ERAP1	0.015094061	0.000887041	0.936098639
ZCCHC3	0.032817882	0.061098726	0.936278949
OAF	0.072631389	0.0396982	0.936320501
EIF1B	0.007778713	0.004324106	0.938111029
HNRNPK	0.008549245	0.000951555	0.938166974
METRN	0.00155388	0.002299155	0.938564564
NDUFC1	0.026336867	0.046831893	0.939140546
ESCO1	0.028919378	0.053190338	0.939309054
TUBB4B	0.003989802	0.001481042	0.939637522
C12orf49	0.011819585	0.013200478	0.940062497
MAP7D1	0.061347396	0.049022033	0.940405913
CETN3	0.041473977	0.126859323	0.940454764

TRIR	0.001015425	0.000177008	0.940625871
PRDM4	0.025809489	0.02717248	0.942212811
PHF23	0.015931314	0.054735603	0.94280266
CDK12	0.025979507	0.047626279	0.943113109
BAK1	0.002741969	0.000682589	0.943182993
TMEM94	0.001354675	0.108955952	0.94328192
DOCK9	9.2746E-05	0.011553578	0.944554484
PIGT	0.006151033	0.002073735	0.94518593
AKAP12	0.233187454	0.003613511	0.945722017
DPF2	0.033498049	0.03277899	0.946773386
AGPAT1	0.048577333	0.096333844	0.948365818
STIMATE	0.038281802	0.024691955	0.949059642
AP2M1	0.010710006	0.003906799	0.949775491
ACSL3	0.019438515	0.013171876	0.950694581
HAGH	0.013702638	0.022393551	0.951294099
TRAK2	0.015369202	0.067195576	0.951870833
LINC01876	0.022362175	0.100360168	0.953619619
PPP2R5A	0.012813519	0.018666046	0.953769734
TXNRD1	0.004929901	0.002315963	0.953844912
TMEM164	0.04808885	0.05710439	0.954280357
CFLAR	0.000212451	0.000141335	0.955704202
OVOL1	0.088576333	0.017129704	0.956320488
LY6E	0.070394534	0.035916972	0.956566408
MSX2	0.04899585	0.006823795	0.95670995
TNIP2	0.004874085	0.000699309	0.957501987
TBCK	0.028249283	0.026972157	0.957751865
UFC1	0.09812717	0.037146205	0.957905989
FXR2	0.037108006	0.032476501	0.959306689
BLOC1S4	0.03346107	0.038335882	0.959715979
BCL9L	0.008731826	0.005864657	0.959790724
EMC3	0.017009704	0.024509893	0.960131221
GALNT2	0.008002213	0.005950131	0.96079513
POLDIP2	0.07099723	0.030565107	0.961654573
ARFGAP3	0.008646424	0.002324595	0.962082639
ZSWIM1	0.047993983	0.040703666	0.962472313
CRY1	0.004623864	0.003349159	0.962475539
TMEM161B	0.001447365	0.001887941	0.963069177
SMOX	0.03854792	0.034124369	0.963315921
RXRA	0.00434628	0.000306743	0.963330746
AC139530.1	0.028270626	0.036295047	0.964109951
TPRKB	0.008496681	0.01286264	0.964178571
BIK	0.001159772	2.17686E-05	0.964240073
INPP5D	0.03518917	0.023871183	0.964651561

ISG15	0.043298828	0.000835487	0.96466485
PLK2	0.03472967	0.047860524	0.964860932
GPBP1	0.011260129	0.019423606	0.96544033
EIF4E	0.078326287	0.042101589	0.966591128
EIF2S1	0.03130196	0.010304801	0.966782583
MAP3K2	0.023580206	0.015829686	0.967235803
PPFIBP1	0.058192271	0.04188122	0.967639879
ARHGAP42	0.016834656	0.00750861	0.968827918
MGAT2	0.032370993	0.032896982	0.968926793
TRIM44	0.001836813	0.010288564	0.968948818
DDRGK1	8.04128E-05	0.008361762	0.96896139
ALDH3A2	0.013919524	0.015670535	0.969130577
NOCT	0.028585265	0.02444807	0.970047583
LINC00543	0.026784214	0.01794646	0.971115522
B3GALT5	0.049299794	0.000577069	0.972789743
YIPF3	0.035130001	0.023334056	0.973202302
GBP1	0.036829074	0.0341361	0.974477114
U62317.2	0.044331655	0.066562187	0.975040806
H3F3A	0.088043182	0.043678499	0.97522657
PEX6	0.072849305	0.039927417	0.976379452
IL13RA1	0.001396076	4.71392E-05	0.976727506
TMED9	0.044054339	0.03788905	0.977126694
C11orf24	0.022651806	0.026680874	0.977765868
PXMP4	0.022065367	0.000978856	0.980105912
NDUFB4	0.057259303	0.03795507	0.980439724
GUK1	0.020218975	0.008129839	0.980460411
CLDN10	0.029584137	0.053364576	0.981289013
SMIM24	0.169994086	0.015360719	0.98164527
MBD3	0.026619645	0.036726552	0.982063429
IBA57	0.048121845	0.029863783	0.983261216
KCTD2	0.017598488	0.000977126	0.98326853
RHOG	0.008372151	0.001624951	0.983573658
CLASP1	0.003890926	0.002290714	0.984248512
TRIM56	0.045265098	0.012515906	0.984456439
IK	0.012656441	0.017189953	0.984557521
SAT1	0.000380547	0.000350736	0.985262326
NME7	0.028822092	0.008052292	0.985666859
SUV39H2	0.043336949	0.082885087	0.986202886
AL451165.2	0.085964196	0.015757528	0.986401589
TOR1AIP1	0.007842885	0.003142075	0.986448821
ACER3	0.010239126	0.010986256	0.986620867
LRIF1	0.093003539	0.045524561	0.987149754
SGF29	0.114516859	0.028428173	0.987801088

SP3	0.007677376	0.005699785	0.98847427
RNF121	0.01426651	0.000635365	0.988743717
TGFBR1	0.005431107	0.003162772	0.988824811
CHMP5	0.006002885	0.002217116	0.988835407
MIOS	0.061041884	0.032644535	0.988887646
MON1B	0.048963678	0.042888499	0.98946781
CDK11A	0.021994866	0.038930473	0.989531973
EMC7	9.55832E-05	7.69889E-05	0.990038305
UBC	0.001266391	8.91591E-05	0.99111981
ARHGEF7	0.002429998	0.000599658	0.991790957
CTR9	0.002972632	0.007813348	0.991808136
ARIH1	0.023100665	0.021841077	0.99218795
ZSCAN16-AS1	0.043415172	0.120479899	0.992237657
BCL7C	0.045068701	0.040442738	0.99226354
SLCO4A1	0.063770904	0.01801473	0.992399836
SUN2	0.008561165	0.00180994	0.992923189
GEMIN6	0.044225596	0.014083083	0.993702968
TMBIM6	0.000262599	0.000339635	0.993779941
RNF214	0.069515349	0.036283732	0.994253352
XRCC1	0.074190643	0.040033917	0.994631163
CLPTM1L	0.026139855	0.011803457	0.995051966
FAT1	0.015619395	3.39336E-06	0.995116478
ICE1	0.060163625	0.043451604	0.995333895
PSMD4	0.013550427	0.009317946	0.995500259
CCNG1	0.03617777	0.034901743	0.995831886
TRABD	0.015001256	0.002423848	0.995888
CASP5	0.037178091	0.035599171	0.996097971
IDI1	0.055140095	0.016383539	0.996116814
INPP5K	0.013171103	0.015328553	0.996554357
MLLT3	0.038491541	0.012430116	0.996884088
SNTB2	0.021117736	0.086098353	0.997262263
RALY	0.049822844	0.043973745	0.99764851
MEGF9	0.058385337	0.020155642	0.997938749
GTF2E1	0.000676036	0.003684845	0.998387129
COX6B1	0.002613661	0.000555475	0.998771116
DPY19L4	0.001167761	0.001195272	0.998926593

Table S3. Primer sequence information

Target gene	Orientation	Sequence
TGFβ1	Forward	GTACCAGAAATACAGCAACAATTCC
	Reverse	GGTGACATCAAAAGATAACCACTCT
WNT5B	Forward	GTGGCTACAACCAGT TCAAGAG
	Reverse	ATTTACAGATGTACTGGTCCACGAT
CA2	Forward	GATGGACAAGGTTCAGAGCATAC
	Reverse	ATCCAGCACATCAACAACTTTC
CEL	Forward	AAGACCTACGCCTACCTGTTTTC
	Reverse	TACTGAATGTCATCTGCATGGTC
GAPDH	Forward	TTGTCAAGCTCATTTCCTGGTATG
	Reverse	TCTCTCTTCCTCTTGTGCTCTTG
